# I_2_/DMSO-mediated oxidative C–C and C–heteroatom bond formation: a sustainable approach to chemical synthesis

**DOI:** 10.1039/d3ra08685b

**Published:** 2024-02-14

**Authors:** Rakshanda Singhal, Satya Prakash Choudhary, Babita Malik, Meenakshi Pilania

**Affiliations:** a Department of Chemistry, Manipal University Jaipur VPO- Dehmi-Kalan, Off Jaipur-Ajmer Express Way Jaipur 303007 Rajasthan India meenakshi.pilania@jaipur.manipal.edu

## Abstract

The I_2_/DMSO pair has emerged as a versatile, efficient, practical, and eco-friendly catalyst system, playing a significant role as a mild oxidative system, and thus employed as a good alternative to metal catalysts in synthetic chemistry. Presently, I_2_/DMSO is a thriving catalytic system that is used in preparing C–C and C–X (X = O/S/N/Se/Cl/Br) bonds, resulting in the formation of various bioactive molecules. Many processes utilize this system, including *in situ* glyoxal synthesis by diverse sp, sp^2^, and sp^3^ functionalities *via* iodination and subsequent Kornblum oxidation. Focusing on oxidation processes, this study examines the synergistic effect of dimethyl sulfoxide (DMSO) and molecular iodine in improving synthetic techniques. We provide a comprehensive overview of the research progress on the I_2_/DMSO catalytic system for the formation of C–C and C–heteroatom bonds from 2018 to the present. Additionally, the future prospects of this research field are discussed.

## Abstract 2

A variety of bioactive compounds can be synthesized *via* C–C and C–X (X = O/S/N/Se/Cl/Br) bond formation using an efficient catalytic system I_2_/DMSO. This review highlights the progress and identifies potential paths for future research in this field.

## Graphical abstract

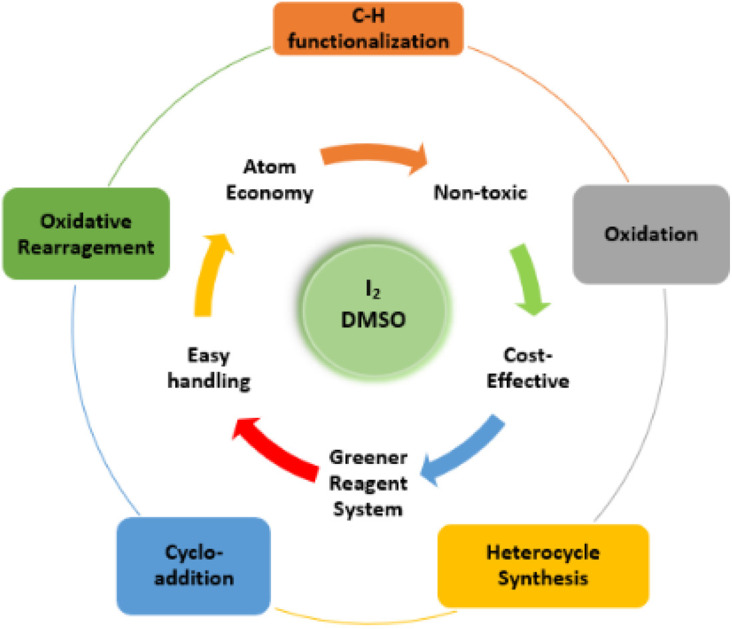

## 1. Introduction

In the field of synthetic chemistry, researchers have consistently strived to develop reaction approaches that have the characteristics of environmental sustainability, economic viability, safety, and effectiveness. As part of this endeavor, they developed and utilized cost-effective, environmentally sustainable catalysts and solvents. In order to meet the above-mentioned requirements, the combination of I_2_/DMSO as a catalyst and solvent has emerged as a versatile approach.^1,2^ Molecular iodine (I_2_) has gained significant popularity as a catalyst and reagent due to its notable attributes,^3–5^ including its cost-effectiveness as an alternative to transition and rare earth metals, its environmentally friendly nature, non-toxicity, high stability, low sensitivity to moisture, ease of handling, and notably its exceptional performance across a wide range of reaction mixtures, including highly concentrated and solvent-free systems.^6^ Collectively, these characteristics make I_2_ a versatile and unique catalyst and reagent.^7,8^ Iodine is present in numerous types of cyclic and acyclic reagents in various oxidation states and cyclic and acyclic forms. Since its discovery in 1811,^9–11^ I_2_ has been shown to be a better alternative to metal catalysts due to its high efficiency as a reagent in cross-dehydrogenative coupling (CDC). Furthermore, its use dramatically reduces the number of reaction steps and produces a higher yield in multicomponent reactions (MCRs) and shortens the time for the synthesis of C–C, C–X, and C-Het bonds.^12–17^ Thus, molecular iodine (I_2_) works perfectly in MCRs, CDC, and in various tedious and hard-to-pursue reactions, providing an easy approach to the synthesis of various high-value bioactive, pharmaceutical, and industrial compounds.^18^ Dimethyl sulfoxide (DMSO) is a well-known aprotic polar solvent, which is less toxic than similar solvents. DMSO is used equally in chemistry and biological activities such as polymerase chain reactions (PCR), cryoprotectants, and integral parts of some drugs and medicines. In chemistry, DMSO is generally used as an inexpensive, high-boiling, and innocuous solvent. Besides, it has been utilized as a mild oxidant, methylthiolating, formylation, and cyanation agent, and as a synthon for C–S bonds.^19,20^ DMSO acts as a mild oxidant and plays a pivotal role in Pfitzner–Moffatt oxidation, Swern oxidation, Corey–Kim oxidation, Corey–Chaykovsky epoxidation, and Kornblum oxidation. Furthermore, it also functions as a terminal oxidant, eliminating the need for transition metals.^21^ Fascinatingly, the combination of these two readily accessible and benign molecules presents new avenues for simple, inexpensive, facile, eco-friendly, metal-free, and atom-economical reaction strategies, facilitating simple oxidation to chemoselective and regioselective oxidation, sulfenylation, and amination to effectively generate C–C, C–X, C–O, C–S, S–N and C–N bonds, affording various bioactive, pharmaceutical, industrial, and applied molecular structures.^22–26^ This review presents a detailed summary of the research advancements achieved in the I_2_/DMSO catalytic system for the formation of C–C and C–heteroatom bonds from 2018 to now. Furthermore, the potential future development in this research field is discussed.

## 2. Kornblum oxidation

**Fig. 1:**
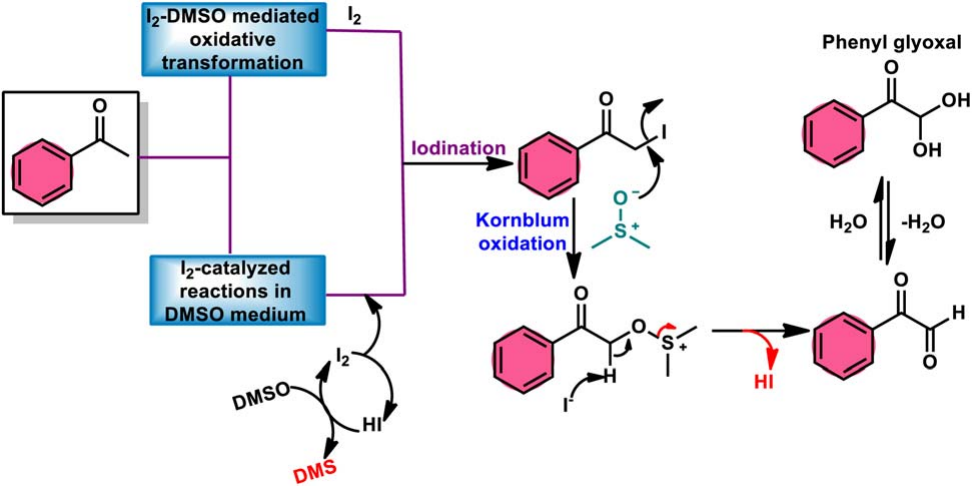
Schematic illustration of Kornblum oxidation.

Fascinatingly, the I_2_/DMSO duo is very suitable for chemoselective oxidation, dehydrogenation, oxidative aromatization, protection/deprotection of various functional groups, and regioselective and stereoselective transformations.^27–29^ The I_2_/DMSO system has been extensively reported for its dual functionality, as follows: (i) the facilitation of I_2_-promoted activities in DMSO medium and (ii) oxidative changes *via* the interaction between I_2_ and DMSO.^30–33^ This method includes initial iodination of substrates including various sp, sp^2^, and sp^3^ functionalities in the presence of molecular iodine. This method proceeds *via* Kornblum oxidation in both instances (Fig. 1).^34–36^ Kornblum oxidation is a chemical process involving the transformation of a primary halide to an aldehyde, which is facilitated by the use of dimethyl sulfoxide (DMSO) as a reagent, also leading to the synthesis of dimethyl sulfide (DMS) as a byproduct.^37–40^ It is worth noting that this oxidation reaction is commonly conducted at elevated temperature. Besides being only a mild oxidant, I_2_ with K_2_S_2_O_8_/DMSO can be employed for the deoxygenation of *N*-oxides and sulfoxides,^41^ sulfonyation,^42,43^ and asymmetric alkoxy selenylation.^44^ Furthermore, it provides the advantage of the selective oxidation of alcohol to carbonyl or carboxylic acid. However, the application of H_2_O_2_ as an external oxidant reduces the amount of I_2_ reconverting iodide, whereas the addition of TBHP (*tert*-butyl hydroperoxide) to I_2_/DMSO provides opportunities to obtain various significant compounds such as benzimidazo[1,2-*c*]quinazolines^45–47^ and amidation.^7,48^ In general, the reaction mechanism of I_2_/DMSO is straightforward and unambiguous. It initiates reactions by iodinating alkenes/alkynes/carbonyls, which subsequently undergo further oxidation to form phenylglyoxal *via* Kornblum oxidation.^49^ In conclusion, the I_2_/DMSO duo provides beneficial and effective options to prepare various highly applicable molecules and their derivatives such as quinazoline, quinolines, isatins, thiadiazoles, triazolopyridines, trisubstituted imidazoles, benzothiazoles, benzothiadiazines and beznothiozole-fused imidazoles.^50,51^

**Fig. 2:**
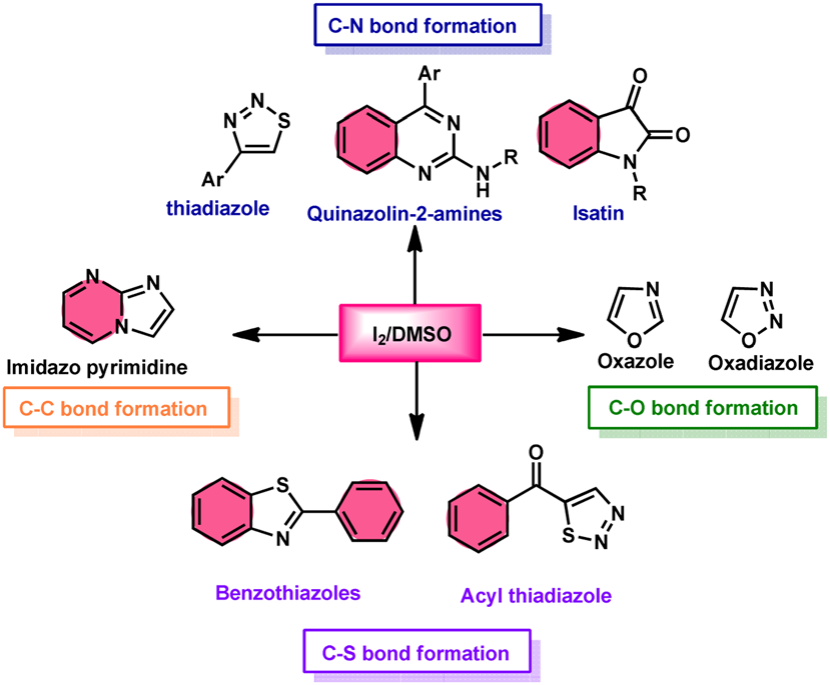
I_2_/DMSO role in C–X bond formation.

Additionally, this procedure can facilitate the basic formation of C–C, C–N, C–O, and C–S bonds using metal-free,^52^ atom-and-step effective, and environmentally friendly chemistry (Fig. 2). We attempt to highlight I_2_/DMSO-based studies reported from 2018 to 2022, showing its abundant synthetic abilities such as oxidative amination, amidation, sulfonation, sulfenylation, esterification, etherification to aryl alkyl ether, and dicarbonylation of C–H (sp^3^,sp^2^, and sp) bonds.^53^

## 3. Six-membered ring formation with one-heteroatom

**Scheme 1:**
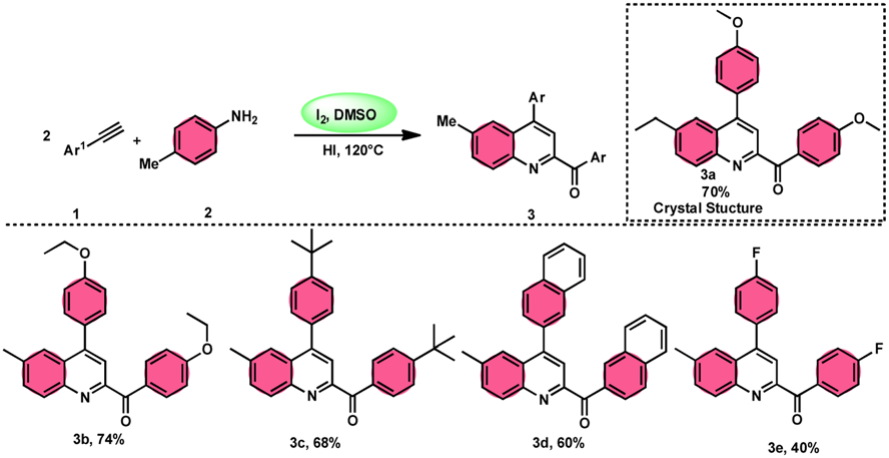
Preparation of substituted quinolines 3 and its mechanism.

**Scheme 2:**
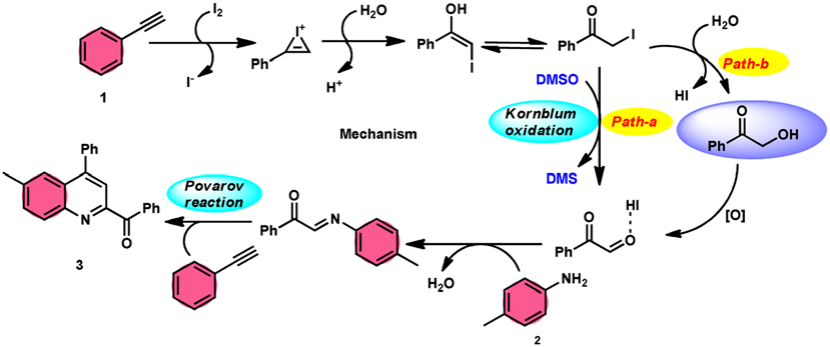
Mechanism for the synthesis of substituted quinolines 3.

The I_2_/DMSO system-based synthesis of substituted quinolines 3 was demonstrated by Yan-Dong Wu, An-Xin Wu, and colleagues.^54^ The synthesis of 2,4-substituted quinolines 3 was achieved through a new I_2_-mediated Povarov reaction involving aryl acetylenes 1 and anilines 2, wherein aryl acetylene 1 initially functions as both a dienophile and diene precursor (Scheme 1). The types of suitable diene precursors have been substantially expanded by this advancement in the Povarov reaction and its substrate range has been extended to carbonyl compounds. When used in the process, aryl amines with both electron-donating and withdrawing groups produced product 3 in satisfactory yield. According to initial mechanistic research, the I_2_/DMSO approach achieved the oxidative carbonylation of the aryl acetylene C(sp)–H before undergoing a [4 + 2] cycloaddition process. Initially, iodine activates phenylacetylene to produce an iodonium cation and release I^−^ species, which is subsequently attacked by water to produce an intermediate called an enol. The intermediate enol is isomerized to α-iodophenone at this point, following two paths. Path one involves the utilization of Kornblum oxidation, whereas in path two, the intermediate compound undergoes a reaction with water to produce 2-hydroxy acetophenone, which is further subjected to oxidation and the Povarov reaction (Scheme 2).

**Scheme 3:**
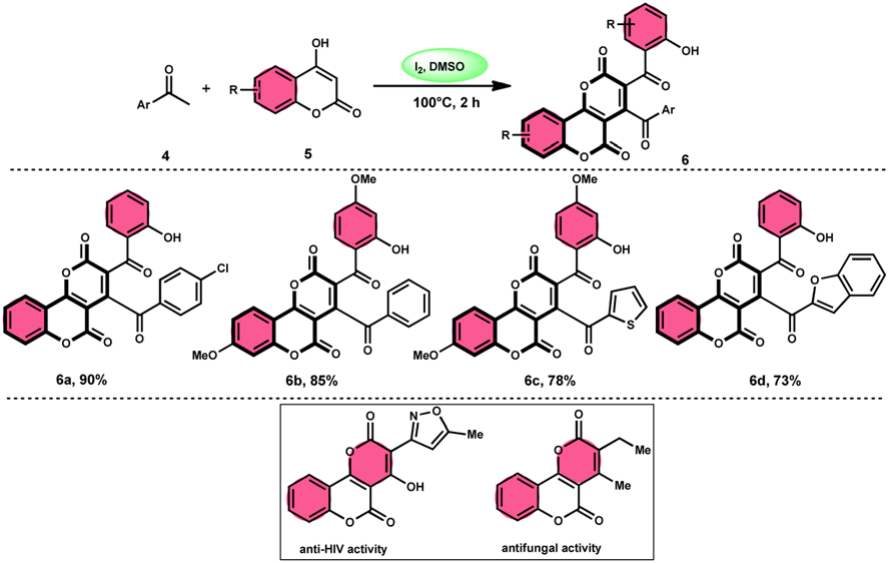
Synthesis of pyranochromene-2,5-diones 6.

**Scheme 4:**
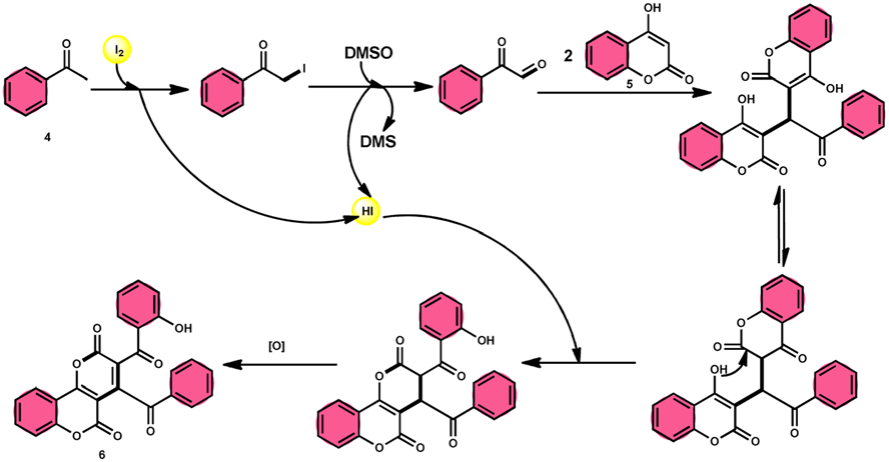
Mechanism for the synthesis of pyranochromene-2,5-diones 6.

In the study by Wu *et al.*,^55^ they provided a straightforward and efficient scheme for the synthesis of several substituted pyrano[3,2-*c*]chromene-2,5-diones 6. This strategy involves the I_2_-promoted sequential cyclization of readily available aryl methyl ketones 4 and 4-hydroxycoumarins 5, as depicted in Scheme 3. Based on initial investigations into the mechanism, it was determined that the reaction follows a sequential pathway involving iodination, annulation, and Kornblum oxidation. The HI generated in the I_2_–DMSO system exhibited significant promotive effects, effectively enhancing the rate of the annulation process. Pyrano[3,2-*c*]chromene-2,5-dione 6 is a highly desirable structural motif found in heteropolycyclic complexes. Its derivatives have significant medicinal capabilities, including substantial anti-HIV activity and antifungal activity.^56^ It was shown that the electronic characteristics of substituted 4-hydroxycoumarins 5 had minimal effect on the yields. The aforementioned process, which serves as a viable synthetic method, provides simple and efficient access to *o*-heteropolycyclic scaffolds under mild conditions (Scheme 4).

**Scheme 5:**
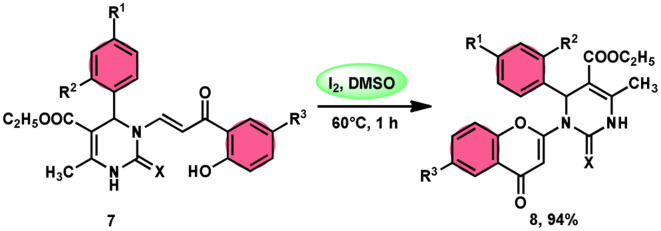
Synthesis of benzopyran-4-one 8 in I_2_–DMSO system.

Pathan, Ali, *et al.*^57^ devised a very efficient one-pot synthesis approach using an I_2_/DMSO green solvent. This methodology allows the rapid synthesis of 2-substituted 4-*H*-1-benzopyran-4-one derivatives 8, which are considered significant pharmacophores. The notable point in this methodology is that the researchers used the molecular hybridization scheme *via* the path of cyclodehydration motivating the α,β-unsaturated carbonyl group, which is a very effective approach. The desired products were not observed, even in trace amounts in the absence of the green catalyst I_2_, although in the plausible mechanism, the investigators did not explain the role of I_2_. The methodology is highly feasible given that the reaction is faster based on the green medium of I_2_/DMSO and requires very mild conditions. The arrays of the new product 5-(ethoxycarbonyl)-4-methyl-1-(6-methyl-4-oxo-4*H*-chromen-2-yl)-2-oxo-6-phenyl-1,2,3,6-tetrahydropyrimidine-1-sulfide 7 work as both antibacterial and antifungal in a single compound form. The researchers performed a detailed bioactivity and pharmacological study of the products employing XRD, Petra, Osiris, Molinspiration (POS) analysis and determined that the pharmacophore sites in the compounds were some substituents on the heterocyclic and aromatic moiety (Scheme 5).

**Scheme 6:**
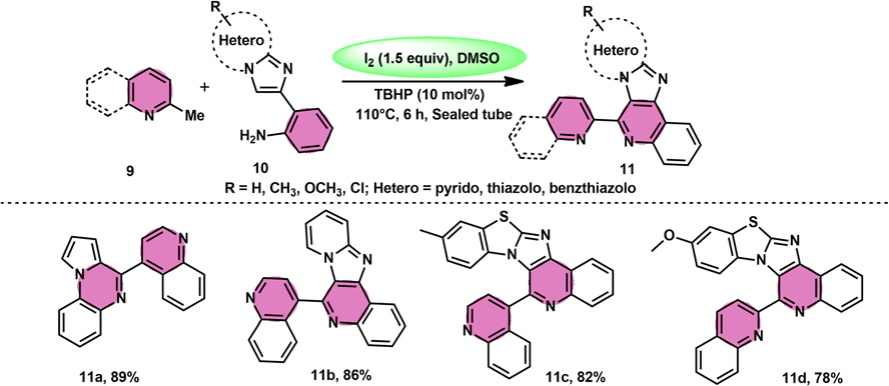
I_2_–DMSO-based synthesis of N-fused polyheterocycles 11.

**Scheme 7:**
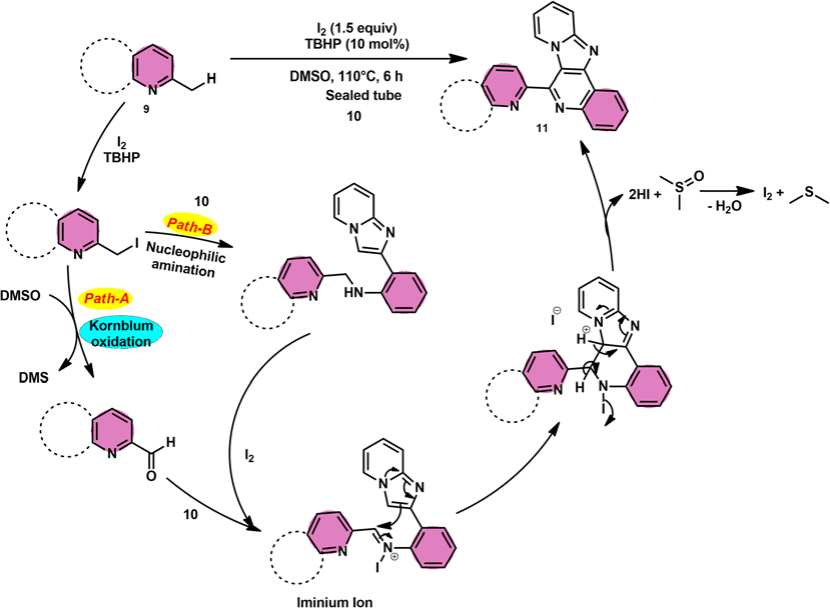
Mechanism for synthesis of N-fused polyheterocycles 11.

Subha Reddy and co-workers^58^ employed I_2_/DMSO and TBHP as a co-oxidant to develop a potent, one-pot, metal-free, atom-economical, simple reaction strategy to synthesize various bioactive N-fused polyheterocycle compounds 11 through the formation of C–C and C–N bonds (Scheme 6). This strategy has advantages compared to previous strategies owing to its simple path, atom economy, readily available starting materials, good yields, metal-free reagents and environmental-friendly nature. The synthesis of a range of bioactive compounds, including pyrido-/thizolo/benzthiozol-imidazo[4,5-*c*]quinolines and indolo-/pyrrolo-[1,2-*a*]quinoxalines 11a–11d, was achieved using 2-methylquinoline 9 and 2-(imidazo[1,2-*a*]pyridine-2yl)aniline 10, which are both readily available starting materials. The latter compound was also synthesized in the same laboratory. The role of TBHP is simply a co-oxidant, which shortens the reaction time drastically. The methodology was well tolerated by both substrates and its yields were appreciable. The following reaction mechanism starts with the iodination of the methyl group located on the 2-methylquinoline 9 compound. This is followed by the Kornblum oxidation process, resulting in the formation of the 2-carbaldehyde quinoline compound. The aldehyde functional group undergoes a reaction with the amine functional group of another substrate, leading to the development of an iminium ion. This is followed by a cyclization process *via* the carbon–carbon bond, ultimately yielding the expected product, as seen in Scheme 7.

**Scheme 8:**
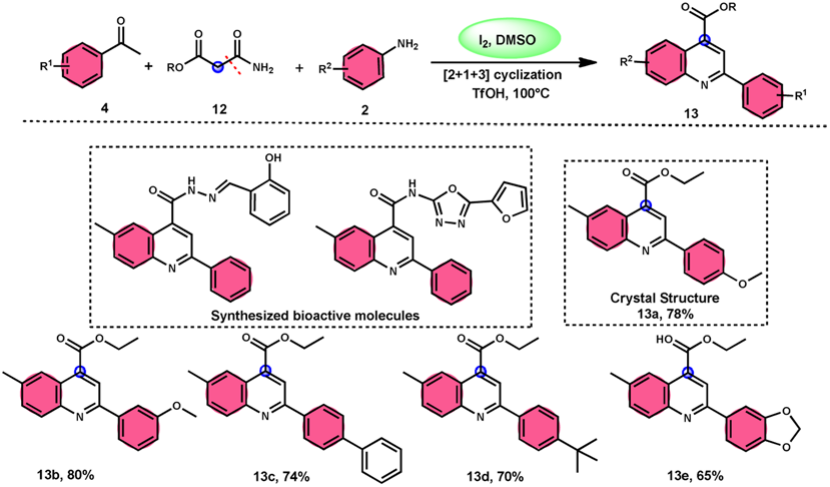
I_2_–DMSO system-based synthesis of 2-arylquinoline-4-carboxylate 13.

**Scheme 9:**
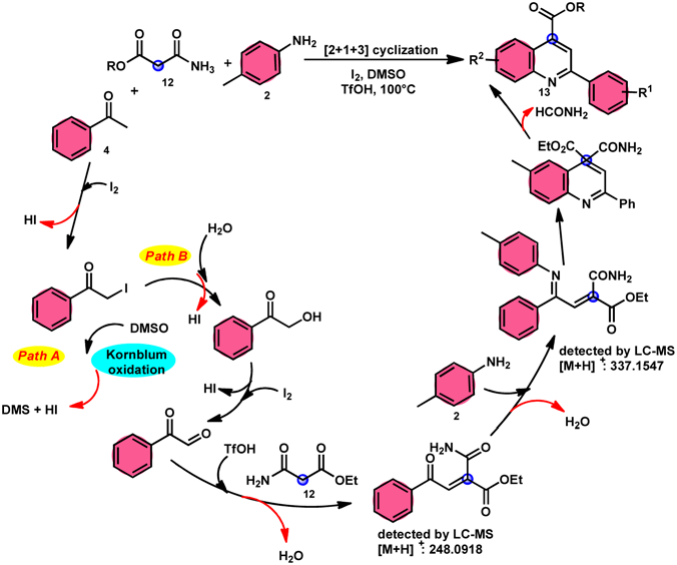
Mechanism of 2-arylquinoline-4-carboxylate 13.

2-Arylquinoline-4-carboxylate moiety 13, which is found in many drugs, natural products, and bioactive products, was prepared by An-Xin Wu *et al.*^59^*via* a unique, metal-free, straightforward, and simple reaction condition-based protocol using the I_2_/DMSO system in an acidic medium and aryl methyl ketone, 1,3-dicarbonyl, and aryl amines (Scheme 8). The reaction was conducted in a [2 + 1 + 3] manner, including the use of aryl methyl ketone 4, 1,3-dicarbonyl 12, and arylamines 2. The C–C bond of 1,3-dicarbonyl acts as a single synthon and breaks down during the process. Three bioactive molecules were prepared using this protocol, with good tolerance of various substituents on all three substrates, where even highly steric hindered arylamines 2 and aryl methyl ketones 4 were converted into the anticipated products with good quantity, showing the efficacy of the reaction. This research team proposed a reaction mechanism in which aryl methyl ketone 4 undergoes conversion into phenylglyoxal through the use of I_2_/DMSO. The first step of this mechanism involves the reaction between the aldehydic carbonyl group of phenylglyoxal and 1,3-carbonyl derivatives 12. This reaction leads to the production of a carbon–carbon double bond (C=C). Moreover, the ketonic carbonyl moiety present in phenylglyoxal undergoes a reaction with arylamine 2, resulting in the formation of a C=N bond. Subsequently, an intramolecular cyclization process occurs, leading to the synthesis of the intended product 13 (Scheme 9).

**Scheme 10:**
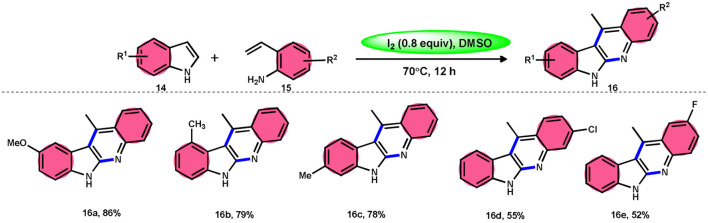
Synthesis of 11-methyl-6*H*-indolo[2,3-*b*] quinolines 16 and selected examples.

Wei, Li, and colleagues^60^ devised an easy one-pot method to produce a wide range of 11-methyl-6*H*-indolo[2,3-*b*]quinolines 16 (Scheme 10). This was achieved using an I_2_-mediated annulation process involving indoles 14 and 2-vinylanilines 15. This approach has several benefits compared with existing techniques, such as a wide range of applicable materials, gentle reaction conditions, and straightforward operation. Furthermore, this approach does not require any pre-functionalization protocols for the production of new C–C and C–N bonds and generates the necessary products in moderate to satisfactory quantities. In addition, these tetracyclic compounds were assessed for their antiviral and cytotoxicity potency against the EV71 and CVB3 viruses. The first observations indicated that some compounds showed remarkable antiviral properties against EV71 and CVB3. Additionally, these compounds efficiently suppressed virus-induced damage to cells and decreased the production of new viral particles. A potential mechanism was suggested. Initially, the reaction can only take place with 2-vinylaniline 15, which can combine with I_2_ to produce an iodonium ion. This is followed by 5-*endo*-cyclization and elimination of HI, resulting in the formation of indole 14.^61^ Alternatively, 2-vinylaniline 15 generates a stable benzylic carbocation C when exposed to a strong acid such as HI. This carbocation C is then targeted by indole 14, resulting in the formation of an iminium ion. Following the intramolecular cyclization of the compound, a new intermediate is formed.^62^ Product 16 is obtained by the oxidative aromatization of the intermediate using iodine. Throughout the procedure, iodine is continually regenerated by the oxidation of HI by DMSO.^1,63,64^

## 4. Six-membered ring formation with two-heteroatom

**Scheme 11:**
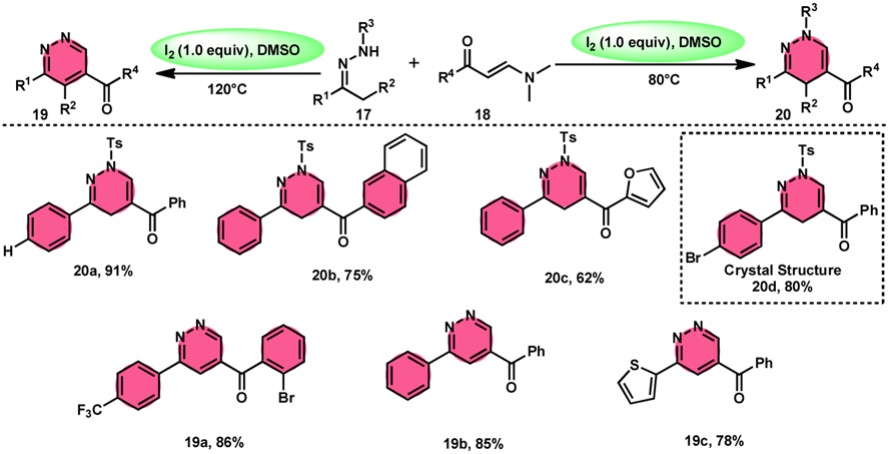
Synthesis of 1,4-dihydropyridazines 20, pyridazines 19 and representative examples.

**Scheme 12:**
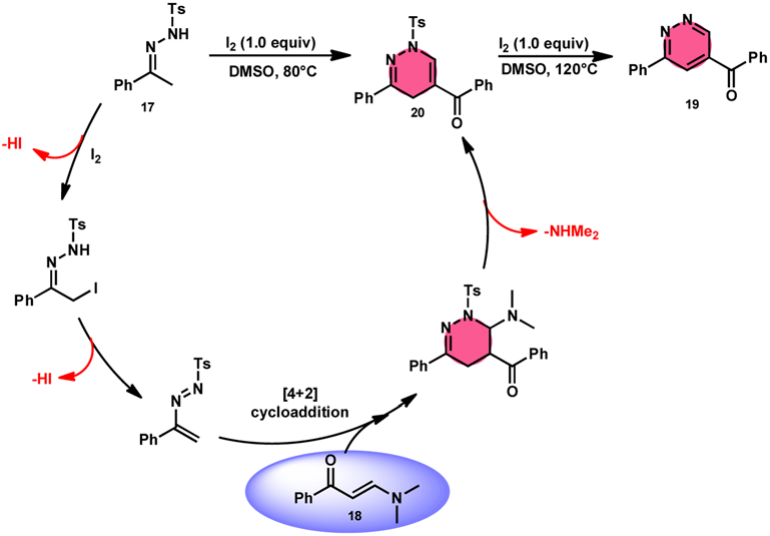
Mechanism for the synthesis of 1,4-dihydropyridazines 20, pyridazines 19 and representative examples.

Fei Huang and colleagues developed a direct and effective method for the synthesis of 1,4-dihydropyridazines 20 and pyridazines 19. This method involves the I_2_-promoted [4 + 2] cycloaddition of *N*-tosylhydrazone 17, which is produced *in situ*, with enaminones 18 (Scheme 11).^65^ The prudent choice of the reaction temperature is responsible for the change in selectivity. The primary advantages of this work include its selective and regulated synthesis, wide functional group tolerance, good to extraordinary reaction yields, absence of metals or bases, and one-pot method adaptability. The synthesis of the pyridazine *N*-oxide derivative and the experiment conducted on a gram-scale demonstrate the prospective use of the developed methodology. The metal/base-free methodology has significant qualities such as regulated and selective formation, extensive functional group compatibility, and suitable for one-pot procedures. Additionally, a control experiment without I_2_ was conducted, which failed to produce the intended result, demonstrating the importance of I_2_ in this reaction. However, the effectiveness of the reaction to produce 1,4-dihydropyridazine 20 was not improved by decreasing or increasing the temperature. Remarkably, an increase in temperature to 120 °C resulted in a significant improvement in the production of pyridazine compound 19, resulting in an isolated yield of 85%. This outcome provides evidence supporting the concept that elevating the temperature facilitates the removal of the –Ts group. The reaction exhibited no progress in the presence of nitrogen. Also, this process is unaffected by the electronic properties of the substituents attached to the phenyl ring of enaminones (Scheme 12).

**Scheme 13:**
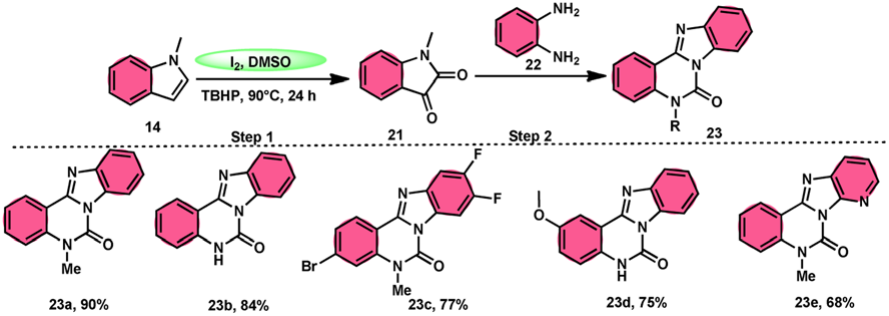
Reaction of isatin 21 and representative examples.

**Scheme 14:**
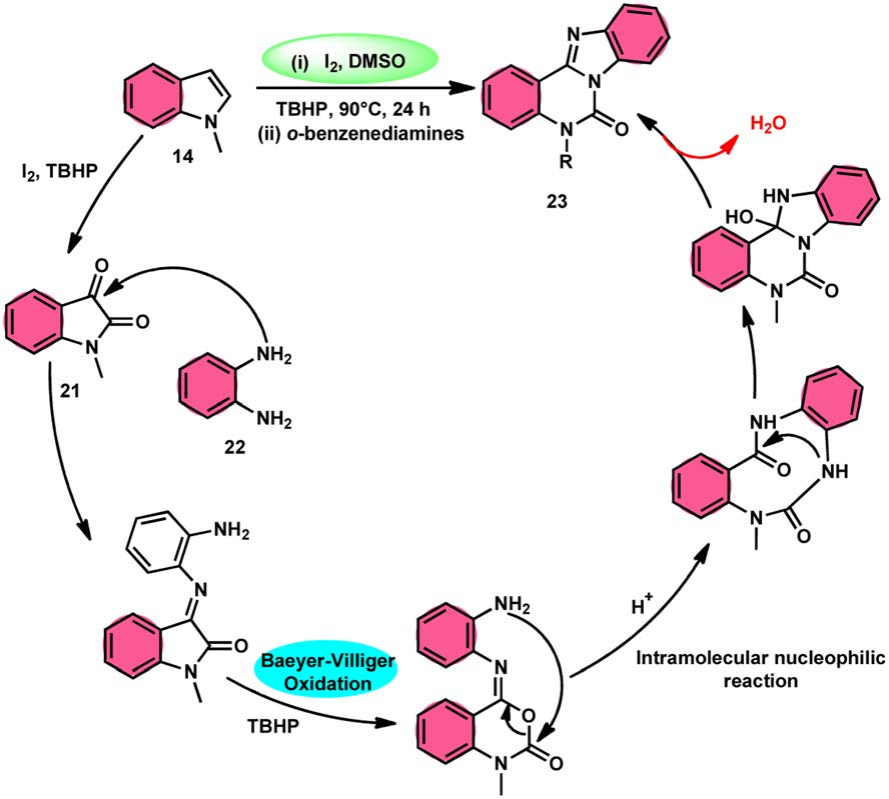
Reaction mechanism and representative examples.

Ma *et al.*^66^ conducted a study, whereby they devised a very efficient methodology for the synthesis of benzimidazo[1,2-*c*]quinazolin-6-ones 23 and their derivatives (Scheme 13). This process was performed under metal-free and mild reaction conditions, utilizing I_2_/DMSO and TBHP (tertiary butyl hydroperoxide). This reaction is very convenient and practical because the starting reactants indole 14 and 1,2-diaminobenzene 22 are readily available, good yields are obtained, and various substituents both on indole 14 and 1,2-diaminobenzene 22 are well tolerated. The methodology proceeds in two straightforward steps, where in the first step, the indole is oxidized to isatin 21, and in the second step the addition of *o*-benzenediamine 22 leads to the product. However, TBHP working as an oxidant is indispensable in the reaction, especially in the second step. The investigators proposed the reaction mechanism, which was supported by spectroscopic data. In the first step, indole 14 is oxidized to isatin 21. In the second step, quinoxaline is the expected product but the reaction proceeds through a ring-expansion mechanism *via* Baeyer–Villiger rearrangement mediated by TBH and results in the formation of benzimidazole. 1,2-Diaminobenzene 22 as a nucleophile reacts with isatin 21, followed by Baeyer–Villiger rearrangement, and in the last step, intramolecular nucleophilic reaction results in the formation of the product (Scheme 14).

**Scheme 15:**
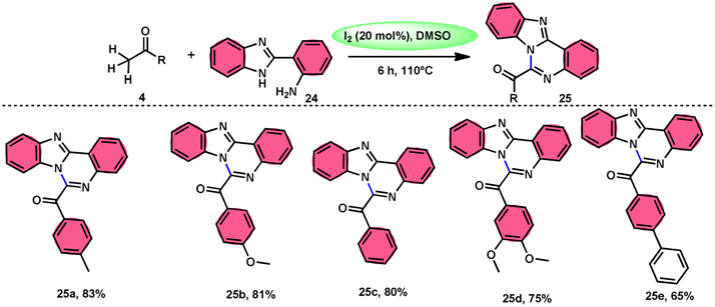
I_2_/DMSO-catalyzed synthesis of benzoimidazo-quinazolinones 25.

Padmini and coworkers^45^ prepared benzoimidazoquinazoles 25 derivates in a straightforward, one-pot, metal-free, effective new reaction methodology using the combination of I_2_ and DMSO. The developed technique can be considered feasible due to the fact that the I_2_/DMSO catalyst facilitates the oxidative amination of the C–H bond in ketones 4, resulting in favourable yields. This process demonstrated good tolerance when applied to both electron-donating groups (EDGs) and electron-withdrawing groups (EWGs) connected to aryl methyl ketones 4. Additionally, it relies on the use of conveniently accessible aryl methyl ketones 4. These ketones can be rapidly transformed into arylglyoxals by the α-iodination of the C–H (sp^3^) bond in the methyl group, followed by Kornblum oxidation. The proposed reaction mechanism proposed by the authors involves the use of I_2_ as a mediator for the α-iodination of aryl methyl ketone 4, which is followed by Kornblum oxidation. The series of chemical events described above results in the synthesis of arylglyoxal and the liberation of dimethylsulfide. The arylglyoxal compound undergoes a chemical reaction with 2-(1*H*-benzo[*d*]imidazole-2-yl) aniline 24, which acts as a nucleophilic reagent. This reaction forms an imine intermediate, which then undergoes intramolecular cycloaddition to provide a bioactive product (Scheme 15).

**Scheme 16:**
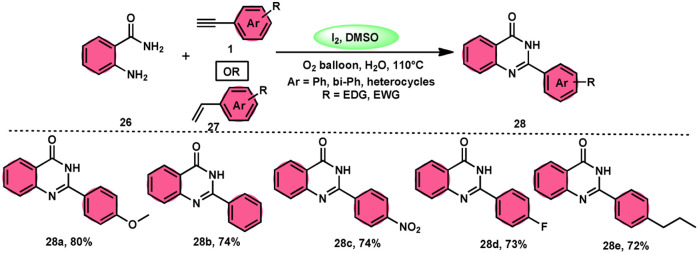
Synthesis of aryl quinazolinone composition 28 and some selected examples.

**Scheme 17:**
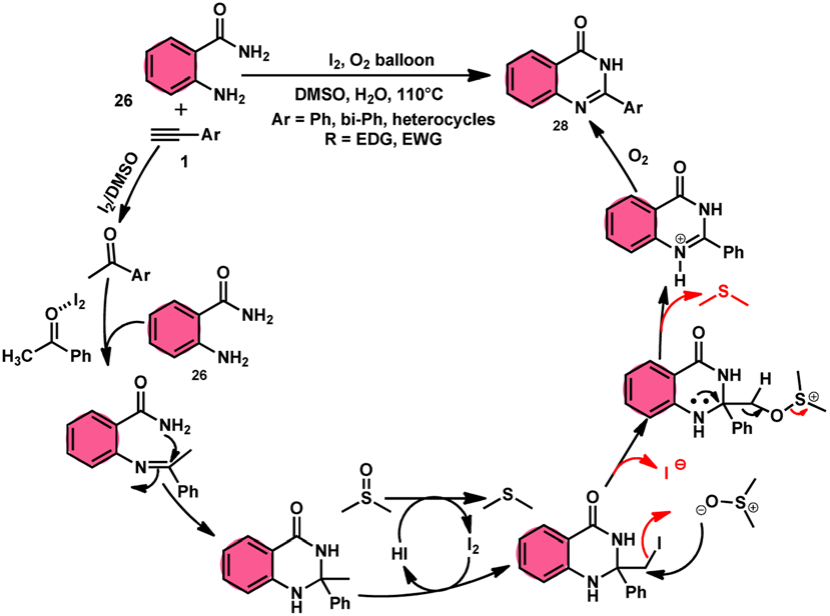
Mechanism for the synthesis of aryl quinazolinone 28.

Sandip B. Bharate *et al.*^67^ synthesized a new non-sulfonyl NLRP3 inflammasome inhibitor compound 2-arylquinazolin-4(3*H*)-one 28 and investigated its bioactivities, interactions, binding sites, and effectiveness employing molecular docking, computations, and other methods (Scheme 16). This method employed is very elegant, economic, metal-free, and molecular O_2_ works as the oxidant in I_2_/DMSO medium, although a higher temperature (140 °C) is required but its yields are good. The condensation between the reactants, namely, 2-aminobenzoamides 26 and terminal aryl alkynes 1 or styrene 27, which are easily accessible, results in the formation of quiozoline-4(3)-ones 28. The above-mentioned conversion takes place through the process of oxidative cleavage of unsaturated carbon–carbon (C–C) bonds, subsequently leading to the formation of two carbon–nitrogen (C–N) bonds. The first carbon–nitrogen (C–N) bond is formed using a Schiff base mechanism, but the subsequent C–N bond is generated *via* an intramolecular cyclization process. Phenylacetylenes 1 and styrene 27 with various EDGs and EWGs are converted into the desired products with good yield; however, aliphatic alkynes and DMAD (dimethyl acetylenedicarboxylate) are not suitable to lead the reactions. The researchers revealed that quinazolin-4-(3*H*)-ones 28 are potent NLRP3 inflammasome inhibitors by binding with the ATP moiety *via* H-bonding and calibrated the inhibitory concentration value of the 5 μM on the IC_50_ scale, which shows its good potency (Scheme 17).

**Scheme 18:**
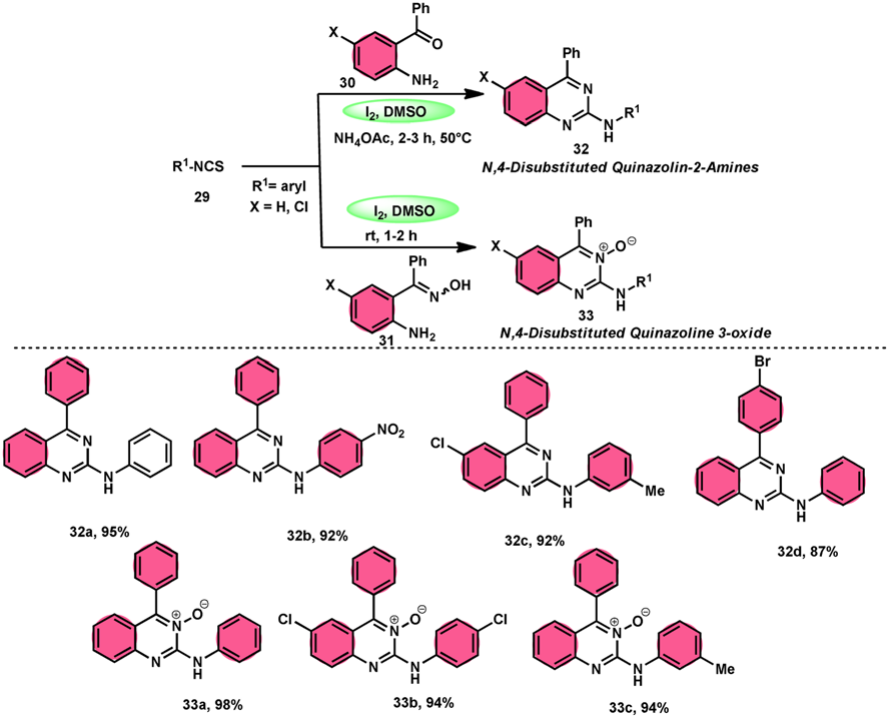
Synthesis of quinazolines 32, quinazoline oxides 33 and selected examples.

In the study by Krishna and Nagesh,^68^ they proposed a versatile methodology for the synthesis of *N*-4-disubstituted quanzoloin-2-amine 32 and 4-aryl-2-(arylamino) quinozoline-3-oxide 33. The approach used in this study involves the utilization of readily available and economically viable reagents, such as aryl isothiocyanate 29, 2-amino benzophenone 30, NH_4_OAc, and (2-aminophenyl)(phenyl) methanone oxime 31. The reactions are facilitated by the utilization of I_2_/DMSO as a promoter. The proposed methodology exhibits several advantageous characteristics, including environmentally sustainable features, cost-effectiveness, absence of transition metals, efficient time management, high production yield, and notable tolerance towards both electron-donating and electron-removing groups on phenyl isothiocyanates 29. However, it should be noted that alkyl isothiocyanates and 2-aminophenyl alkyl ketones are unable to achieve the desired outcomes within this framework. According to the plausible reaction mechanism, isocyanate moiety 29 is attacked by the amine group of the substrate, creating a C–N bond and producing a thiourea intermediate, which reacts with NH_4_OAc to afford the imine intermediate, and intramolecular cyclization attack by N of the imine intermediate on the carbon of the thiourea results in the formation of *N*-4-disubstituted quanzoloin-2-amine 32 (Scheme 18).

**Scheme 19:**
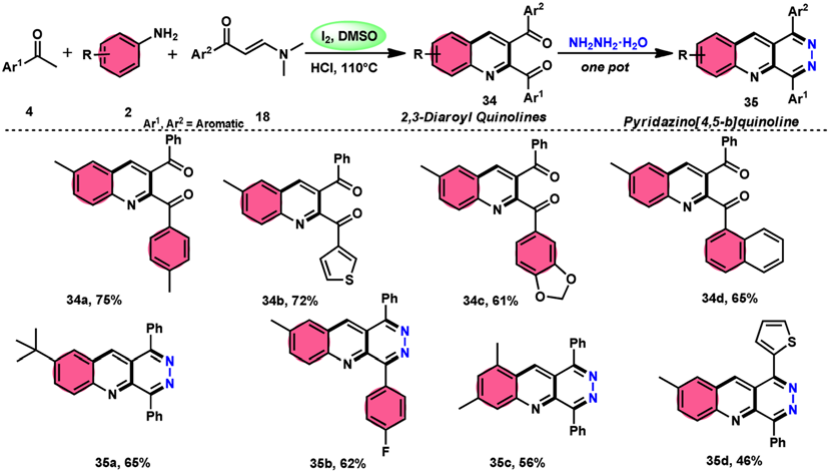
Synthesis of quinolines 34, 35 and representative examples.

**Scheme 20:**
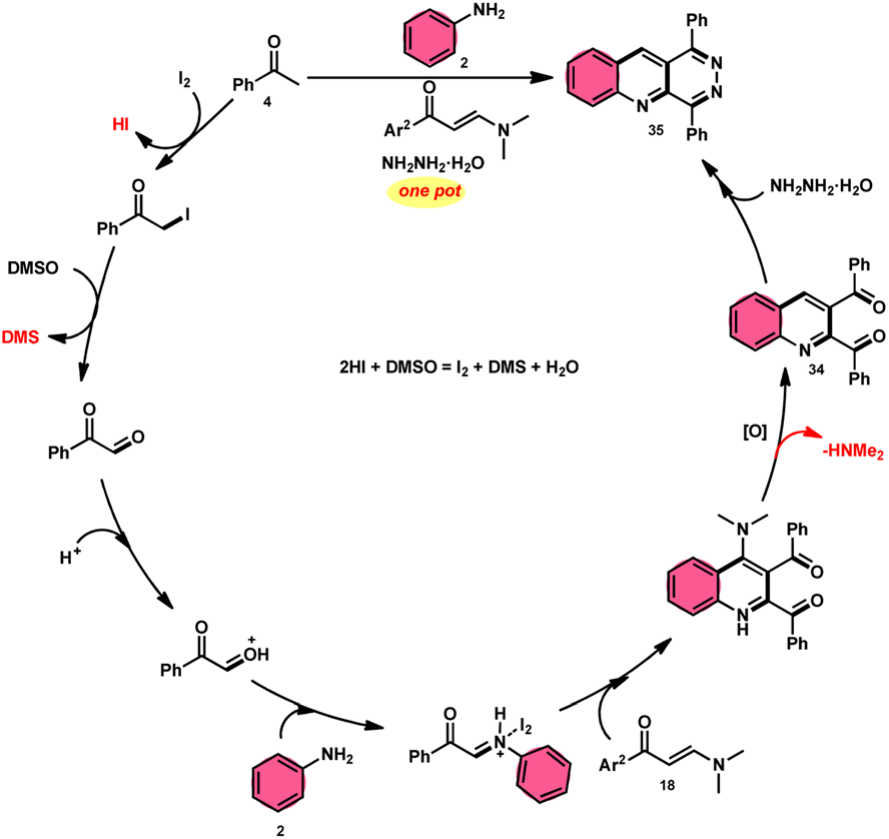
Mechanism for the synthesis of quinolines 34 and 35.

Wu *et al.*^22^ developed a successful strategy utilizing the well-known Povarov reaction to synthesize 2,3-diaroyl quinolines 34 in a direct manner (Scheme 19). I_2_/DMSO-controlled reaction conditions allow for the easy functionalization of these quinolines to form pyridazino[4,5-*b*]quinolines 35. This scheme has good potential in synthesis chemistry owing to its metal-free, one-pot, shortened, cyclocondensation in a [3 + 2 + 1] manner of three components, *i.e.*, enaminone 18, aryl methyl ketone 4 and aryl amine 2, with mild to good yields. All three reactants having EDGs and EWGs as substituents are compatible to achieve the desired products without any significant effect on the yields; however, none of the alkyl reactants were compatible to generate the desired products. For the Kornblum oxidation, Povarov reaction, and intramolecular cyclization to occur, molecular iodine is the key ingredient. These reactions are dependent on the presence of I_2_, given that they cannot proceed without it (Scheme 20).

**Scheme 21:**
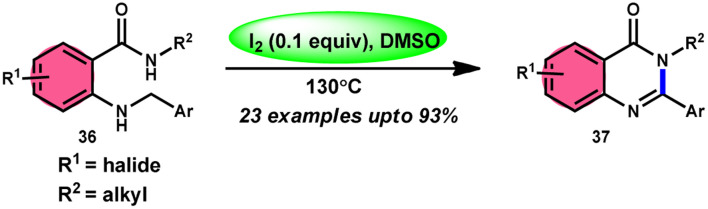
Intramolecular oxidative cross-coupling reaction mediated by I_2_/DMSO.

**Scheme 22:**
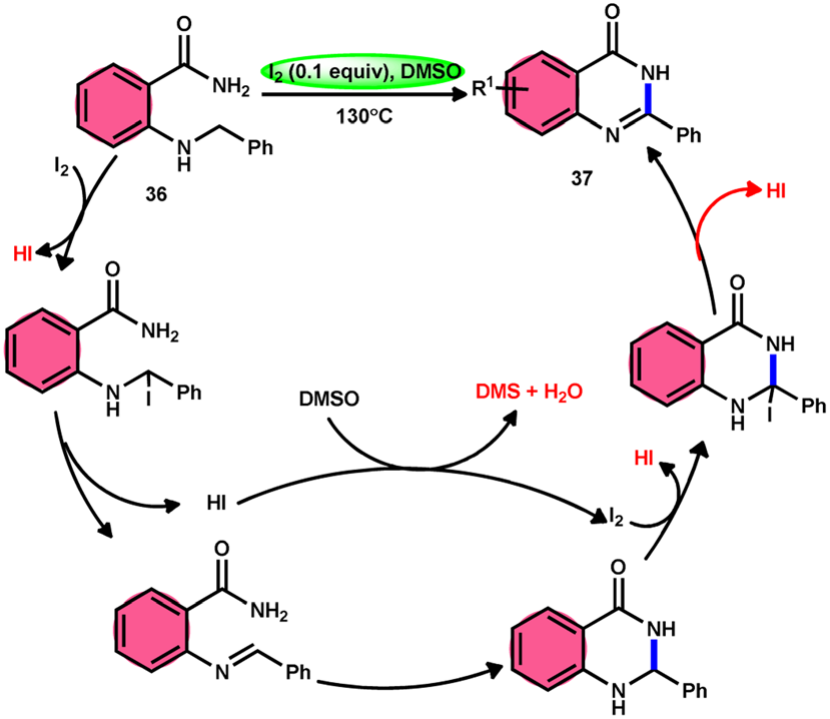
Mechanism of I_2_/DMSO-mediated intramolecular C(sp^3^)–H/N–H oxidative cross-coupling reaction.

Zhou and colleagues^69^ presented an intramolecular C(sp^3^)–H/N–H oxidative cross-coupling process for the synthesis of quinazolinones 37 (Scheme 21). This approach includes the use of I_2_/DMSO to facilitate the intramolecular oxidative cross-coupling reaction. The reaction transforms 2-(benzylamino) benzamides 36 into arylquinazolinones 37 by forming C=N bonds. This approach exhibits remarkable properties such as excellent functional group tolerance, absence of metal catalysts, straightforward procedure, practicality, and high product yields (up to 93%). According to the proposed mechanism, initially, 36 reacts with iodine, resulting in the formation of an iodine intermediate. Furthermore, the intermediate catalyzes the elimination and liberation of HI inside the molecule, resulting in the formation of imine. This imine undergoes intramolecular addition, resulting in the formation of a cyclized intermediate. This cyclized intermediate undergoes a reaction with iodine to produce another intermediate, which is iodized. The intermediate catalyzes the elimination and liberation of HI intramolecularly, resulting in the formation of the final product 37 (Scheme 22). The crucial aspect of this reaction is that HI can undergo oxidation and be restored to iodine by the action of dimethyl sulfoxide.

**Scheme 23:**
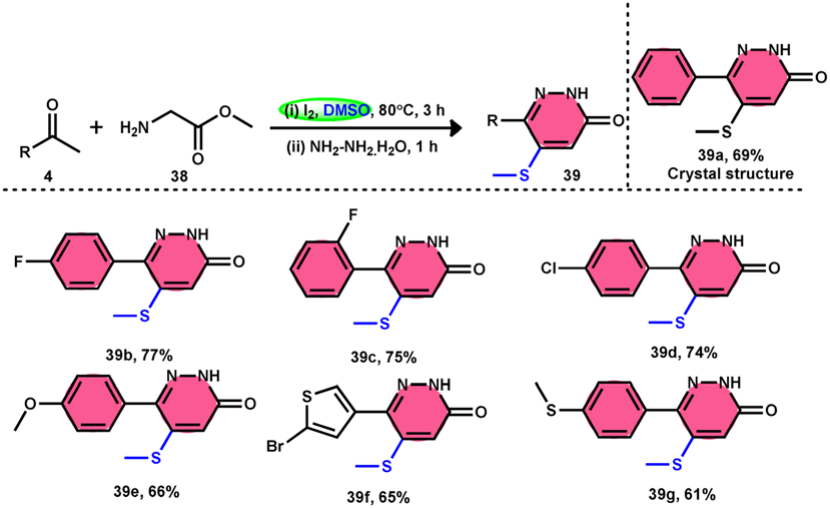
Synthesis of 5-(methylthio)pyridazin-3(2*H*)-ones promoted by I_2_–DMSO.

**Scheme 24:**
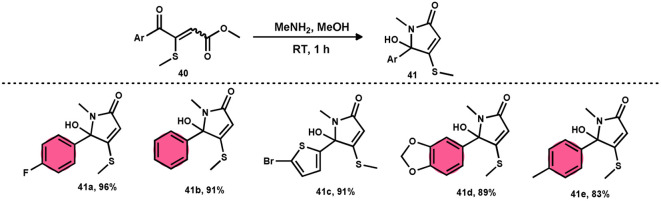
Applications of 3-(methylthio)-4-oxo-enoates 40 and representative examples.

Bhat *et al.*^70^ developed a new approach for synthesizing 5-(methylthio)pyridazinone derivatives 39 (Scheme 23). This method involves the use of iodine to stimulate the deaminative coupling of glycine esters 38 with methyl ketones 4 and hydrazine hydrate in DMSO, resulting in a one-step process. In the absence of hydrazine, these modifications facilitated the production of various 3-methylthio-4-oxo-enoates 40 with high efficiency. DMSO played several roles, including an oxidant, methylthiolating reagent, and solvent. Pyridazin-3(2*H*)-one is a nitrogen-containing aromatic ring structure that is present in several natural products, medicines, and functional materials. The reaction is straightforward and has a wide range of substrates that can be employed, as well as being able to tolerate various functional groups. Furthermore, this technique can be readily modified to serve as an *in situ* generator of various alkyl-3-(methylthio)-4-oxo-enoates 40. The usefulness of 40 has been confirmed by synthesizing several new 5-hydroxy-1-methyl-4-(methylthio)-5-phenyl-1,5-dihydro-2H-pyrrol-2-ones 41 (Scheme 24).

## 5. Six-membered ring formation with three-heteroatoms

**Scheme 25:**
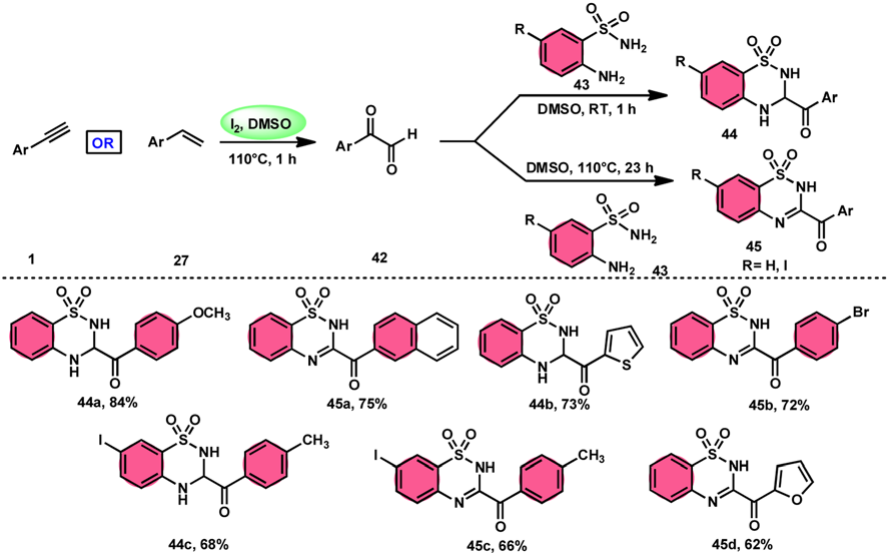
Synthesis of benzothiadiazine-dioxides 44 and 45 and representative examples.

**Scheme 26:**
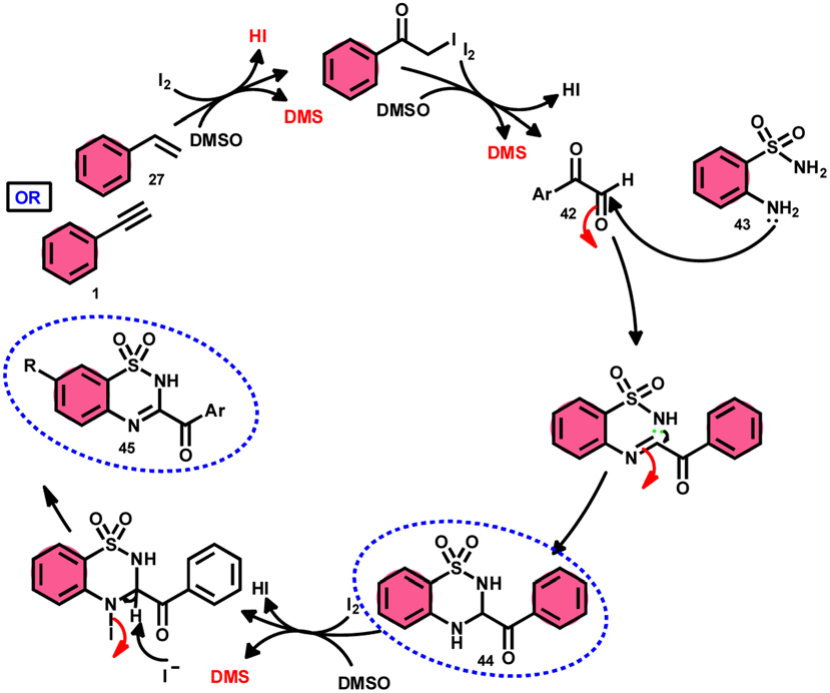
Mechanism for the synthesis of benzothiadiazine-dioxides 44 and 45.

A new, metal-free, efficient, and scalable approach was devised for the production of 3-acyl-3,4-dihydro-2*H*-1,2,4-benzothiadiazine-1,1-dioxides 44 and 3-acyl-2*H*-1,2,4-benzothiadiazine-1,1-dioxide 45 (Scheme 25). In their study, Chaskar *et al.*^71^ employed affordable ethynyl arenes 1 and ethenyl arenes 27, which are readily accessible, as starting materials. These compounds consist of sp^2^ and sp^3^ carbon–hydrogen (C–H) bonds, respectively. By using Kornblum oxidation, both types of bonds were functionalized to produce phenylglyoxal 42 with the help of I_2_/DMSO. In the second reaction, 2-aminobenzenesulfonamides 43 are added to phenylglyoxal 42, which results in the desired product through condensation, followed by intramolecular cyclization and aromatization. However, on adding 2-amino-*N-*phenylbenzenesulfonamide instead of 2-aminobenzenesulfonamides, 43 does not follow the cyclization path, resulting in the formation of oxidative cross-coupling products. DMSO plays a binary role as an oxidant and solvent, whereas molecular iodine is a vital part of Kornblum oxidation, iodination, and aromatization of the product. In summary, the methodology has good value in pharmaceutical and synthetic chemistry owing to its low cost, good substrate scope, and eco-friendly nature (Scheme 26).

## 6. Five-membered ring formation with one-heteroatom

**Scheme 27:**
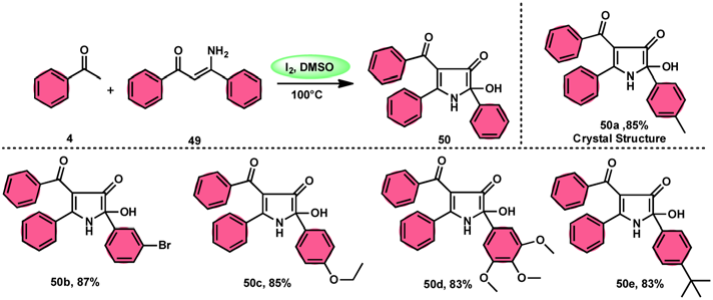
Synthesis of substituted pyrrole 50.

**Scheme 28:**
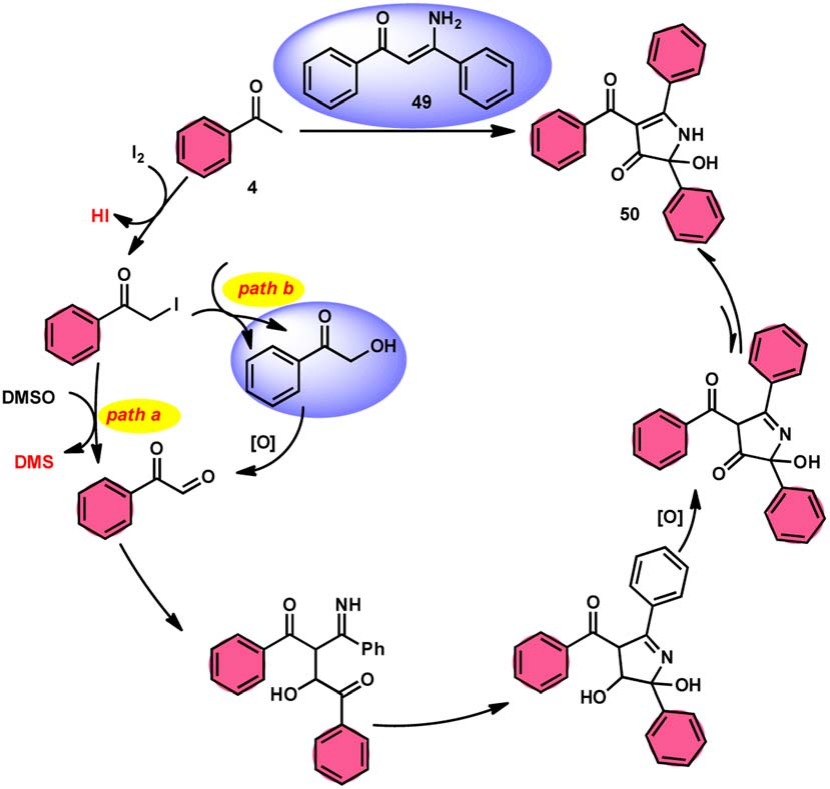
Mechanism for the synthesis of substituted pyrrole 50.

To realize the formation of 2-hydroxy-pyrrol-3(2*H*)-ones 39, Wu *et al.*^72^ developed a new metal-free and I_2_/DMSO-supported cyclization method (Scheme 27). The experimental procedure included the reaction between enaminone 49 and aryl methyl ketones 4. This approach allows for the production of 2-hydroxy-pyrrol-3(2*H*)-one rings 50 with diverse structural properties, resulting in high yields. Furthermore, it successfully produced quaternary alcohol. This depicts the use of group-assisted purification (GAP) chemistry, whereby the purification of the product is achieved by a simple washing process with CH_2_Cl_2_ solvent. This method avoids the need for conventional procedures such as chromatography and recrystallization. The reaction had moderate and straightforward working conditions, was very efficient, and demonstrated strong functional group compatibility. In contrast to electron-withdrawing groups, which demonstrate compatibility only with the *para*- or *meta* positions, and electron-donating groups attached to the aromatic ring of aryl methyl ketones 4 can effectively produce the desired compounds, regardless of their position in the *ortho*, *meta*, or *para*-positions. The suitable 2-hydroxy-pyrrol-3(2*H*)-ones 50 were generated with exceptional yield using halogenated aryl methyl ketones, as can be seen in Scheme 28.

**Scheme 29:**
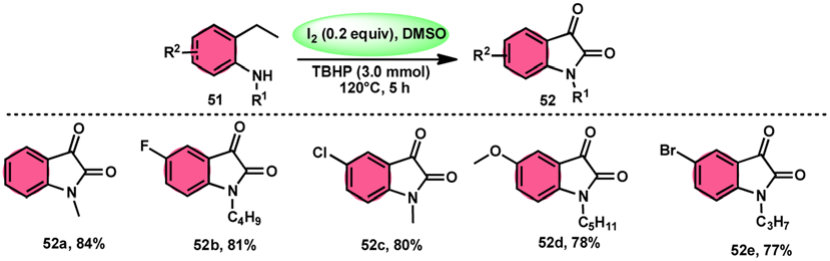
Synthesis of isatin 52 catalyzed by I_2_–DMSO and representative examples.

Nayaki Salvanna and coworkers^73^ established a novel, metal-free, and efficient methodology to prepare isatins^74^52 from 2-ethylanilines or 2-vinylanilines 51 through oxidative intramolecular cyclization. The I_2_/DMSO combination without TBHP (tertiary butyl hydroperoxide), which works as an oxidant, was unsuccessful in generating the selected product. This approach successfully functionalized both sp^2^ and sp^3^ C–H bonds. In addition, its broad substrate scope and strong functional group tolerance make it very useful. According to the researchers, TBHP initiates the reaction by generating secondary alcohol from ethylanilines 51 in a radical manner and the alcohol is easily oxidized to ketone, producing 2-aminoacetophenone by molecular iodine. 2-Aminoacetophenone produces 2-aminophenylglyoxal *via* Kornblum oxidation, which undergoes intramolecular cyclization, forming a C–N bond and a 2-hydroxyquinoline-3-one intermediate, which is oxidized by I_2_/DMSO to afford isatin 52 (Scheme 29).

## 7. Five-membered ring formation with two-heteroatoms

**Scheme 30:**
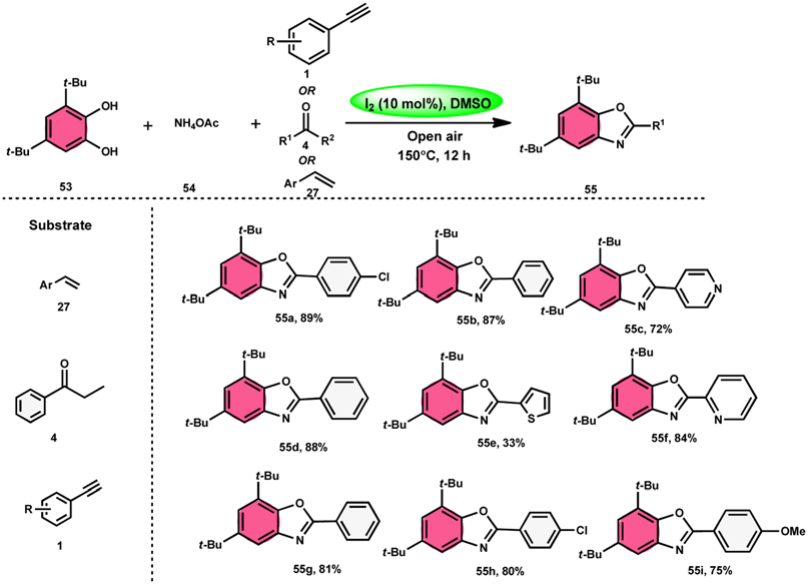
Synthesis of benzoxazoles 55 from phenylacetylenes 1, styrenes 27 and different ketones 4.

Panahi, Sharghi, and colleagues^75^ proposed a method to produce benzoxazoles 55 without the use of metals. This involves combining ammonium acetate 54 with catechols 53 and alkenes/alkynes/ketones. This methodology is simplified by using I_2_/DMSO, which is a one-pot method, suitable for large-scale procedures, and operationally efficient. When neither molecular iodine nor DMSO solvent was used, no product was detected. Besides the dimethyl sulfoxide (DMSO) solvent, only a 30% product yield was reported when using *N*,*N*-dimethylformamide (DMF). Furthermore, there was a significant decline in the yield at lower temperatures. At a temperature of 100 °C, a minute quantity of product was identified. To demonstrate the effectiveness of this process, a large-scale synthesis was conducted, resulting in the isolation of 70% of the product (Scheme 30).

**Scheme 31:**
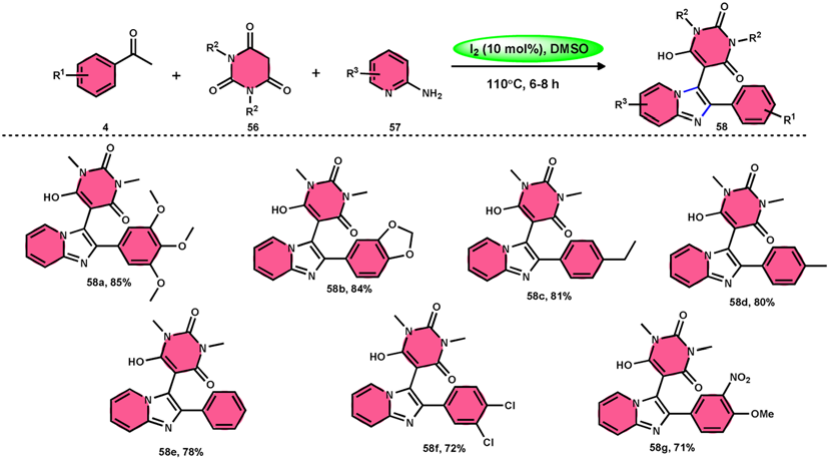
I_2_–DMSO-catalyzed synthesis of highly fluorescent pyrimidine-linked imidazopyridines 58.

**Scheme 32:**
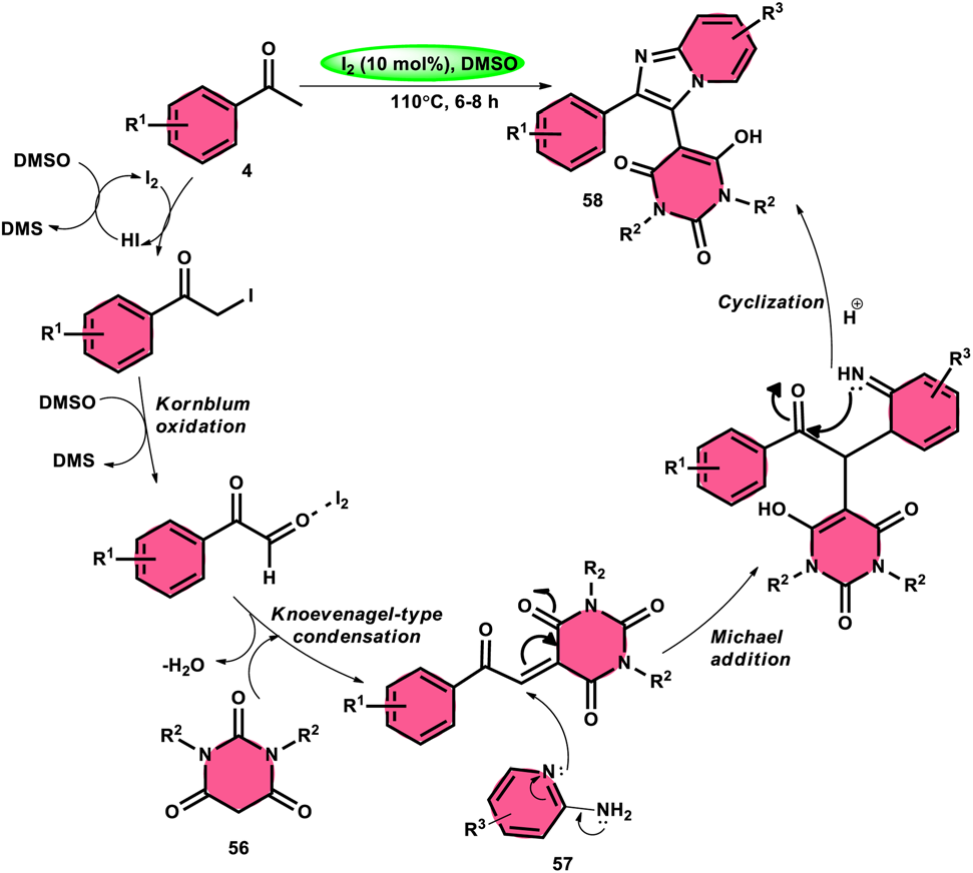
Mechanism for the synthesis of iodine-catalyzed pyrimidine-linked imidazopyridines 58.

Choudhury *et al.*^76^ identified a method for synthesizing pyrimidine-linked imidazopyridine 58 (Scheme 31). This method involves the use of aryl methyl ketones 4, 2-aminopyridines 57, and barbituric acids 56 with the addition of a small quantity of molecular iodine as a catalyst. The synthesis takes place in a DMSO medium. C–H oxidation and the subsequent production of one C–C and two C–N bonds are the steps in this metal-free one-pot method. The majority of the synthesized compounds had a significant fluorescence quantum yield, ranging from very good to exceptional. The reaction process involves the first reaction of acetophenone derivative 4 with iodine, resulting in the formation of an intermediate and the byproduct HI. The presence of DMSO allows the regeneration of I_2_ in the following cycle. Subsequently, in the presence of DMSO, the intermediate undergoes a transformation into the equivalent phenyl glyoxal. This phenyl glyoxal then reacts with 56 by Knoevenagel condensation to get a compound. The nucleophilic addition of 56 to 57 results in the formation of an intermediate. This intermediate undergoes cyclization to form a compound. Subsequent elimination of water (H_2_O) from the compound leads to the formation of the intended bioactive product 58 (Scheme 32).

**Scheme 33:**
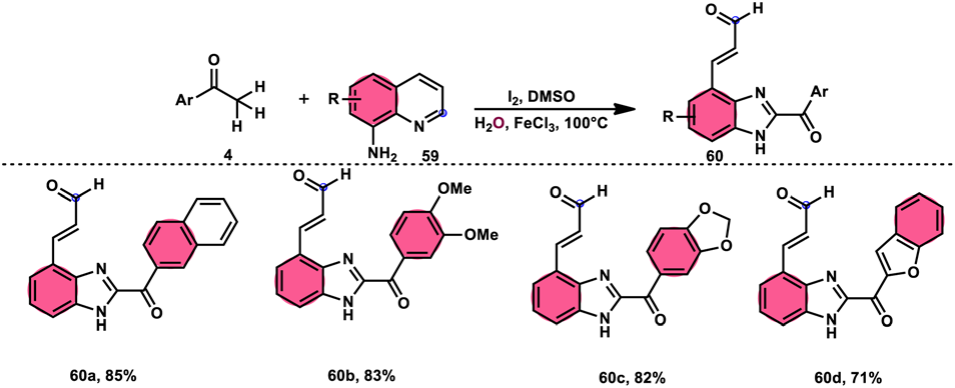
Synthesis of benzoimidazolylacrylaldehyde 60 and selected examples.

**Scheme 34:**
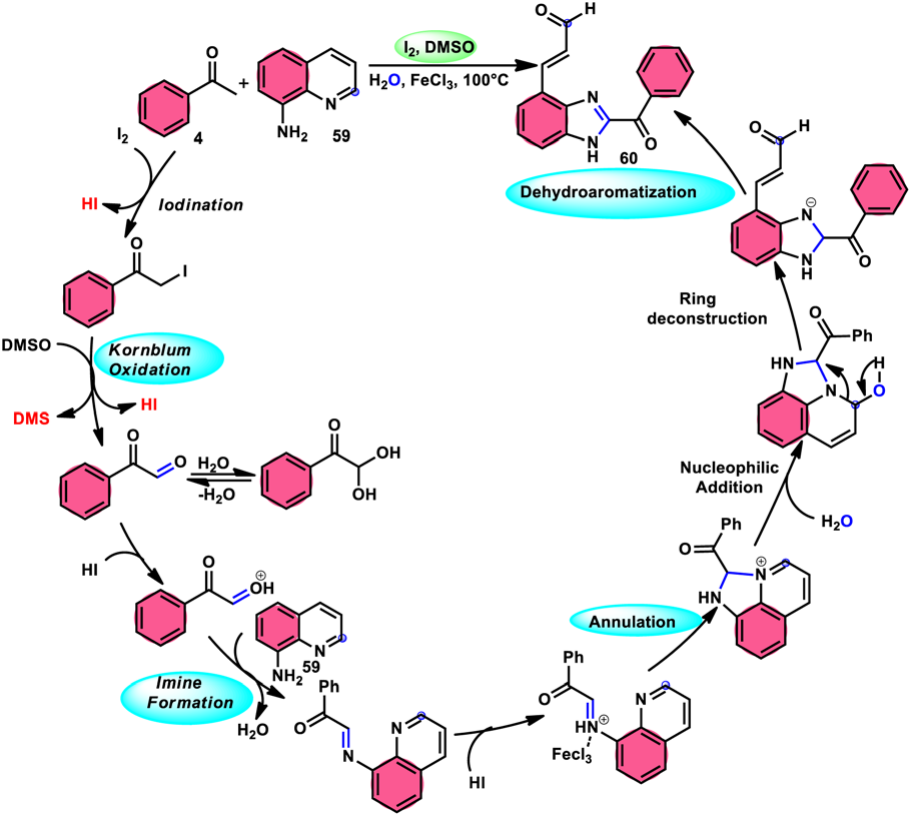
Mechanism for the synthesis of benzoimidazolylacrylaldehydes 60.

Wu *et al.*^77^ effectively demonstrated a significant cascade reaction involving aryl methyl ketones 4 and 8-aminoquinolines 59, using I_2_/DMSO as the catalyst (Scheme 33). The synthesis of (*E*)-3-(2-acyl-1*H*-benzo[*d*]imidazole-4-yl)acrylaldehydes 60 was achieved using a combination of annulation and ring deconstruction methodologies. This procedure was carried out for 8 h at 100 °C. The use of this new methodology resulted in enhanced reactivity of 8-aminoquinolines 59, therefore providing a favourable framework for the activation of unreactive N-heteroaromatic compounds through ring-opening procedures. The reaction in the absence of I_2_ signified the crucial functionality of molecular iodine as a prominent chemical mediator. Aryl methyl ketones 4 have a tendency to easily engage in interactions with both electron-efficient and electron-deficient groups, leading to the production of products with yields that vary from moderate to excellent. This study not only revealed the unique reactivity of 8-aminoquinolines 59, but also presented a potential structure for the initiation of unreactive N-heteroaromatic compounds by ring opening (Scheme 34).

**Scheme 35:**
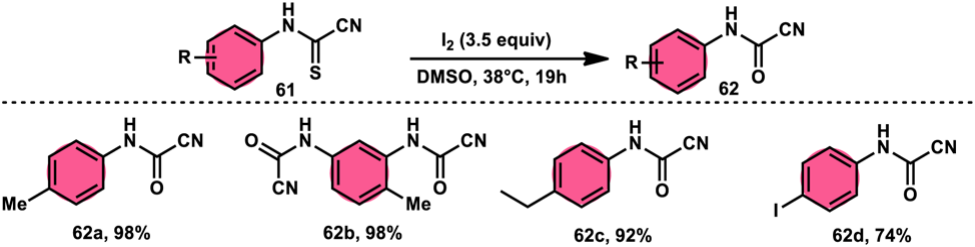
Synthesis of *N*-arylcyanoformamide 62 and representative examples.

**Scheme 36:**
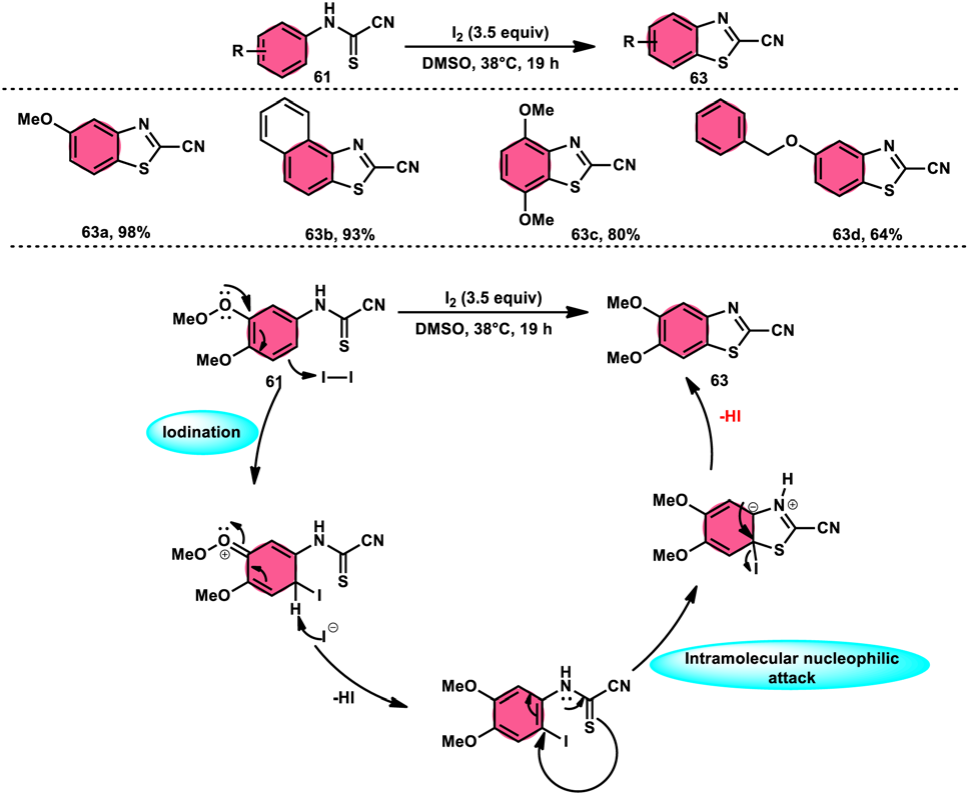
Synthesis and mechanism of 2-cyanobenzothiazoles 63.

Ziad Moussa *et al.*^78^ provided information regarding the viability of using the I_2_–DMSO oxidative system for the production of *N*-arylcyanoformamides 62 at 38 °C using *N*-arylcyanothioformamides 61 (Scheme 35). The synthesis of important intermediates and bioactive compounds frequently involves the use of cyanoformamides as useful building blocks. This synthetic approach employed a diverse array of substrates, operated under gentle conditions, and exhibited exceptional reaction efficiency. Furthermore, it presents a novel and unconventional means to obtain 2-cyanobenzothiazoles 63 (as seen in Scheme 36). These compounds serve as valuable substrates for the identification of distinct luciferin analogues. The reaction exhibited tolerance towards a diverse array of functional groups, including various alkoxides, halides, esters, nitro, thiomethyl, cyano, and trifluoromethyl functionalities, resulting in the formation of a broad spectrum of products. Because there was no apparent improvement in conversion, KI was not an acceptable alternative for iodine. The biological actions of the benzothiazole nucleus are extremely diverse. For example, improve breast cancer diagnosis and therapy, 2-cyanobenzothiazole 63 was recently integrated in gold nanoparticles.^79^

**Scheme 37:**
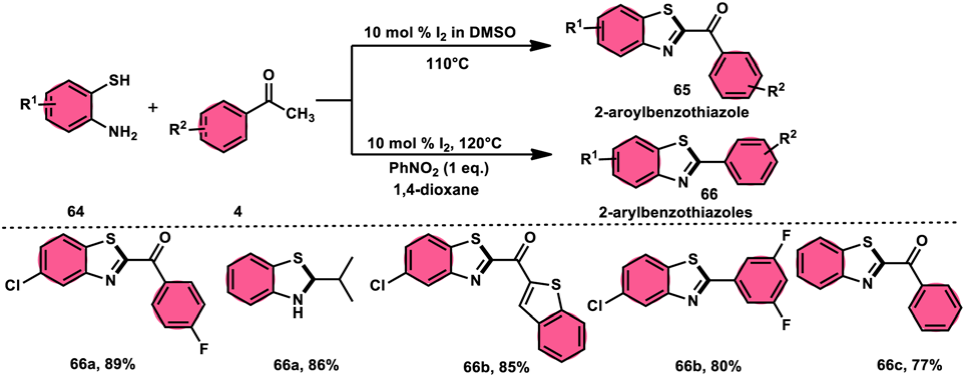
Synthesis of aroylbenzothiazole 65 and arylbenzothiazoles 66 in I_2_–DMSO system.

**Scheme 38:**
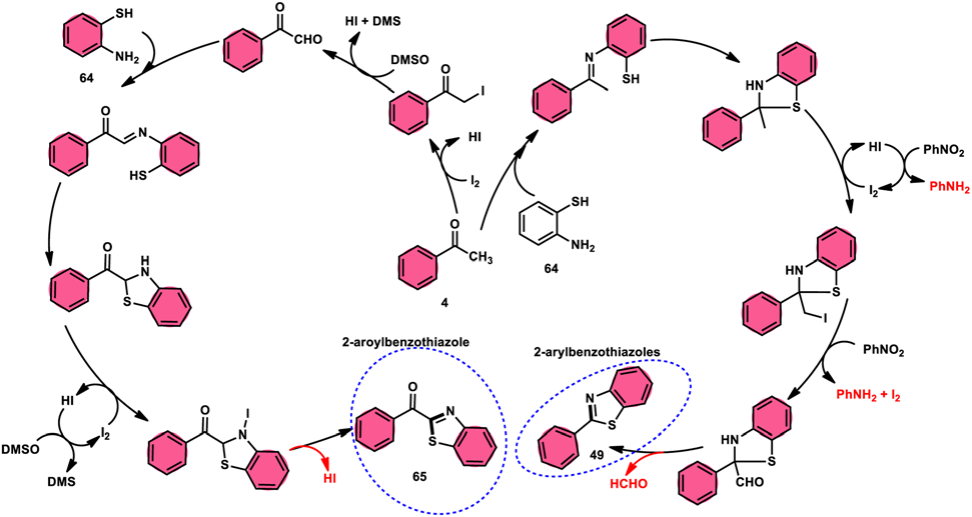
Mechanism for the synthesis of aroylbenzothiazole 65 and arylbenzothiazoles 66 using I_2_–DMSO system.

Ma *et al.*^80^ developed a unique, domino solvent-based selective methodology to prepare 2-aryl benzothiazole 66 and 2-aroylbenzothioazole 65 mediated by I_2_/DMSO or nitrobenzene/1,4-dioxane, respectively, starting with substrates aryl methyl ketone 4 and 2-aminobenzene thiol 64 (Scheme 37). Both EWG and EDG substituents on both substrates were well tolerated and the yields remained unaltered owing to presence of substituents. The generation of different products is due to the oxidation of aryl methyl ketone 4 to phenylglyoxal in the DMSO/I_2_-mediated reaction, where the aldehydic group of the phenyl glyoxal reacts with amino moiety of the substrate, leading to the formation of 2-aroylbenzothiazole 65, whereas in PhNO_2_/dioxane, the ketonic group without any oxidation step directly reacts with the amine moiety of aminobenzene thiol 64 and results in the formation of 2-aryl benzothiazole 66. The formation of the imine intermediate is initiated through the reaction between the aldehydic or ketonic group and the amine of aminobenzene thiol 64. This is followed by an intramolecular cyclization process, facilitated by the thiol group of the latter substrate, resulting in the formation of a C–S bond. In this reaction scheme, PhNO_2_ acts as an oxidant, oxidizing the methyl group of the aryl benzothiazole intermediate to an aldehydic group. Subsequently, the aldehydic group undergoes elimination, producing formaldehyde as a byproduct. Ultimately, this series of reactions leads to the formation of 2-aryl benzothiazole 66 (Scheme 38).

**Scheme 39:**
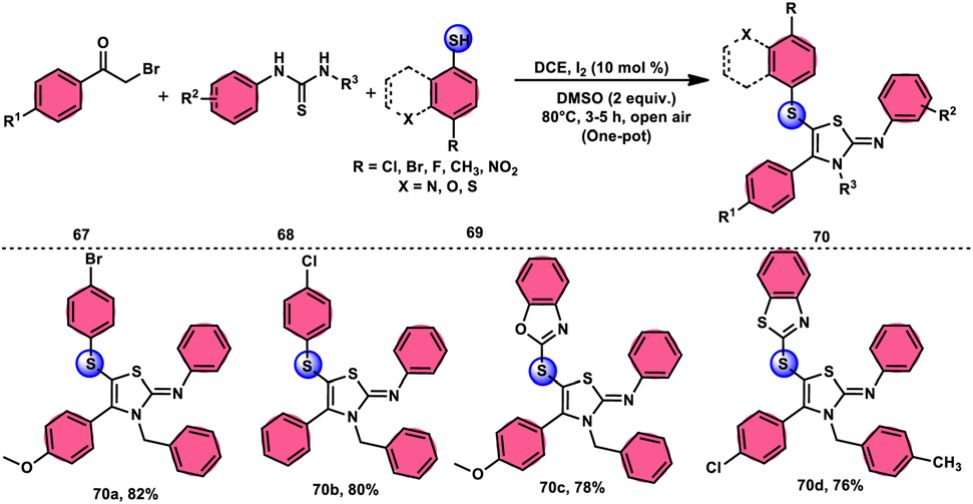
I_2_–DMSO-based preparation of 5-sulfenyl-2-iminothiazolines 70 and representative examples.

Animesh Pramanik and Bodhak^81^ proposed a metal-free, open-air, one-pot method for the regioselective sulfenylation of 2-iminothiazoline 70 at the C-5 position. This approach utilizes the I_2_/DMSO combination as a catalytic oxidant. The reaction takes place in the C_2_H_4_Cl_2_ (DCE) solvent and involves the interaction between phenacyl bromide 67 and thiourea 68. This interaction leads to the formation of the 2-iminothiazoline intermediate. The C–H bond in this intermediate becomes reactive due to the presence of I_2_ as a catalyst. Subsequently, the oxidant DMSO facilitates the regioselective sulfenylation at the C-5 position. This series of reactions ultimately yields the desired product, which is 5-sulfenyl-2-iminothiazoline derivative 70. The applicability of this methodology increases when the chemoselective bridged S-atom of the desired product is oxidized into sulfoxide, and the reaction also proceeds smoothly whether all three substrates have EDGs or EWGs, pyridyls or heterocyclic thiols and gives good yields in all cases, showing its good functional tolerance. Interestingly, 2-iminothiozoline intermediate 70 achieved through the condensation between phenacyl bromide 67 and thiourea 68 does not require I_2_/DMSO; however, the diaryl disulfide intermediate, which forms *in situ* from aromatic thiols 69 and nucleophilic attack on the 2-iminothiozolines intermediate on the S atom of the former intermediate through C–H bond activation leads to the desired product, although I_2_/DMSO is indispensable in both steps (Scheme 39).

**Scheme 40:**
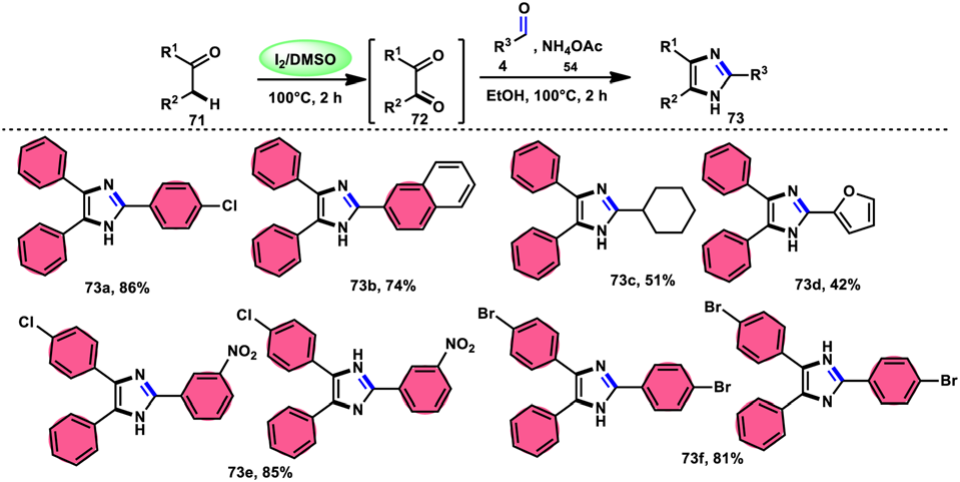
I_2_/DMSO-catalyzed synthesis of trisubstituted-imidazoles 73 and representative examples.

In the study by Jeena and Jayram,^82^ they proposed an enhanced and environmentally sustainable approach for the construction of 2,4,5-trisubstituted imidazole 73 and its bioactive scaffold. This technique eliminates the need for acid/base and transition metal catalysts, resulting in a more time-efficient and cost-effective process. The authors used I_2_/DMSO as a promoter in the oxidative cyclization of the C–H bond, leading to increased yields and reduced environmental impact. The reactants α-methylene ketone 71, aldehydes and NH_4_OAc 54, which is used as a nitrogen source, are readily accessible and inexpensive. Furthermore, the broad substrate scope of this methodology makes its applicable in the synthesis of various derivates of the target product, which can be used as pharmaceutical scaffolds. The use of benzyl alcohol instead of benzyl aldehyde is feasible given that benzyl alcohol and benzyl phenyl ketone are concurrently oxidized in benzene aldehyde and benzil, respectively, leading to the target product in moderate yields through the domino convergent reaction path approach. According to the plausible reaction mechanism, molecular iodine converts methylene ketone 71 into diketones 72*via* the radical path, and both carbonyl reactants further react with the *in situ*-generated NH_3_ from NH_4_OAC 54, affording the imine intermediate. Finally, the condensation between these imine intermediates results in the desired products 73 (Scheme 40).

**Scheme 41:**
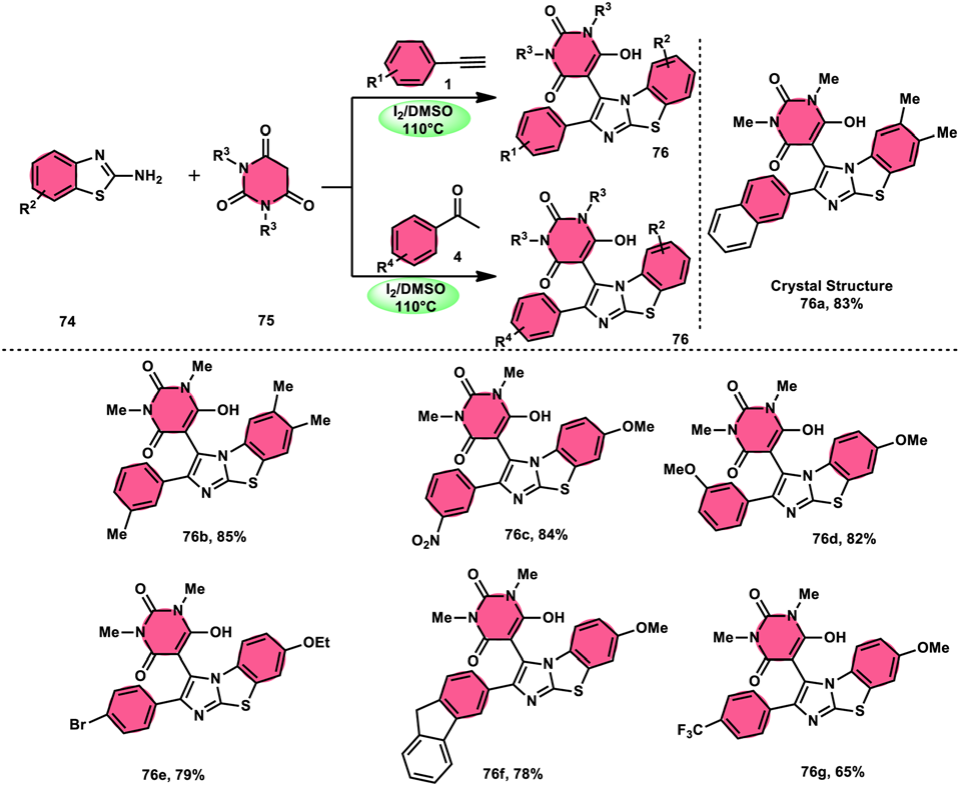
Synthesis of 2-arylbenzo[*d*]imidazo[2,1-*b*]thiazole 76 catalyzed by I_2_/DMSO.

**Scheme 42:**
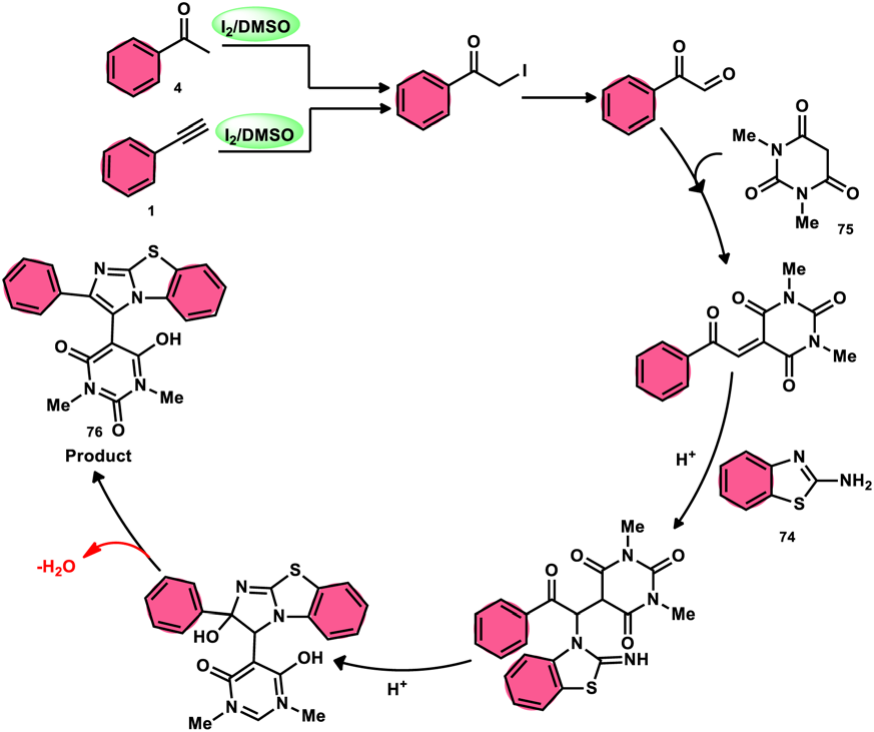
Mechanism for the synthesis of 2-arylbenzo[*d*]imidazo[2,1-*b*]thiazole 76 catalyzed by I_2_/DMSO.

The research group led by Lokman H. Choudhury devised a method, as shown in Scheme 41,^50^ for the synthesis of 2-arylbenzo[*d*]imidazo[2,1-*b*]thiazoles 76 derived from barbituric acid 75. This method utilizes an I_2_/DMSO-based reaction system, which is both effective and environmentally benign. Furthermore, this metal-free, one-pot, three-component reaction (MCR) offers an efficient approach for the synthesis of the desired compounds. The methodology has practical value owing to the fact that its product and its arrays have medicinal applications, its good substrate scope, competence under traditional or microwave heating condition, and utilized reactants, *i.e.*, barbituric acid 75, 2-aminobenzothizole 74 and aryl methyl ketone 4 or aryl acetylene 1. The reaction is easily accessible, inexpensive and its yields are good to excellent. The methodology proceeds *via* Kornblum oxidation, converting aryl methyl ketone 4 into aryl glyoxal, which undergoes Knoevenagel condensation, forming a C–C bond with barbituric acid, aza-Michael addition by 2-aminobenzothiazole and intramolecular cyclization of the intermediate, affording two C–N bonds, which results in the targeted products, and molecular iodine plays an important role in the oxidation and cyclization (Scheme 42).

**Scheme 43:**
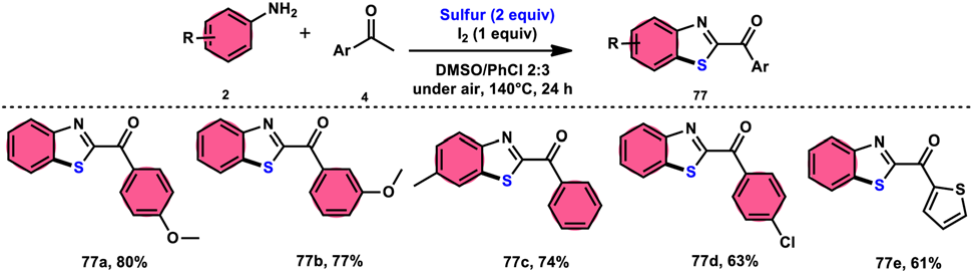
Synthesis of 2-aroylbenzothiazoles 77 catalyzed by I_2_/DMSO.

**Scheme 44:**
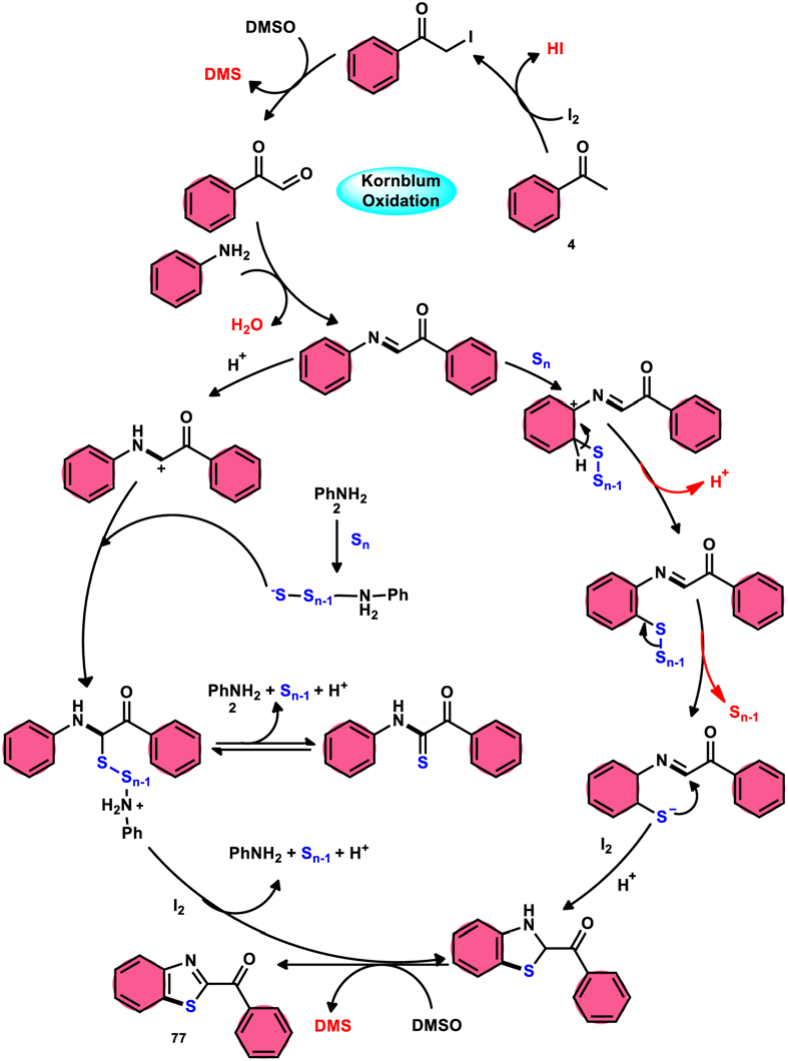
Mechanism for the synthesis of 2-aroylbenzothiazoles 77 catalyzed by I_2_/DMSO.

Phan *et al.*^83^ proposed a new, straightforward, environmentally friendly, transition metal-free, three-component reaction. This reaction utilizes readily available and cost-effective starting materials including elemental sulfur (S_8_), acetophenones 4, and anilines 2 to produce 2-aroylbenzothiazoles 77 (Scheme 43). The molecular iodine-promoted reaction gave the best yields in the solvent combination of DMSO/PhCl in a 2 : 3 ratio, whereas DMSO as an oxidant leads to Kornblum oxidation and aromatization. This strategy exhibits economical value due to the non-toxic nature and commercial availability of the substrates, as well as the favourable functional group tolerance of the substituted acetophenones 4 and anilines 2. According to the proposed reaction mechanism, the combination of I_2_ and DMSO facilitates the Kornblum oxidation, resulting in the conversion of acetophenone 4 to phenylglyoxal. Subsequently, the condensation of an amine ketone forms an imine intermediate. The imine intermediate then undergoes electrophilic attack by S_8_ at the *ortho* position of benzene, followed by intramolecular cyclization to yield the desired product (Scheme 44).

**Scheme 45:**
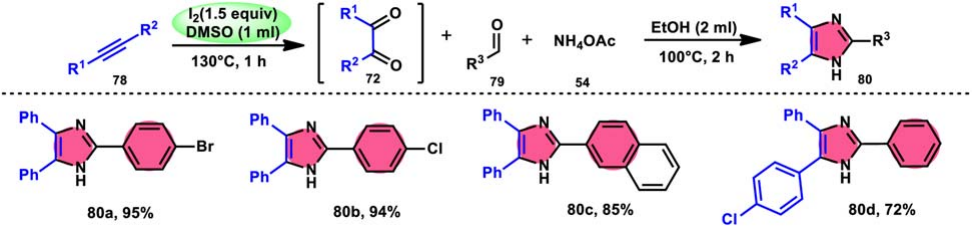
I_2_–DMSO-catalyzed oxidation of internal alkynes 78 and selected examples.

**Scheme 46:**
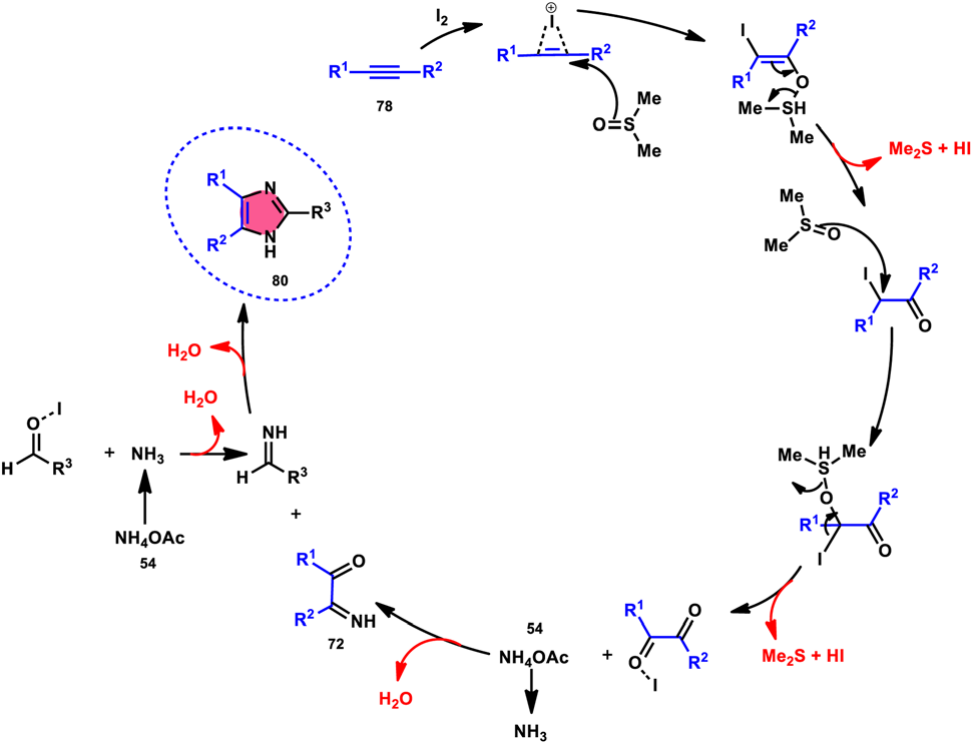
Mechanism for the I_2_–DMSO-catalyzed of oxidation of internal alkynes 78.

Vineet Jeena and Shivani Naidoo^84^ developed a new, practical, economical, metal-free, acid-free, and environmental-benign I_2_/DMSO-mediated approach to prepare trisubstituted imidazoles 80 using the cheap reactants internal alkyne 78, aldehyde 79, and ammonium acetate 54 (Scheme 45). The one-pot oxidative cyclization procedure is concluded in two steps, where in the first step, alkynes 78 are transformed into α-diketone, which further reacts with aldehyde and NH_4_OAc 54*via* cyclic condensation, affording the desired product 80. Benzaldehydes substituted with various EDGs and EWGs are compatible to achieve the target products; however, aliphatic aldehydes result in unsatisfactory yields. The utilization of molecular iodine as a catalyst and DMSO as an oxidant plays significant roles in the described transformation (Scheme 46).

**Scheme 47:**
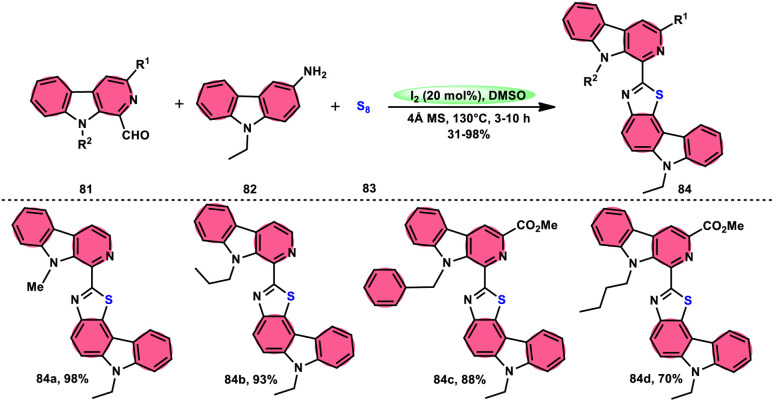
Synthesis of β-carboline C-1-tethered thiazolo[4,5-*c*]carbazole 84 and selected examples.

**Scheme 48:**
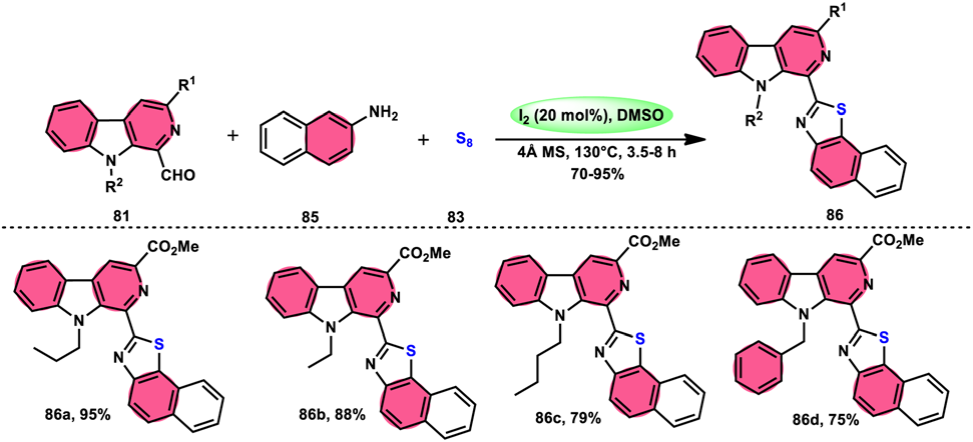
Synthesis of β-carboline C-1-substituted naphtho[2,1-*d*]thiazole 86 and selected examples.

**Scheme 49:**
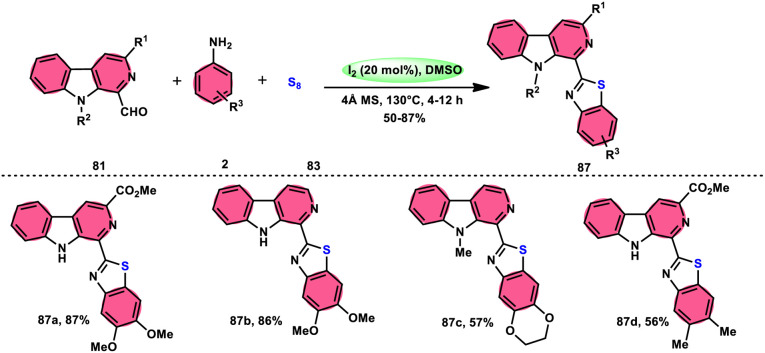
Synthesis of β-carboline C-1-substituted benzothiazole 87 and selected examples.

Singh *et al.*^85^ developed an innovative and effective method, free of metal catalysts, for synthesizing highly fluorescent compounds. This method involves the preparation of β-carboline C-1(3) linked thiazolo[4,5-*c*]carbozoles 84 (Scheme 47), naphtho[2,1-*d*]thioazoles 86 (Scheme 48), and benzothiazole 87 (Scheme 49) using I_2_/KI as a mediator in DMSO solvent. All three variant products were highly fluorescent, and also prepared on a gram scale with outstanding yield of above 90%, making this technique industrially applicable. Both acetal and aldehyde derivatives of Kumujian C (1-formyl-9*H*-β-carbolines) 81, which serve as the base model, exhibit high efficiency in their reaction with elemental sulfur (S_8_) 83, as well as 3-aminocarbazole 82, anilines 2, 2-aminopyridine, and naphthylamine 85. These reactions yield the desired products derived from the corresponding aryl amine derivatives. This methodology has good functional group tolerance on β-carboline (Kumujian) 81 and aniline 2; however, aniline having EWG groups failed to generate the desired products. This strategy involves the formation of two C–S bonds and one C–N bond in a single step through the utilization of an imine intermediate. This reaction is considered to be highly atom economic. Additionally, both potassium iodide (KI) and iodine (I_2_) exhibit equal efficiency in catalyzing these reactions. The research team also deeply investigated the luminous nature of derivates of all three products. All the derivates had good fluorescence with a fluorescence quantum (*Φ*_F_) yield of up to 92%, although thiazolo[4,5-*c*]carbazole 84 had the highest fluorescence properties, which can be used in medical science.

## 8. Five-membered ring formation with three-heteroatoms

**Scheme 50:**
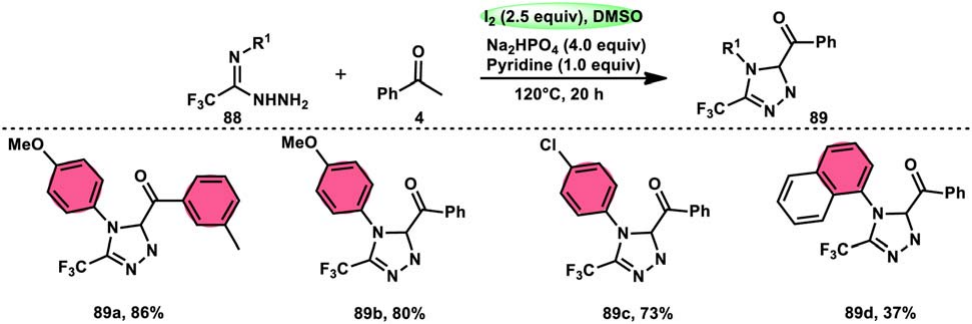
Synthesis of substituted triazoles 74 and selected examples.

**Scheme 51:**
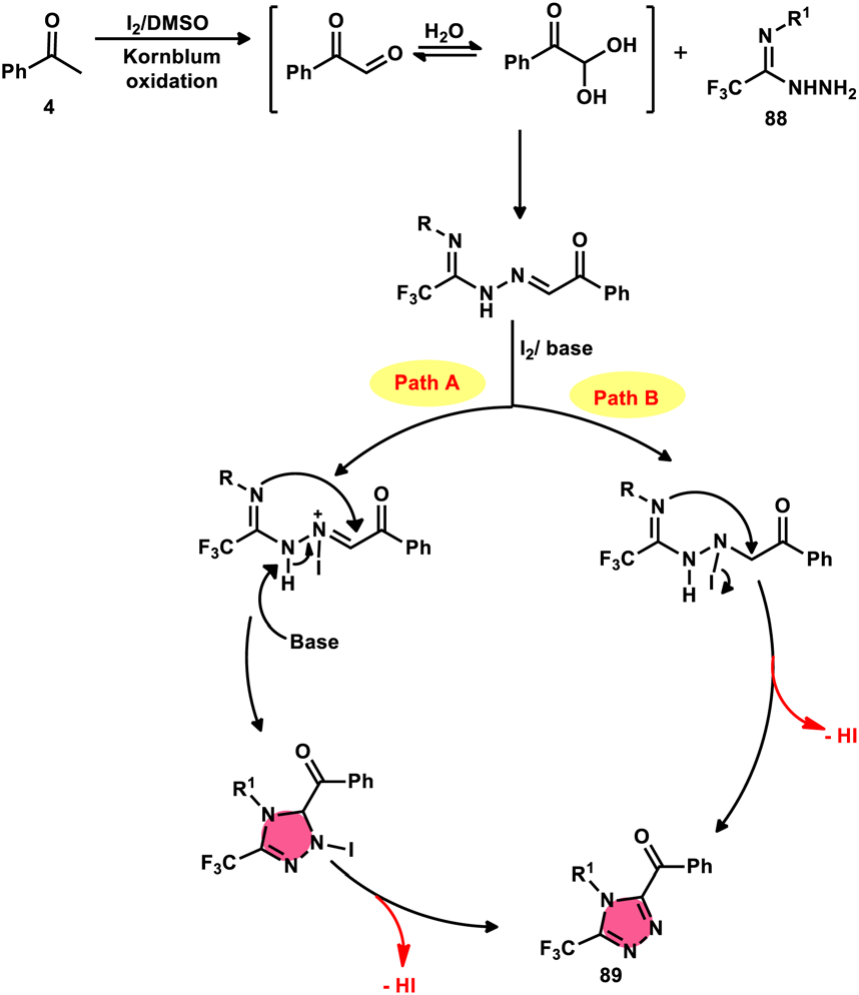
Mechanism for the synthesis of substituted triazoles 89.

The formation of 5-trifluoromethyl-1,2,4-triazoles 89 was achieved by Zhengkai Chen, Xiao-Feng Wu, and colleagues using a metal-free methodology.^86^ This method involves the I_2_-mediated [4 + 1] annulation of readily accessible trifluoroacetimidohydrazides 88 and methyl ketones 4, as illustrated in Scheme 50. The procedure involves iodination/Kornblum oxidation, intermolecular dehydration condensation, and an intramolecular cyclization–aromatization sequence facilitated by iodine. The utilization of the synthesis method presents a viable alternative for obtaining functionalized 1,2,4-triazole derivatives^87^ possessing therapeutic features. The results indicate that the electron factors and steric hindrance of the trifluoroacetimidohydrazides have a limited effect on the outcome of the reaction, as evidenced by the observed comparable efficiency (Scheme 51).

**Scheme 52:**
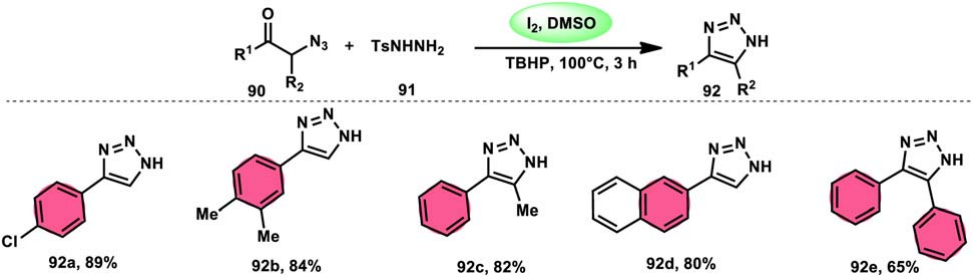
Synthesis of triazoles 92 and selected examples.

**Scheme 53:**
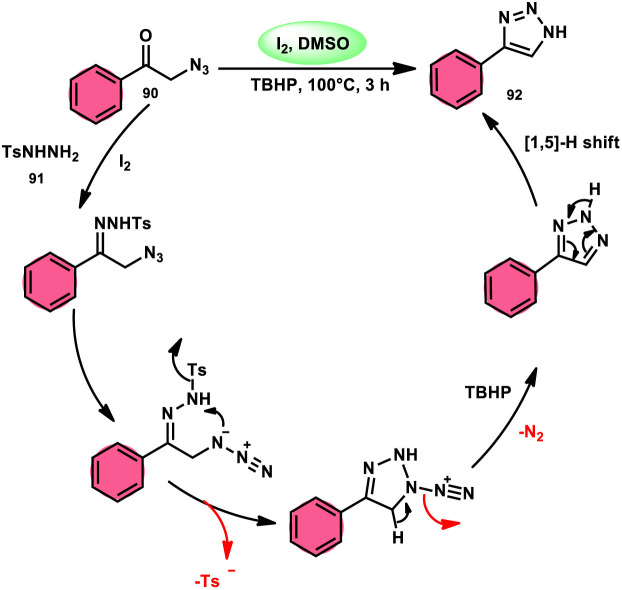
Mechanism involving the synthesis of triazoles 92.

N*H*-1,2,3-Triazoles 92, together with their derivatives, represent a crucial class of heterocyclic compounds that possess diverse and significant pharmacological and biological properties. These properties include the inhibition of HER2, hMetAP2, IDO, VIM-2, as well as anticancer activity.^88–91^ Hence, by employing readily available α-azido acetophenones 90 and *p*-toluene sulfonyl hydrazide 91 in the presence of DMSO solvent, Wen-Ming Shu *et al.* successfully devised a condensation/cyclization methodology mediated by molecular iodine for the production of 4-aryl-N*H*-1,2,3-triazoles 92 (Scheme 52).^92^ Under ideal circumstances, this reaction presents a method that does not require the use of metals to sequentially develop C–N and N–N bonds. The compounds that correspond to α-azido ketones and possess either an electron-donating or withdrawing group can be synthesized in high yields. Substrates with halo-substituted groups (4-F, 3,4-2Cl, 3-Cl, 4-Cl, 3-Br, and 4-Br) also exhibited a favourable performance, resulting in the production of the desired products in significant quantities. The utilization of cyclopropyl α-azido ketone in the annulation reaction was prohibited, resulting in the failure to attain the intended outcome (Scheme 53).

**Scheme 54:**
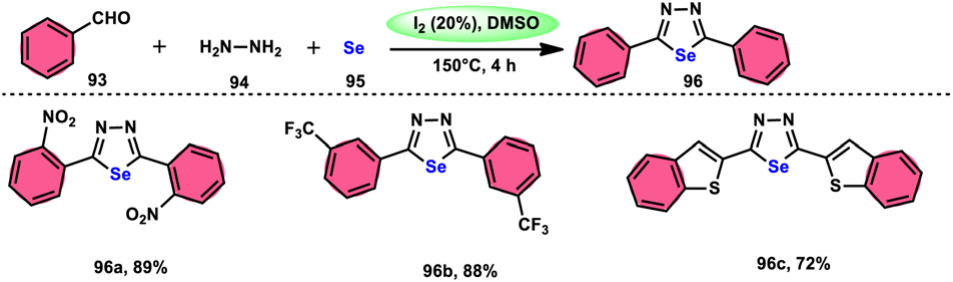
Synthesis of 1,3,4-selenadiazoles 96 and selected examples.

**Scheme 55:**
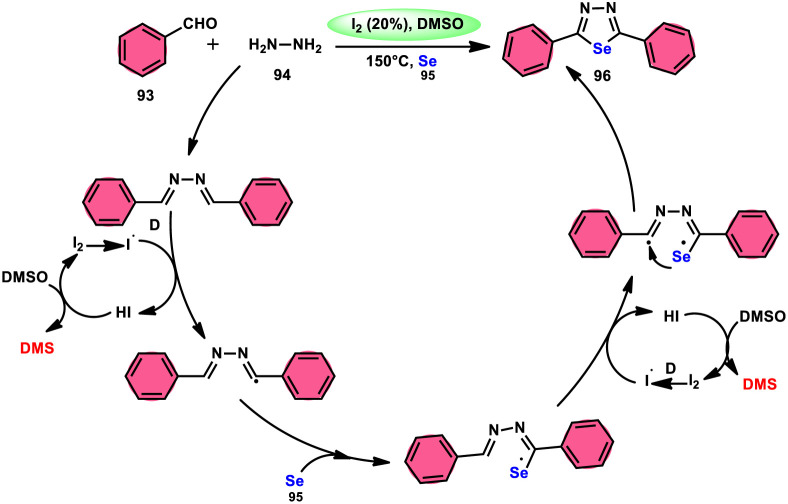
Mechanism involving the synthesis of 1,3,4-selenadiazoles 96.

The synthesis of 1,3,4-selenadiazoles 96 was explained by Bavanthula and colleagues in 2021 utilizing a three-component process involving arylaldehydes 93, hydrazine 94, and elemental selenium 95 in the presence of the I_2_/DMSO system (Scheme 54).^93^ This methodology demonstrates the ability to produce the desired products in moderate to favorable yields. It is characterized by its operational simplicity and effectiveness across a range of functional groups. The reaction tolerating a radical operation is predicted by the postulated mechanism. 1,3,4-Selenadiazoles 96 have demonstrated a wide range of biological actions, ranging from pesticides, fungicides, analgesics, anticancer, anticonvulsants, and anti-inflammatory medications.^94–96^ At 150 °C, the reaction was finished in 4 h. In the control experiment, dibutylhydroxytoluene (BHT) and (tetramethylpiperidin-1-yl)oxidanyl were used as radical inhibitors to stop the reaction. The advantages of the current approach are its basic operation and avoidance of metal. Under the optimal reaction conditions, benzaldehyde with substituents at the *ortho* (*o*), *meta* (*m*), and *para* (*p*) positions of the aromatic ring that are neutral, electron-donating, or electron-withdrawing exhibited efficient reactivity, resulting in the formation of the desired product in significant yields (Scheme 55).

**Scheme 56:**
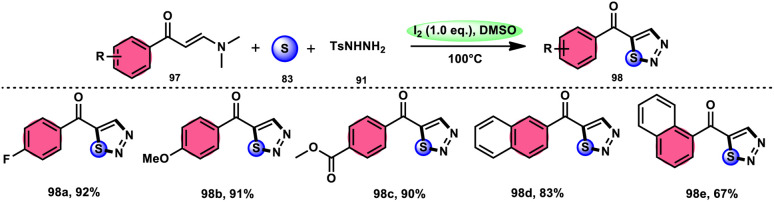
I_2_/DMSO-based synthesis of thiadiazols 98 and selected examples.

**Scheme 57:**
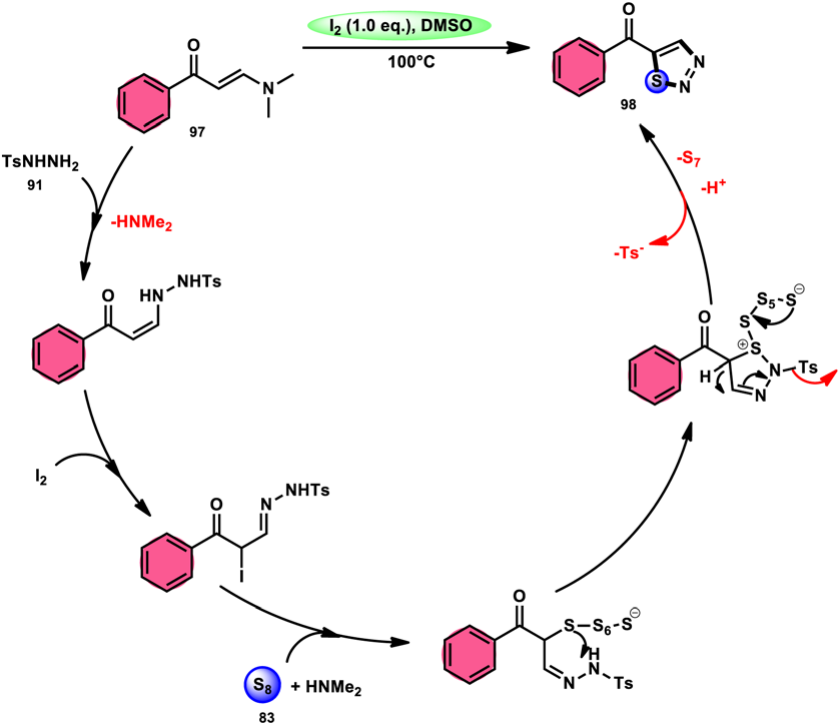
Mechanism for the synthesis of thiadiazols 98 catalyzed by I_2_/DMSO.

In the study by Zhou *et al.*,^97^ they developed a highly efficient method involving three components, without the use of transition metals, to synthesize 5-acyl-1,2,3-thiozole 98. This protocol involves the reaction of enaminones 97, tosylhydrazine (*p*-toluenesulfonyl hydrazide) 91, and elemental sulfur 83, promoted by I_2_/DMSO (as shown in Scheme 56). This strategy facilitates the formation of three significant C–S, C–S, and S–N bonds in a direct manner, resulting in yields ranging from moderate to outstanding. Additionally, the utilization of affordable and easily accessible reactants enhances the practicality of this methodology.^98,99^ The functional group tolerance on aromatic enaminones is very good, whereas alkyl enaminones are incompatible to achieve the targeted product (Scheme 57).

**Scheme 58:**
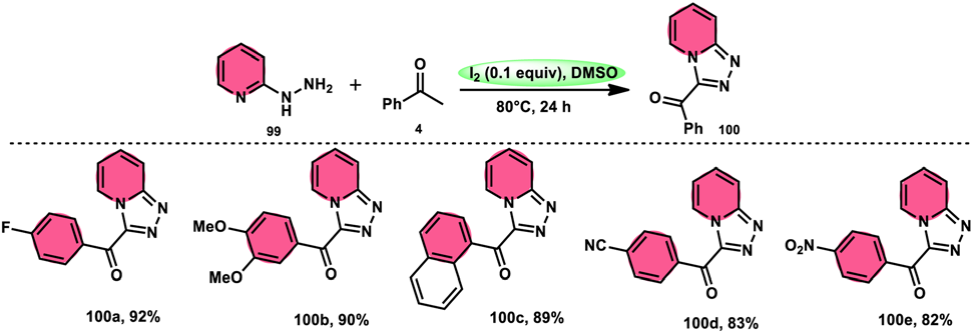
Metal-free I_2_–DMSO-based reaction of 2-hydrazinylpyridines 99 and selected examples.

**Scheme 59:**
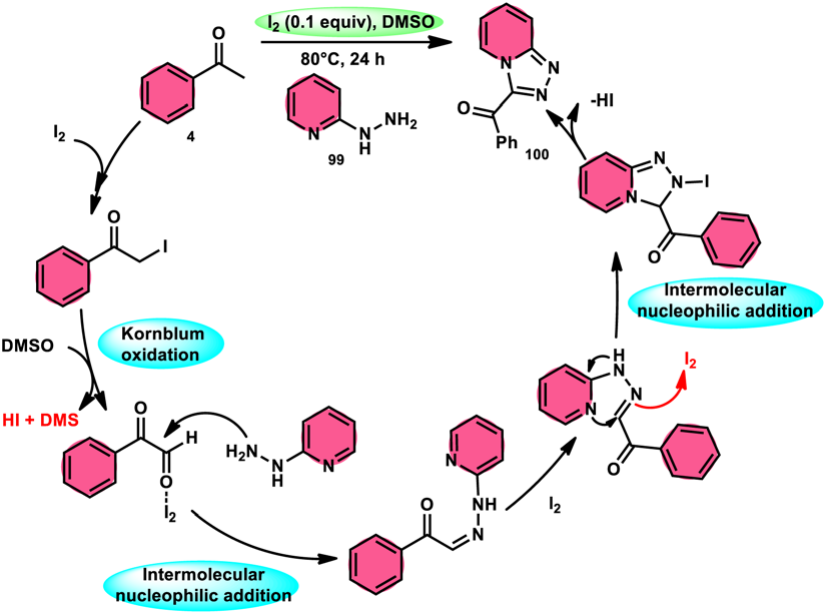
Mechanism for reaction of 2-hydrazinylpyridines 99.

B. V. Subba Reddy and fellows^100^ developed an environmentally friendly, one-pot, metal-free, conducive and highly effective protocol to prepare the bioactive scaffold 3-aryl[1,2,4]triazolo[4,3-*a*]pyridines 100 promoted by I_2_ and DMSO (Scheme 58). This methodology has a broad scope of reactants including acetophenone 4, phenylacetylene, ethyl benzoylacetate, styrene and alcohol with 2-hydrazinopyridine 99; however, styrene and alcohol require 2 equiv. of IBX (2-iodo benzoic acid) besides the general conditions, where IBX converts these reactants to phenylacyl iodide. Further, this strategy is scalable and has a high functional group tolerance on all the reactants and its yields are good to excellent. Molecular iodine plays pivotal role in the oxidative cyclization *via* Kornblum oxidation, producing phenylglyoxal, working as a Lewis acid to induce the intramolecular nucleophilic addition of 2-hydrazinopyridine and intramolecular cycloaddition, which results in the target product (Scheme 59).

**Scheme 60:**
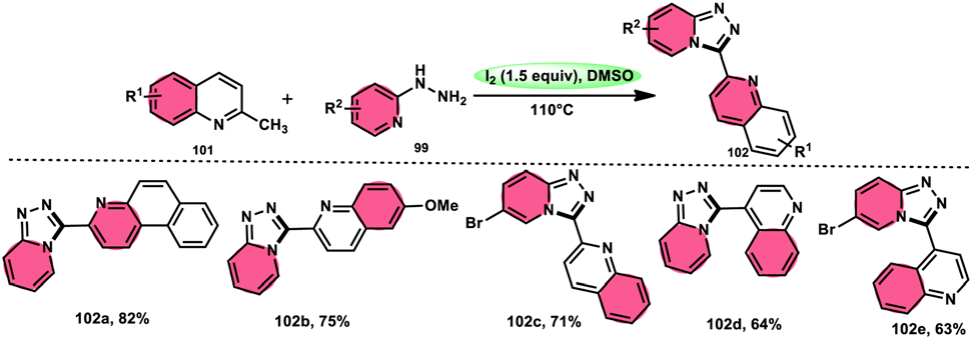
Synthesis of triazolo-pyridines 102 and representative examples.

**Scheme 61:**
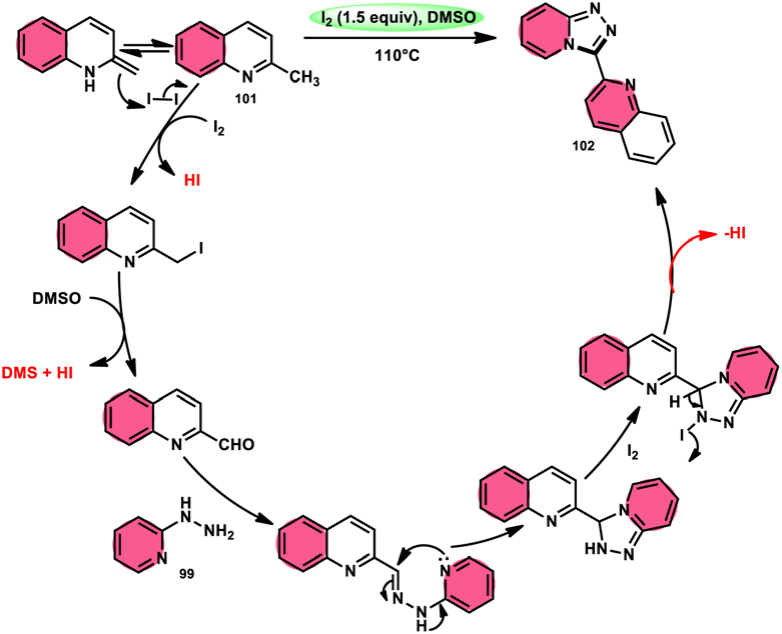
Mechanism for the synthesis of triazolo-pyridines catalyzed by I_2_/DMSO.

Wu *et al.*^101^ developed a new reaction method that does not involve the use of metals or acid/base catalysts. This one-pot, environmentally friendly approach enables the synthesis of diheterocycles containing 1,2,4-traizolo[4,3-*a*] pyridine and quinoline moieties 102. The reaction utilizes 2-methyl quinoline derivatives 101 and 2-hydrazinepyridine derivatives 99 as starting materials and is facilitated by the I_2_/DMSO system (Scheme 60). In this protocol, the oxidative activation of the C–H (sp^3^) bond reacting with 2-hydrazinepyridine 99*via* (1 + 4 ring forming procedure) affords 1,2,4-traizolo[4,3-*a*] pyridine 102 in good yield. The compatibility of methyl quinoline 101 and 2-hydrazinepyridine 99, together with their derivatives substituted with diverse functional groups allows for their conversion into the desired products. According to the proposed reaction mechanism, molecular iodine is crucial in the transformation, which starts the reaction *via* iodination of methyl quinoline 101 followed by Kornblum oxidation, which results in the formation of quinoline-2-carbaldehyde, and it further reacts with 2-hydrazinepyridine 99*via* condensation, followed by the annulation to afford the target products (Scheme 61).

**Scheme 62:**
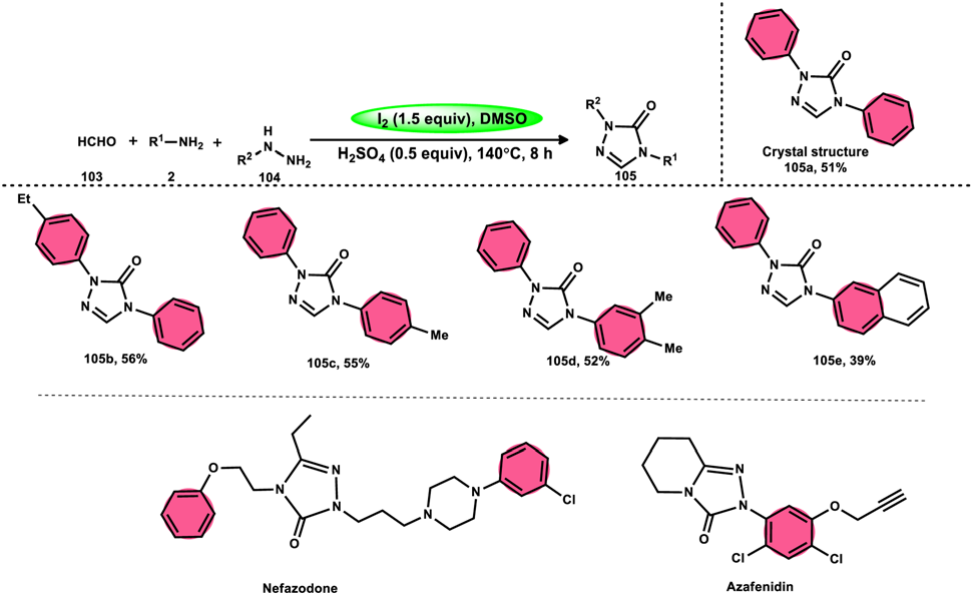
Synthesis of 2,4-disubstituted-1,2,4-triazole-3-ones 105, representative examples and their biologically active compounds.

Zhu *et al.*^102^ introduced a method to produce 2,4-disubstituted 1,2,4-triazole-3-ones 105 by a three-component reaction involving formaldehyde 103, amines 2, and hydrazines 104, which is facilitated by iodine and DMSO. 1,2,4-Triazole-3-ones 105 are significant heterocyclic compounds that exhibit a diverse range of biological functions.^103–105^ However, the reaction does not occur at a temperature of 100 °C and only produces a yield of 28%. Further, absence of I_2_ resulted in minimal product formation, highlighting the vital importance of I_2_ in this reaction. The desired product could not be obtained when anilines with strong electron-withdrawing substituents, such as nitro groups, were used as the reactants. Also, the desired product could not be obtained if the temperature is lower than 100 °C. Both I_2_ and DMSO are essential given that the lack of either resulted in a small or insufficient quantity of the desired product. Also, I_2_ must be introduced after all other reactants are added; otherwise, complicated products will be produced throughout the process. Alternatively, an atmosphere of either O_2_ or N_2_ did not affect the yield of the product (Scheme 62).

**Scheme 63:**
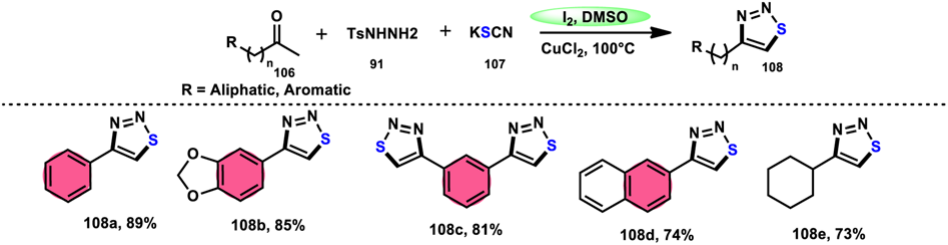
Iodine–DMSO-catalyzed synthesis of 1,2,3-thiadiazoles 108 and representative examples.

**Scheme 64:**
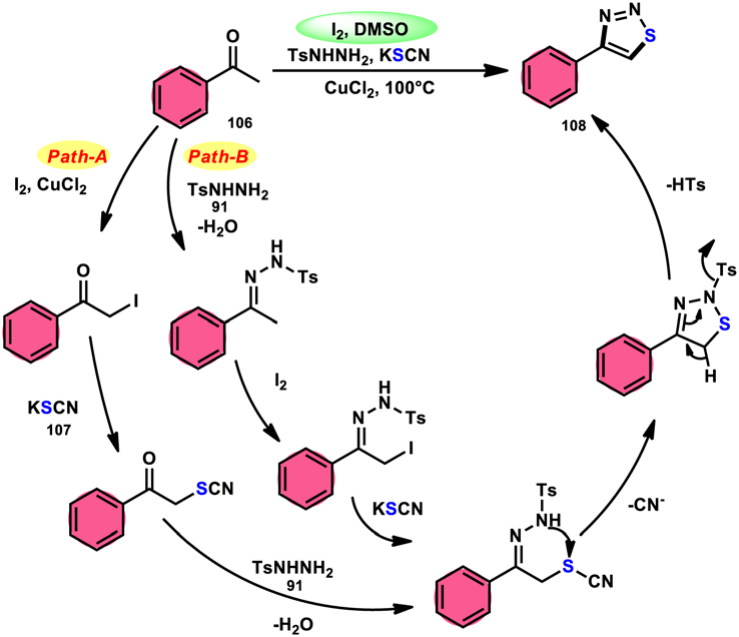
Iodine–DMSO-catalyzed mechanism for the synthesis of 1,2,3-thiadiazoles 108.

Wu *et al.*^106^ developed a concise and efficient method for the synthesis of 1,2,3-thiadiazoles 108 using a one-pot, three-component protocol. The reaction utilizes readily accessible and cost-effective reactants, including TsNHNH_2_91, KSCN 107, and aliphatic or aromatic methyl ketones 106. The reaction is facilitated by the use of I_2_/CuCl_2_ as a catalyst in DMSO solvent, as depicted in Scheme 63. The methodology exhibits a broad substrate scope, including aryl/heteroaryl/chained-aliphatic/cyclic aliphatic methyl ketones 106, which are capable of producing the desired compounds. Additionally, the methodology demonstrates good compatibility with various substituents on the aryl moiety of the aryl methyl ketones, resulting in high yields in the majority of cases. The conventional Kornblum oxidation of ketones to glyoxal derivatives was found to be incompatible with the proposed reaction mechanism. Instead, an alternative pathway was observed. Initially, an intermediate α-iodo ketone is formed, followed by substitution of the iodine atom with the nucleophile SCN^−^. Then, a condensation reaction between ketone 106 and TsNHNH_2_91 occurs, resulting in the formation of a C–N bond. Next, intramolecular cyclization leads to the desired product 108. Molecular iodine plays a key role in the methodology; however, the yield without the catalyst CuCl_2_ is lower, and thus CuCl_2_ increases the yield to an outstanding level (Scheme 64).

**Scheme 65:**
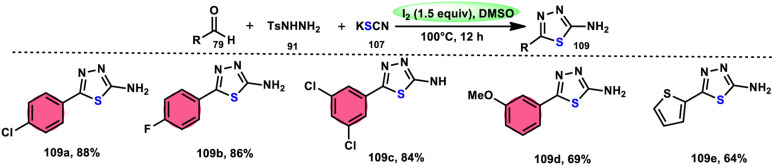
Synthesis of aminothiadiazoles 109 catalyzed by I_2_–DMSO and selected examples.

**Scheme 66:**
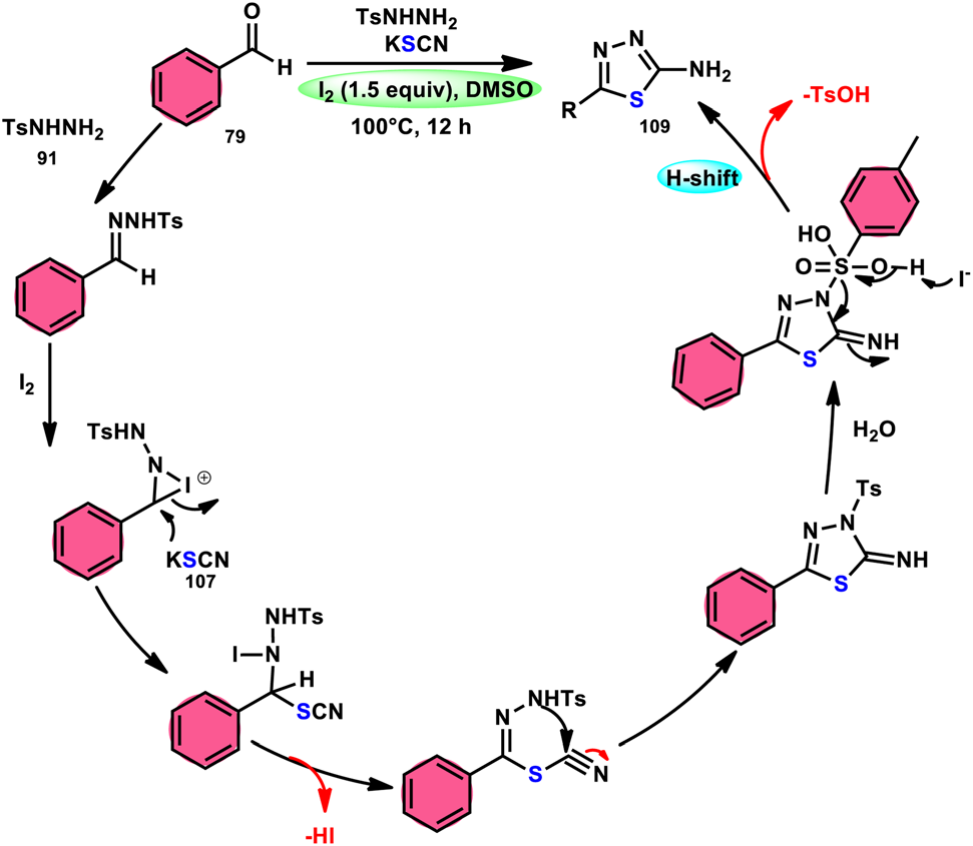
Mechanism for the synthesis of aminothiadiazoles 109 catalyzed by I_2_–DMSO.

Sen Lin *et al.*^107^ designed a unique protocol to synthesize 2-aminothiadiazole 109 employing aldehyde 79, NH_2_NHTs (*p*-toluenesulfonyl hydrazide) 91, and KSCN 107, which works as an odourless, effective source of S without emitting toxic cyanide byproduct (Scheme 65). The three-component, metal-free, I_2_/DMSO-mediated procedure proceeds in a one-pot fashion, producing the target product in satisfactory yields and on a gram scale. Benzaldehydes having EDGs or EWGs, multi-substituents or heteroaryl aldehydes are competent to afford the desired products; however, aliphatic substituents cannot be converted to the target product. According to the plausible reaction mechanism, aldehyde 79 reacts with TsNHNH_2_91 to afford *N-*tosylhydrazone, which reacts with iodine, resulting in an iodonium salt intermediate. Subsequently, ^−^SCN attacks this intermediate, and in the last step intramolecular cyclization results in the formation of an N–C bond, generating the desired product 109 (Scheme 66).

**Scheme 67:**
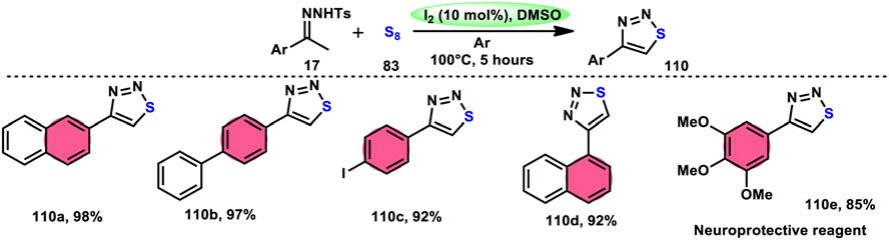
Synthesis of thiadiazoles 110 using I_2_/DMSO system.

**Scheme 68:**
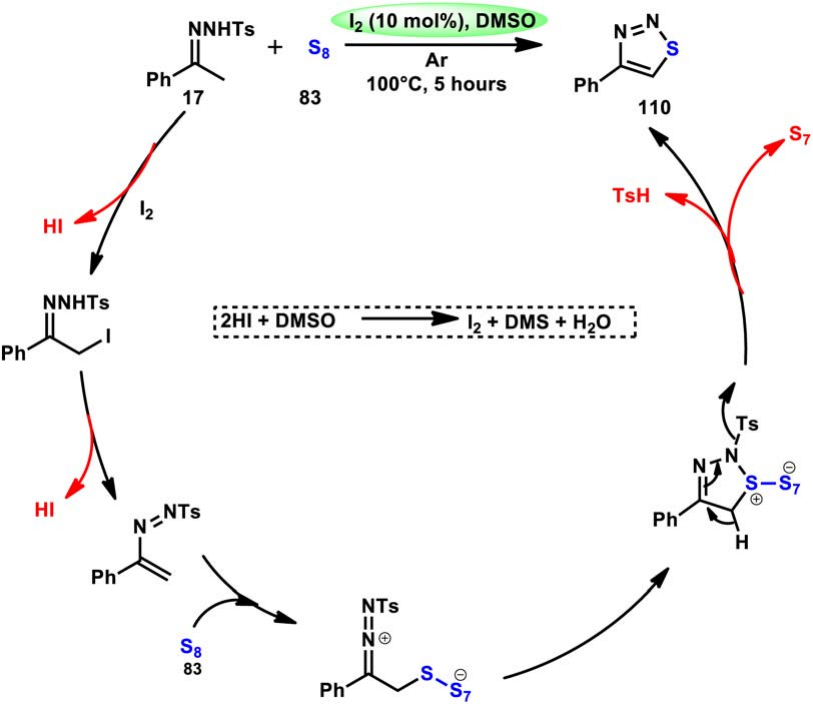
Mechanism for the synthesis of thiadiazoles 110 using I_2_/DMSO system.

Liu, Gu, and colleagues^108^ discussed the synthesis of 4-aryl-1,2,3-thiadiazoles 110. They described a selective cyclization process using iodine/DMSO as the catalyst, which enabled the cyclization of *N*-tosylhydrazones 17 with sulphur 83 without the need for an additional oxidant (Scheme 67). The crucial aspect of this procedure involves the oxidation of HI using DMSO as both the oxidizing agent and solvent. This enabled the retrieval of elemental iodine, thereby confirming the achievement of the synthesis. The distinguishing features of this protocol include its user-friendly nature, efficient utilization of steps (one-pot approach), wide range of applicable substrates, and potential for scalability. This methodology can be employed for the synthesis of compounds on a gram scale. A one-pot synthesis method was also developed, enabling the direct utilization of ketone as a precursor without the need for isolating *N*-tosylhydrazone intermediate 17. The efficacy of this method was also demonstrated in the production of neuroprotective drug 110e. The utilization of this approach offers a notable advantage given that it eliminates the need for the utilization of external oxidizing agents (Scheme 68).

## 9. Other reactions

### 9.1 Sulfenylation

**Scheme 69:**
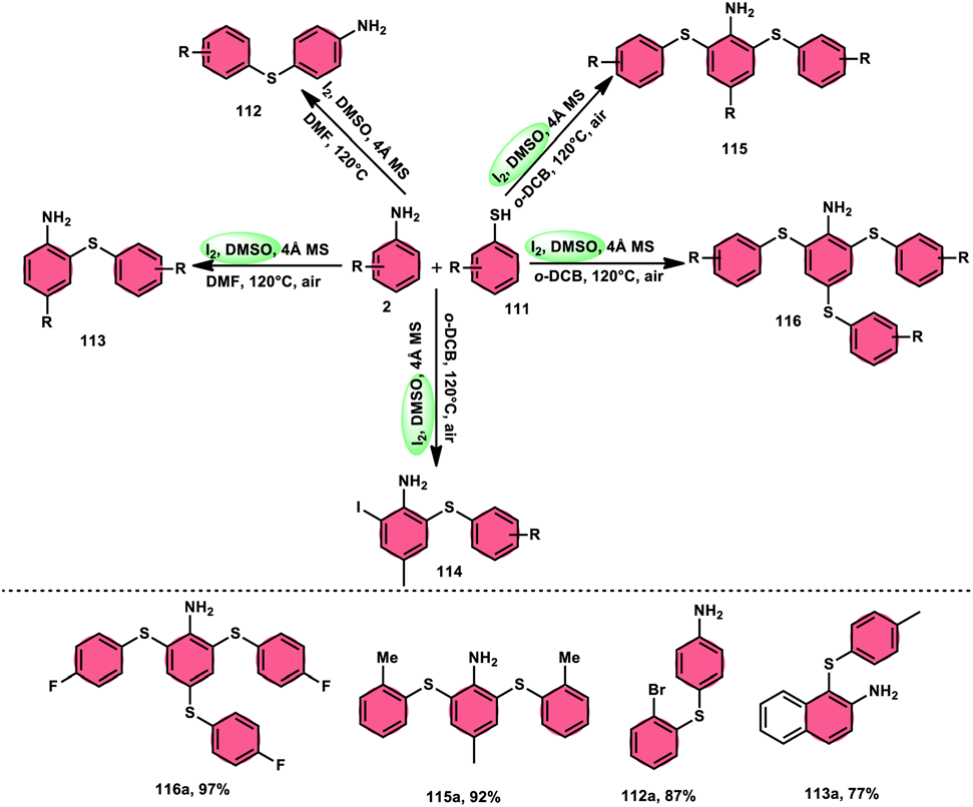
Reaction of aniline 2 with thiols 111 in a I_2_/DMSO environment.

The synthesis of sulfide anilines 112–116 was developed by Zhang, Deng, and research team. This method involves the iodine-promoted divergent thiolation of unprotected anilines 2 using thiols 111.^109^ The utilization of a mixture comprising I_2_ and DMSO played a significant role in facilitating this particular reaction, avoiding the use for a metal catalyst or potent oxidizing agents. To produce mono-112–113, bis-115, and trisubstituted diaryl sulphide derivatives 116, the reaction selectivity was carefully regulated. Significantly, the simultaneous iodination and sulfenylation processes can yield very useful iodoaniline molecules that possess several functional groups. Under the conditions of mild reactions, this methodology offers a successful procedure for synthesizing C–S and C–I bonds by the activation of C–H bonds. The oxidative reaction conditions utilizing DMSO as a solvent demonstrated favorable outcomes when applied to synthetically valuable functional groups, such as halogens. Given that DMSO is crucial in this thiolation reaction, the coupling reaction in its absence did not produce any products. The desired compounds were successfully synthesized in high yields through the reaction of aniline 2 with different thiol derivatives 111 possessing methyl and halogen substituents (–F, –Cl, and –Br) on the aromatic ring (Scheme 69).

**Scheme 70:**
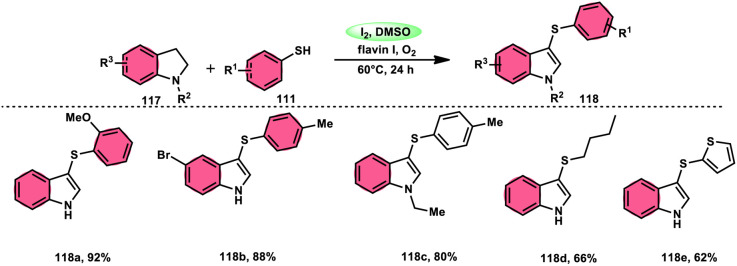
Formation of sulfenylindoles 118 using I_2_/DMSO framework.

Chuanming Yu and colleagues^110^ discovered a method for the aerobic oxidative sulfenylation of aryl-fused cyclic amines, using a range of thiols 111 and flavin/I_2_ as catalysts. The conversion facilitated by flavin II resulted in the replacement of the *para*-position on the aryl ring with a sulfenyl group, leading to the synthesis of 6-sulfenylquinolines. In contrast, flavin I was shown to act as a catalyst in the sulfenylation process of indolines, resulting in the synthesis of 3-sulfenylindoles 118. The investigation focused on the benefits of using ambient oxygen as the ultimate oxidizing agent in this metal-free oxidative C–S coupling method, which was conducted under ecologically sustainable conditions. The results demonstrated the significant atom efficiency and high degree of compatibility with various functional groups. This study represents the initial occurrence of a sequential process including the dehydrogenation and sulfenylation of indolines, employing thiophenols 111. The procedure presented in this study offers a metal-free approach for the production of environmentally sustainable water given that it is the only by-product, using molecular oxygen as the final oxidizing agent. The intended compounds were successfully synthesized in high yields by the incorporation of several electron-donating groups (–Me, –OMe, and –Ph) and electron-withdrawing groups (–F, –Cl, –Br, and –CF_3_) at different positions of thiophenol, as well as at the C-2, C-4, C-5, and C-6 positions of the indoline ring (Scheme 70).

**Scheme 71:**
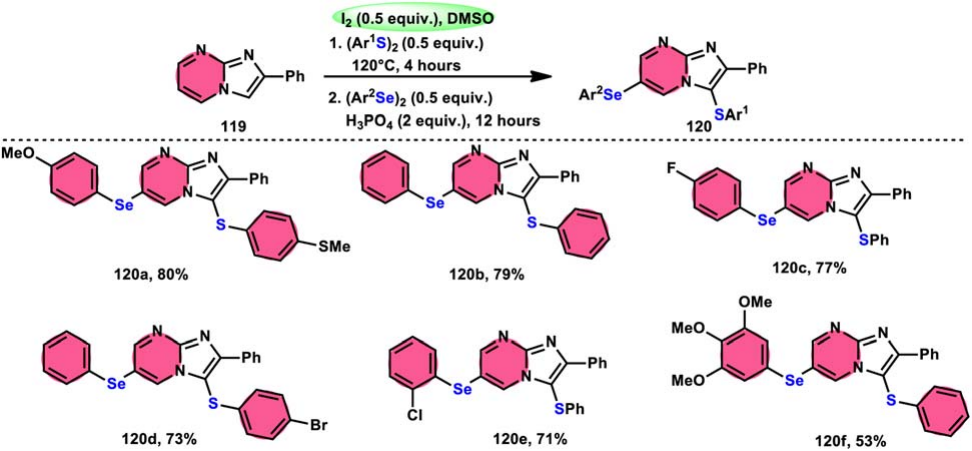
I_2_/DMSO-based dichalcogenation of imidazo[1,2-*a*]pyrimidine 120.

**Scheme 72:**
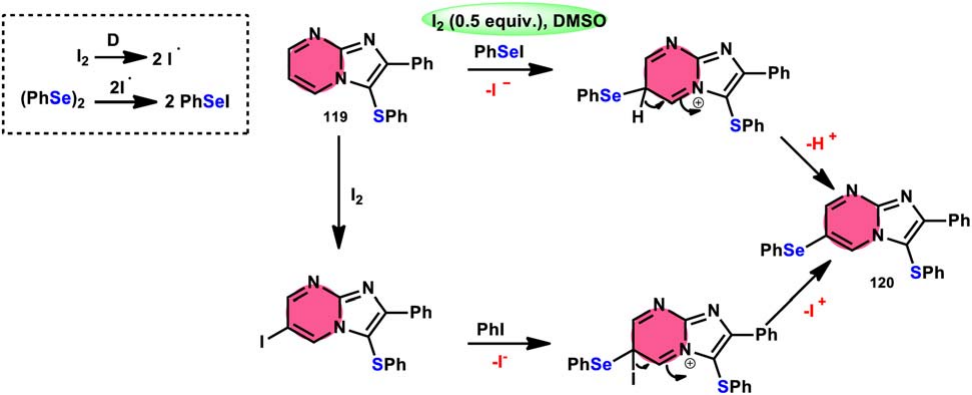
I_2_/DMSO-based dichalcogenation mechanism of imidazo[1,2-*a*]pyrimidine 120.

Vanelle, Redon, and colleagues^111^ documented the process of dichalcogenation of imidazoheterocycles 119, leading to the successful functionalization of the C6-position of the imidazo[1,2-*a*]pyrimidine moiety 119 (Scheme 71). The process of iodine/DMSO treatment of diaryldichalcogenides commenced with C3-chalcogenation, and then by C6-selanylation of 120, which was facilitated in acidic conditions using phosphoric acid. The addition of stronger acids proved the beneficial effect on the reaction efficiency. This unique stepwise dichalcogenation technique performed well in terms of regioselectivity and yield. However, the yield was considerably degraded (5%) under an inert atmosphere (N_2_), highlighting the importance of O_2_ in the process. The use of the C6-halogenated intermediate was shown to provide benefits in promoting cross-coupling events, which resulted in the formation of C–C bonds. The mechanistic studies suggested that C6-selanylation of 120 can be related to electrophilic aromatic substitution (Scheme 72).

**Scheme 73:**
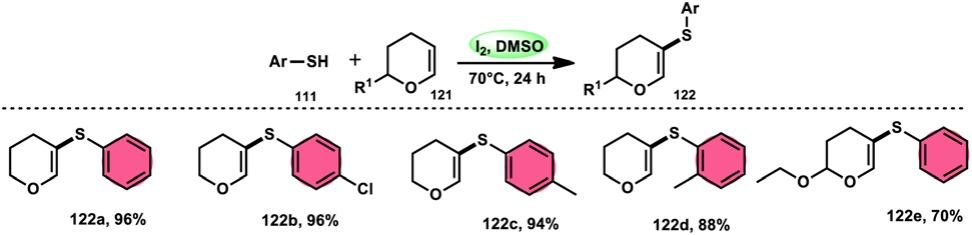
Thioarylation of dihydropyrans with different thiols 111.

**Scheme 74:**
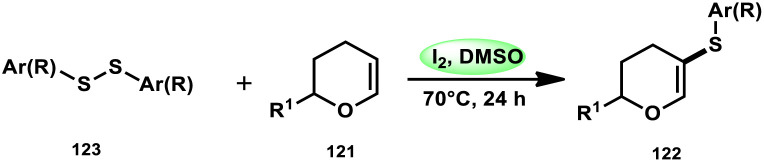
Thioarylation of dihydropyrans with disulfides 123.

**Scheme 75:**
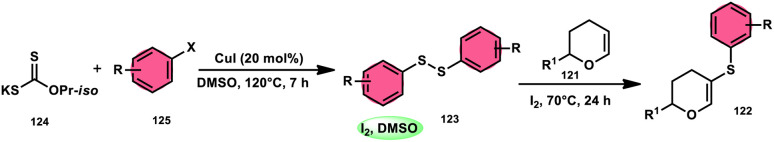
Thioarylation of dihydropyrans with aryl halides 125.

Nejmoh Nowrouzi *et al.*^112^ devised a series approach for the sulfenylation of the C(sp^2^)–H bond of dihydropyrans employing thiols 111, disulfides 123, and aryl halides 125 as co-reagents. The first methodology was carried out in one-pot, straightforward fashion, and without any metal-catalyst using dihydropyrans as the substrate and aromatic thiols or disulfides reactants promoted by the I_2_/DMSO combination (Schemes 73 and 74). In another repugnant-free methodology, aryl halide 125 was deployed instead of thiols or disulfides and CuI, potassium isopropyl, and molecular I_2_ worked as catalysts to achieve sulfonated dihydropyrans (Scheme 75). Under all these conditions, the yield was excellent, although aliphatic thiols and aryl chlorides were not potent to afford the desired products 122, and in addition, dihydropyrans bearing EWGs gave lower yields. I_2_ plays a central role in all these reactions, such as oxidizing thiols first to disulfides, and further converting them to ArSI, making it susceptible to nucleophilic attack by dihydropyran working as a good leaving group.

### 9.2 Decarbonylation

**Scheme 76:**
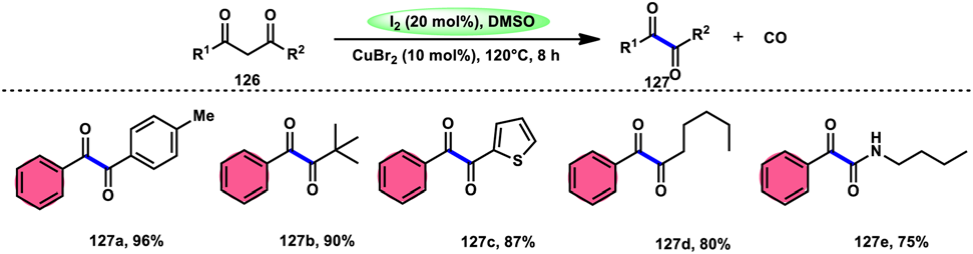
Synthesis of 1,2-dicarbonyl compounds 126 and selected examples.

**Scheme 77:**
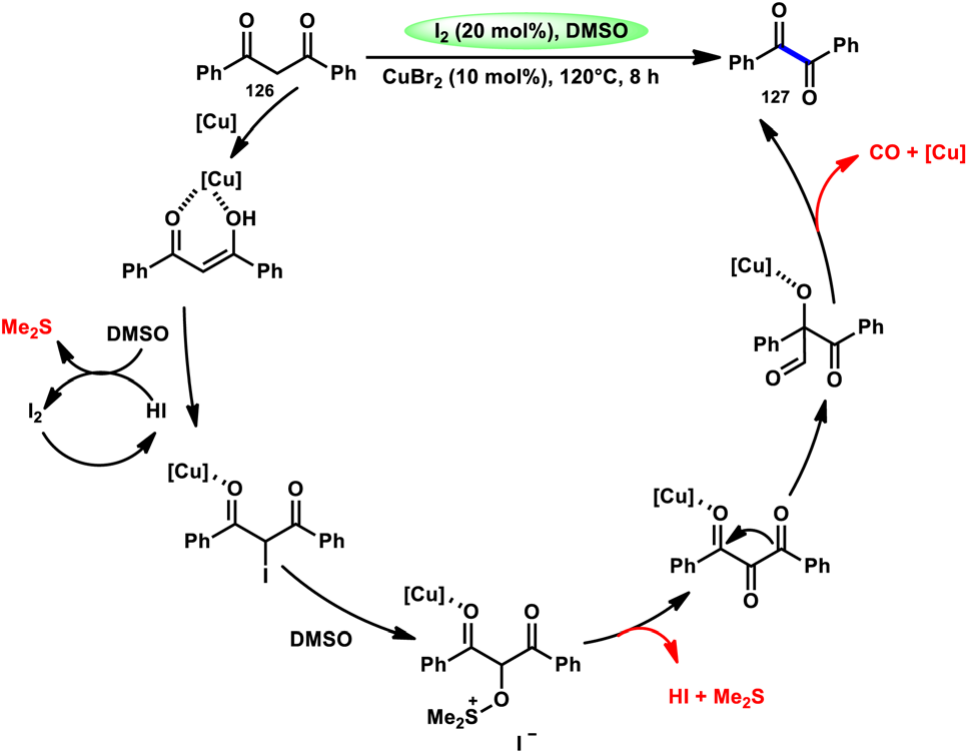
Mechanism for the synthesis of 1,2-dicarbonyl compounds 126.

For the oxidative C–C bond breakage of 1,3-dicarbonyl compounds 126, Chen and colleagues^113^ developed a coordinated catalytic system including CuBr_2_ and I_2_ (Scheme 76). The oxidant used in this process was DMSO, resulting in the formation of 1,2-dicarbonyl compounds 127, which hold considerable synthetic value. Significant selectivity was achieved in the breaking of the C–C bonds during the process of carbon monoxide (CO) release when 1,2-dicarbonyl molecules 127 were considered. Moreover, this specific methodology can be used for a wide range of 1,3-dicarbonyl compounds, involving 1,3-diketones, 1,3-keto esters, and 1,3-keto amides. The use of a copper catalyst to improve the reactivity of iodine affords a prospect for the investigation and progression of a novel theoretical approach for cleaving C–C bonds. The observed reaction exhibited a dependence on temperature, whereby a decrease in temperature from 120 °C to either 100 °C or 80 °C resulted in a reduction in the product yields. A variety of substituents, including –Me, –MeO, –Cl, –CO_2_Me, –F, and –CF_3_, was shown to be compatible with 1,3-dicarbonyl compounds. The reaction exhibited some sensitivity to both electronic and steric hindrance factors (Scheme 77).

**Scheme 78:**
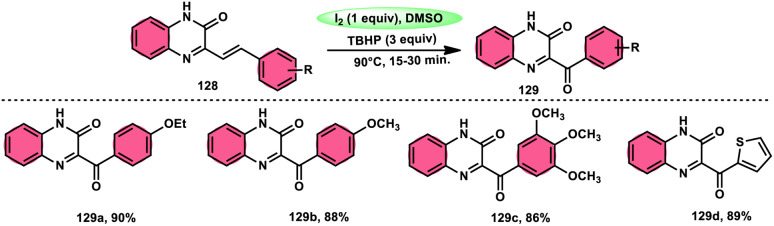
I_2_/DMSO-mediated metal-free reaction.

**Scheme 79:**
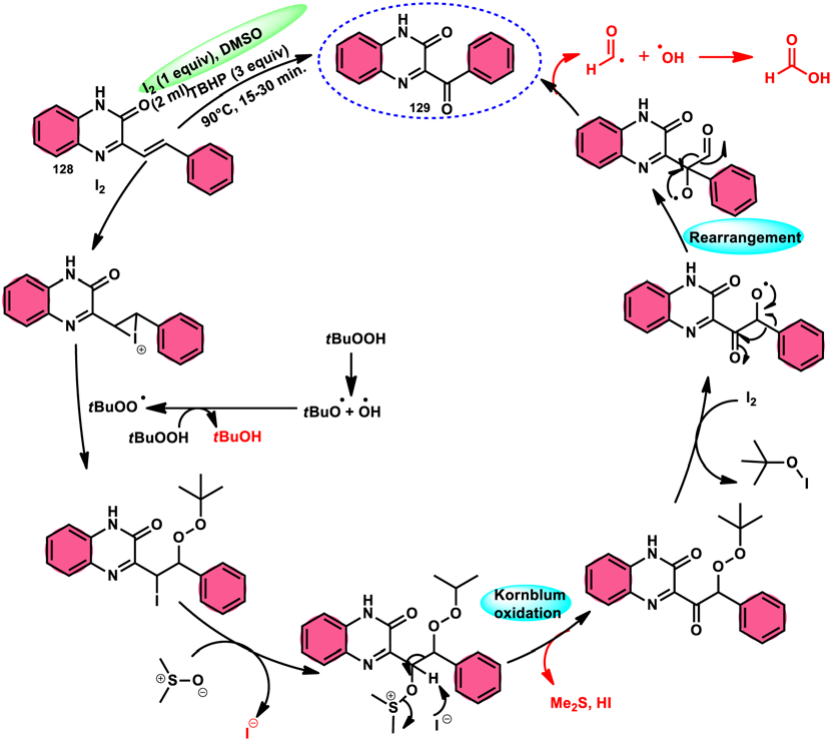
Mechanistic pathway for I_2_/DMSO-mediated metal-free reaction mechanism.

A new approach was developed by Kamal K. Kapoor *et al.*^114^ to produce 3-aroylquinoxaline-2(1*H*)-ones 112 (Scheme 78), which is both efficient and ecologically sustainable, while also being free of any metal components. The researchers used the combination of I_2_ and DMSO, together with *tert*-butyl hydroperoxide (TBHP) as a co-oxidant. This particular combination of I_2_/DMSO/TBHP is widely recognized for its effectiveness in facilitating the oxidative rearrangement of α,β-unsaturated ketones 111. The reaction protocol exhibits favorable tolerance towards a range of styrylquinoxalin-2(1*H*)-one substrates, resulting in satisfactory yields. It is worth mentioning that substrates including electron-donating groups exhibit greater yields in comparison to that incorporating electron-withdrawing groups. This discovery implies that the reaction initiates with the involvement of electron-rich olefinic groups as nucleophiles. According to the plausible reaction mechanism, the substrate initiates the reactions by attacking I_2_, generating the iodonium intermediate. The reaction further leads *via* Kornblum oxidation followed by a radical rearrangement. TBHP (tertiary butyl hydroperoxide) generates the radicals and the reaction continues in a radical manner, resulting in the desired product 112 through a carbon degrading reaction (Scheme 79).

### 9.3 α-Oxygenation and deoxygenation

**Scheme 80:**
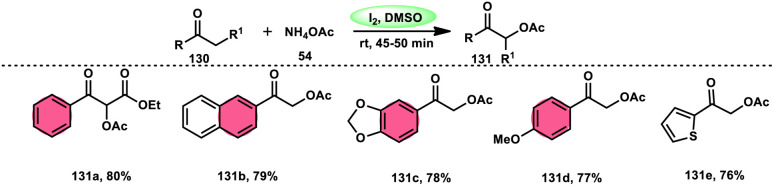
I_2_-mediated direct α-functionalization of ketones 113.

**Scheme 81:**
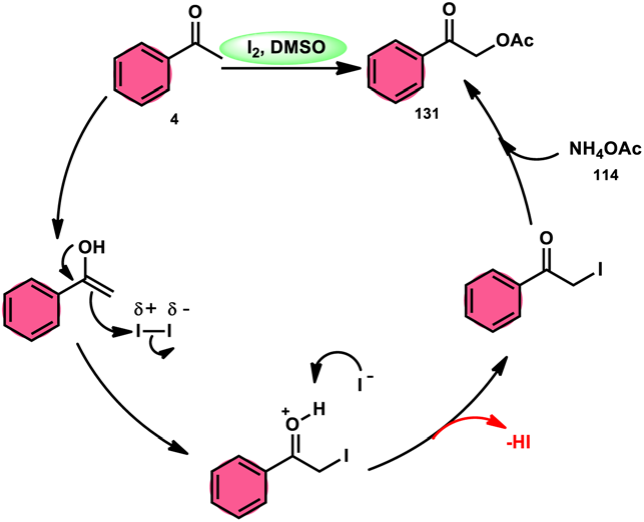
I_2_–DMSO-mediated mechanism of direct α-functionalization of ketones 130.

Prapurna *et al.* presented a procedure for the iodine-mediated oxidative α-functionalization of ketones 130 using NH_4_OAC 54. This method presents a direct method for the production of α-acetoxy and α-alkoxy ketones 131 using readily available chemicals (Scheme 80).^115^ The reaction begins with iodination, and then moves to nucleophilic substitution with acetate. The product was produced within one hour of the reaction and completed at room temperature. Under the specified reaction conditions, acetophenone, which has electronic substituents on its phenyl ring, exhibited favourable reactivity, leading to the synthesis of α-keto-acetals in modest yields. Nevertheless, the attempted reactions using cyclohexanone and ethylmethyl ketone did not provide the anticipated products. Consequently, the proposed reaction is a helpful alternative to conventional methods for α-oxygenation due to its simple synthesis method, broad substrate range, and quicker reaction times (Scheme 81).

**Scheme 82:**
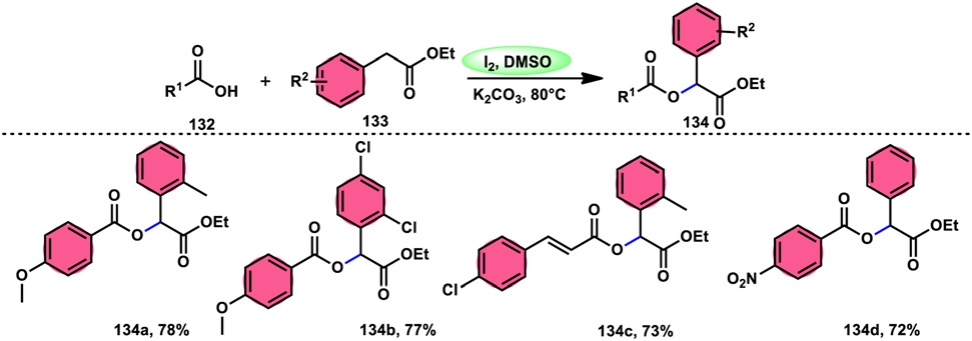
Synthesis of α-acyloxy esters 134 and representative examples.

A practical, economic, simple, transition-metal free method of cross dehydrogenative coupling (CDC) promoted by I_2_/DMSO was developed by the team of Krishan Nand Singh.^116^ The researchers synthesized α-acyloxy esters derivates 134 from the inexpensive easily accessible reactants aryl carboxylic acid 132 and ethyl arylacetates 133 in basic medium of K_2_CO_3_ in the appropriate yield. This methodology has good functional tolerance on both reactants without any effect from EDG and EWG groups. as Also, α,β-unsaturated aryl carboxylic acids were equally compatible; however, aliphatic esters could not produce the desired products. According to the reaction mechanism proposed by the investigators, the ester is converted into an α-iodo ester intermediate, and further a carboxylate ion (originates from the carboxylic acid by K_2_CO_3_) participates in nucleophilic substitution attack on the ester intermediate, leading to the desired product (Scheme 82).

**Scheme 83:**
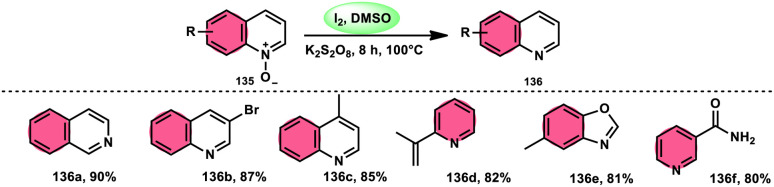
Deoxygenation of *N*-oxides 135 and representative examples.

**Scheme 84:**
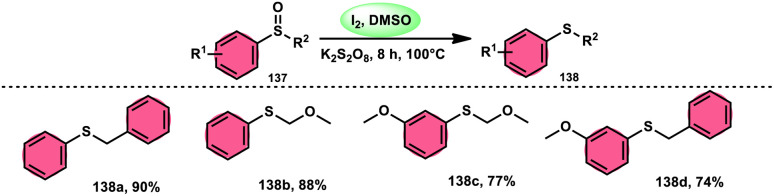
Deoxygenation of sulfoxides 137 and representative examples.

The I_2_/DMSO combo in presence of K_2_S_2_O_8_ was applied in a different approach to generate amine 136 and sulfides 138 from amine *N*-oxides 135 (Scheme 83) and sulfoxides (Scheme 84), respectively, by the team of Dushyant Singh Raghuvanshi.^117^ The metal-free, straightforward, deoxygenative technique has practical value owing to the use of the inexpensive and green I_2_/K_2_S_2_O_8_ catalyst, its excellent yields, good functional group tolerance, and broad substrate scope including pyridine *N*-oxides, secondary/tertiary amine *N*-oxides and aryl sulfoxides. However, the nitro group positioned at C-8 in quinoline was not reduced to the desired products and aliphatic sulfoxides were not compatible with this scheme. The molecular iodine plays a pivotal role in reacting such as an electrophile initiating the reaction, whereas homolysis activities occur with K_2_S_2_O_8_.

### 9.4 Oxidation

**Scheme 85:**
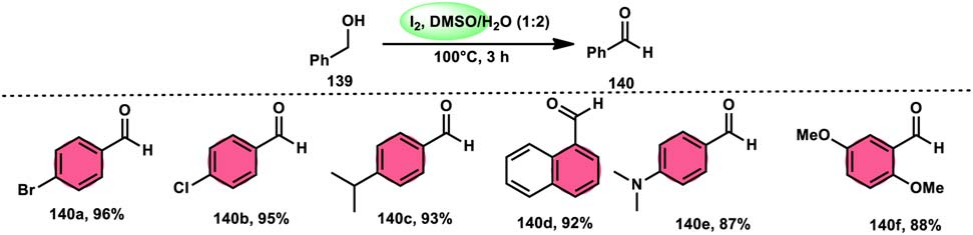
I_2_–DMSO-based chemoselective oxidation of benzylic alcohols 139.

A new, easy, low-cost, environmental-friendly and highly chemoselective and proficient protocol to access benzaldehyde 140 from benzyl alcohol 139 was designed by Ehsan Sheikhi and coworkers.^118^ Although many known methodologies are applied in the oxidation of the benzylic alcohol 139 to aromatic aldehyde 140, this tactic selectively oxidizes only benzylic alcohol 139, resulting in the formation of the desired product. This strategy has several advantages compared to previous methodologies such as no further purification of the product, no by-product, fascinating yields of up to 96%, no co-catalyst such as N_2_H_4_, K_2_CO_3_, MeCN and KI are required, where only the readily available DMSO dissolved in water (DMSO : H_2_O) in a ratio of 1 : 2 and molecular iodine are required (Scheme 85).

**Scheme 86:**
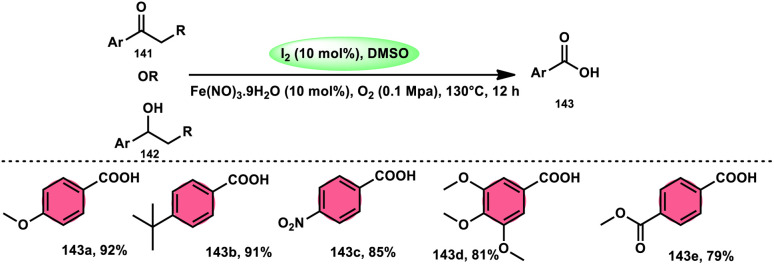
Oxidation of aryl methyl ketones 141 by I_2_/Fe(NO_3_)_3_·9H_2_O.

Li *et al.*^119^ devised an economically viable, uncomplicated, ecologically sustainable, and feasible methodology for synthesizing carboxylic acids 143. This method employs I_2_/Fe(NO_3_)_3_·9H_2_O as the catalysts and DMSO/O_2_ as the oxidants. This method utilized aryl alkyl ketones 141 or *sec*-benzylic alcohols 142 as feedstocks. It has good substrate scope, covering various substituted aryl alkyl ketones to heteroaryl alkyl ketones; however, to avoid polymerization reactions, the temperature must not be above 110 °C when heteroaryl is employed as the substrate. The approach used in this study demonstrates favourable to outstanding yields. However, it was noted that substrates containing electron-donating groups (EDGs) exhibit greater yields compared to substrates containing electron-withdrawing groups (EWGs). According to the precise analysis of the reaction mechanism by the researchers, initially, both types of substrates are converted into phenylglyoxal *via* Kornblum oxidation, whereas Fe^3+^ smoothly cleaves the C–C bond of phenylglyoxal, resulting in the formation of HCOOH as the by-product and benzaldehyde, which is oxidized by O_2_ to carboxylic acids. Interestingly, the use of N_2_ instead of O_2_ resulted in the disproportion of the ratio of desired products and by-products (Scheme 86).

### 9.5 Aromatization

**Scheme 87:**
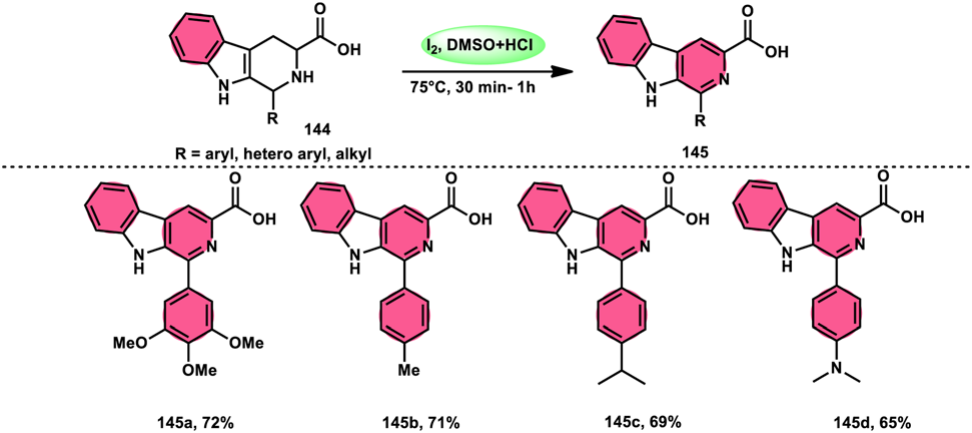
I_2_/DMSO-based chemoselective mechanism.

**Scheme 88:**
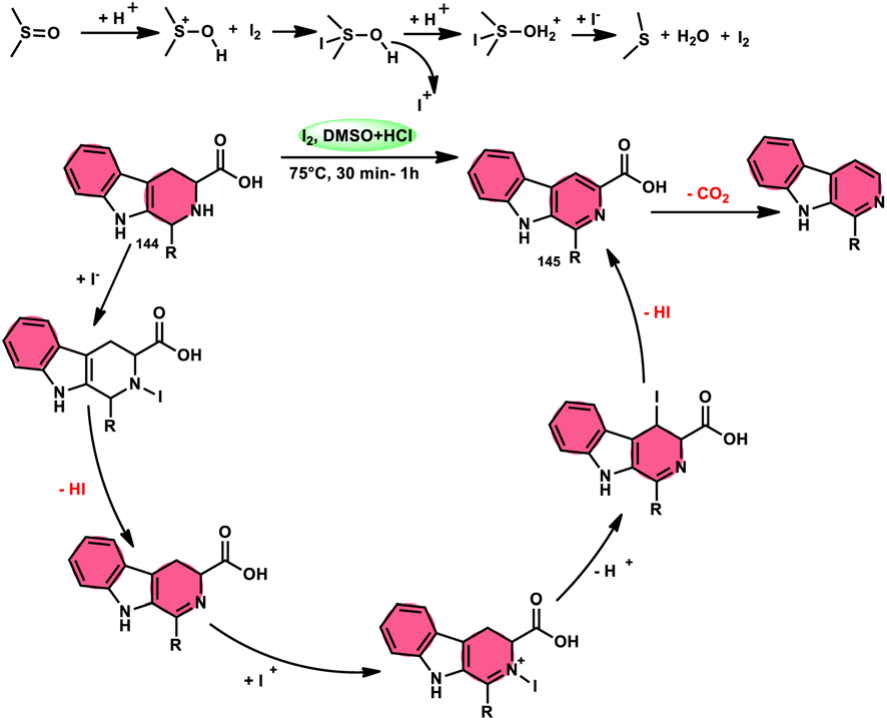
I_2_/DMSO-based chemoselective reaction and representative examples.

Bhosale, Lokhande, and colleagues^120^ developed a new catalytic method utilizing I_2_/DMSO for the selective aromatization of tetrahydro-β-carboline-3-carboxylic acids (THβC-carboxylic acids 144), aiming to produce β-carboline-3-carboxylic acid 145 (Scheme 87). This approach has the advantage of utilizing easily accessible starting materials. This study presents a metal-free aromatization methodology that includes a controlled decarboxylation phase. The developed approach offers several advantages, including mild reaction temperatures, convenient handling, and the utilization of readily accessible chemicals. In comparison to conventional approaches, using I_2_/DMSO to produce HI and I^+^ is a more environmentally friendly process. Additionally, this technique can be improved to aromatize new hybrid β-carboline compounds 145 and THβC methyl esters with great yield. Density functional theory (DFT) calculations were employed to investigate the process in a cost-effective manner. The chemo selectivity of β-carboline-3-carboxylic acid 145 was further validated using computational analysis. This research revealed that the carboxylic acid group was retained, leading to the significant production of β-carboline-3-carboxylic acid as the primary product 145 (Scheme 88).

**Scheme 89:**
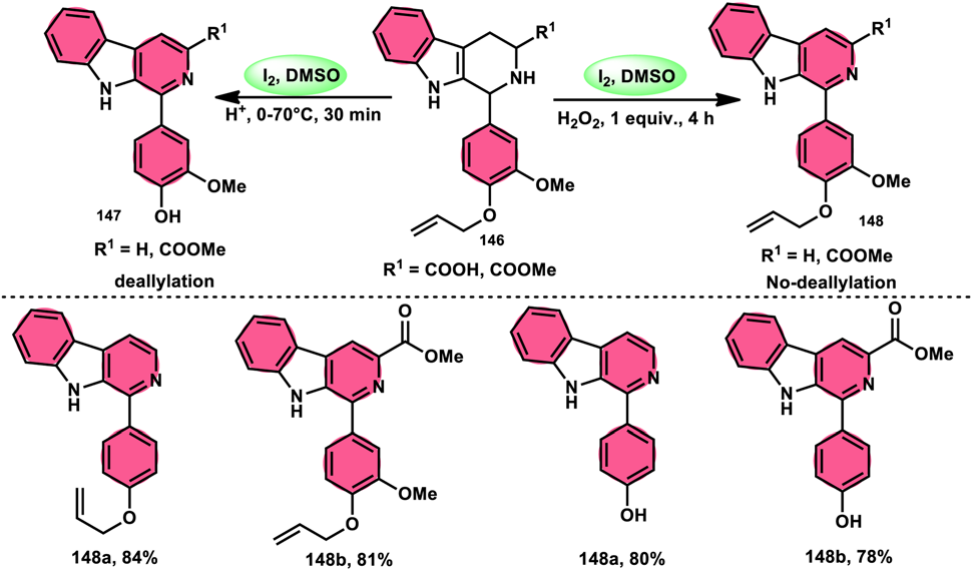
Deallylation of O-allyl tetrahydro-β-carboline 146.

Sunil V. Gaikwad and others^27^ designed a metal-free, green, economical, fast, and low temperature-based approach for the chemoselective aromatization tetrahydro-β-carboline (THβC) 147, as well as providing the option of obtaining deallylated or non-deallylated 148 of THβC. Catalyst I_2_ at 100% mol, DMSO as an oxidant, and H_2_O_2_ as an external oxidant all contributed to the high yield of the desired products. This methodology was employed to successfully synthesize flavones and has high functional tolerance, demonstrating its usefulness. The deallylation work-up occurred by adding just a drop of HCl to the reaction mixture. Overall, this strategy was proven to be superior to the conventional methods owing to its eco-friendly nature, low cost, shorter reaction time, and practical value (Scheme 89).

**Scheme 90:**
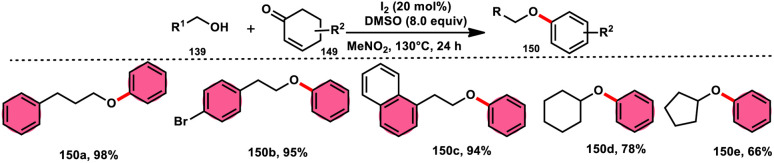
Coupling of cyclohexenones 149 and alcohols 139 catalyzed by I_2_–DMSO.

**Scheme 91:**
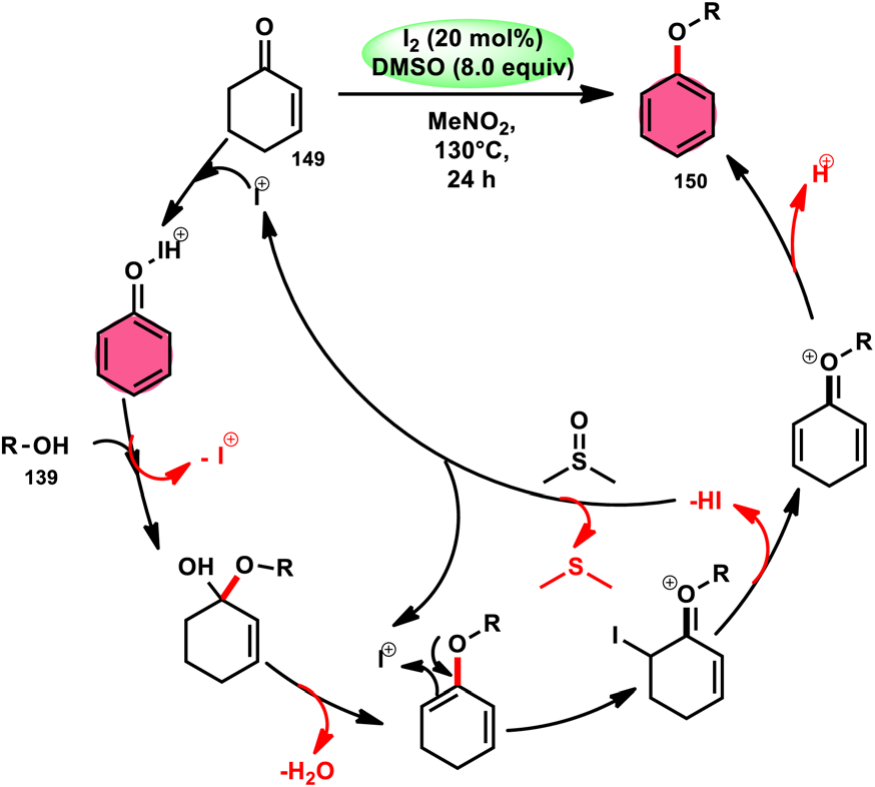
I_2_–DMSO-catalyzed mechanism of the reaction.

The synthesis of aryl alkyl ethers 150 through a new, metal-free, simple, and economical protocol was possible by employing commercially available nonaromatic cyclohexenones 149 and alcohols 139 (Scheme 90). In this study Jiao and colleagues^121^ used an approach using I_2_/DMSO, whereby molecular iodine serves as the catalyst and DMSO acts as the oxidant, facilitating the regeneration of I_2_ from I^−^. The broad substrate scope of alcohols 139 and compatibility of cyclohexenones 149 bearing various substituents to afford the desired products show the excellent substrate and functional group tolerance of this methodology. Besides, *meta*-substituted aromatic ethers are smoothly prepared using this strategy, which is tedious by conventional methods. According to the proposed reaction mechanism, iodine enhances the electrophilicity of the carbonyl group, leading to the aromatization of cyclohexenone 149*via* iodination; however, the reaction mechanism requires further investigation (Scheme 91).

### 9.6 Amidation

**Scheme 92:**
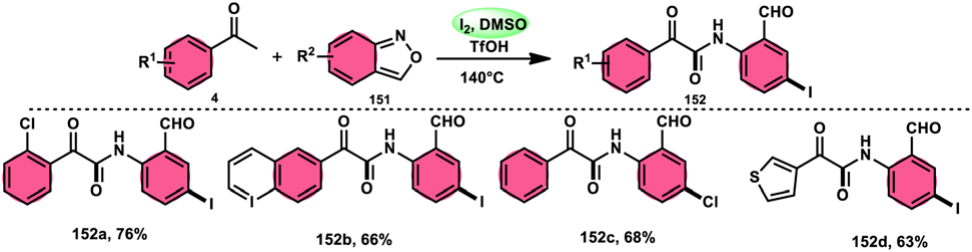
I_2_/DMSO-catalyzed synthesis of α-ketoamides 152 and representative examples.

**Scheme 93:**
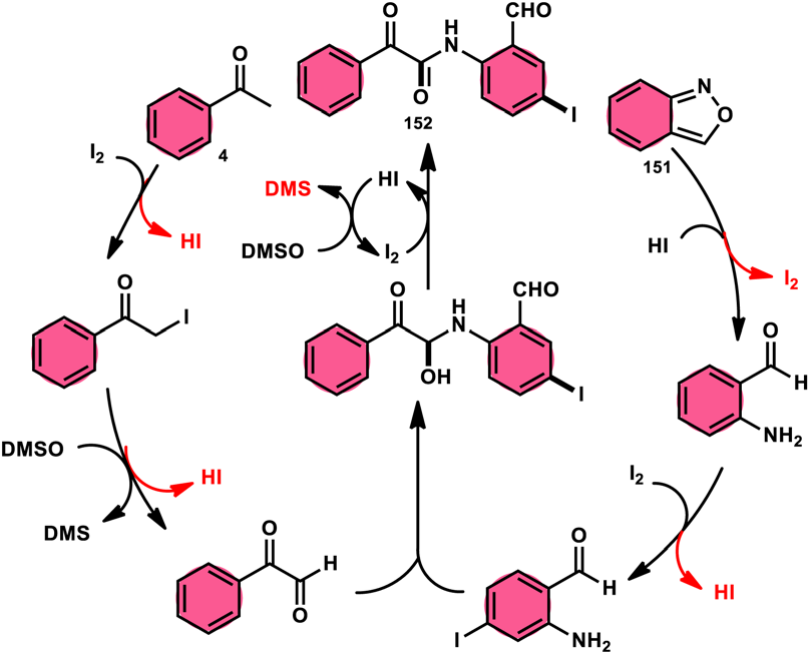
I_2_/DMSO-catalyzed mechanism of α-ketoamides 152.

The oxidative amidation of the methyl ketone to obtain α-ketoamides 152 in a unique, effective, atom-economical, and metal-free manner was developed by An-Xin Wu and coworkers (Scheme 92).^122^ This methodology promoted by I_2_/DMSO employs methyl ketones 4 and anthranils 151 as feedstock where anthranils 151 works as a masked N-nucleophile, with the addition of trifluoromethanesulfonic acid (TfOH) enhance the yield to a satisfactory level. Iodine plays a vital role in transforming the methyl ketone to phenylglyoxal *via* Kornblum oxidation and *in situ* generating 2-amino-4-iodobenzaldehyde to form anthranils *via* reductive ring opening besides iodination of this intermediate at 4-position. This process exhibits favourable functional group tolerance and a wide range of substrates in relation to anthranils and methyl ketones. However, it was observed that methyl ketones with electron-donating groups (EDGs) yielded higher quantities compared to ketones with electron-withdrawing groups (EWGs) on aryl rings. Regrettably, aliphatic ketones are not viable for producing the required products. This methodology provides further scope for the transformation of good target products bearing iodo and formyl groups. The iodo and formyl groups of the desired product *N*-(2-formyl-4-iodophenyl)-2-oxo-2-phenylacetamide further extend the scope of various important transformation (Scheme 93).

**Scheme 94:**
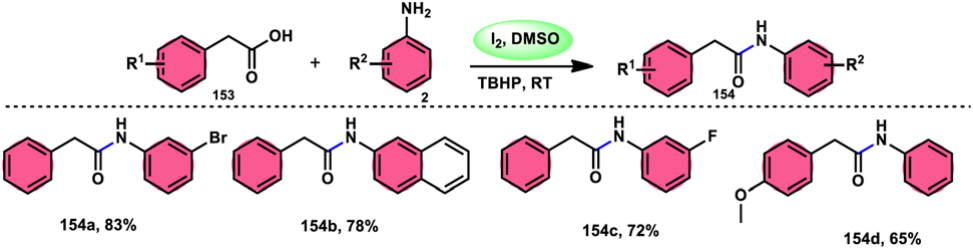
I_2_–DMSO-catalyzed synthesis of amides 154 and representative examples.

**Scheme 95:**
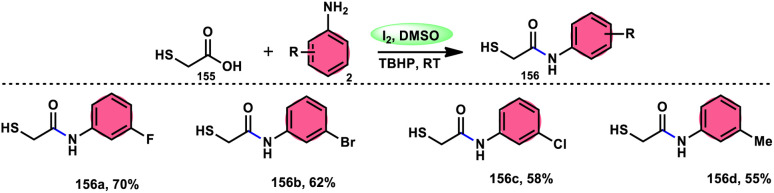
Synthesis of amides 156 using thioglycolic acids 155.

Sakram Boda *et al.*^123^ devised a facile, metal-free, environmental-benign technique to afford amide employing I_2_/TBHP as a catalyst in DMSO solvent. The reaction proceeds under mild reaction conditions utilizing carboxylic acid 153 and amines 2 as feedstock. Additionally, this methodology has a broad substrate scope covering aliphatic, aryl, bulky carboxylic acids and amines substituted with various functional groups (Scheme 94), and even thioglycolic acids 155 and phenoxy acetic acids were well tolerated in the reaction (Scheme 95). The feasible reaction starts by the reaction of TBHP and I_2_, forming the tertiary-butoxy radical, which abstracts H from acid and generates a carboxy radical, further attacking I_2_ to generate acetic hypoiodous anhydrides (ArCOOI). In conclusion, amine nucleophilic attacking acetic hypoiodous anhydrides generate amide 154 and HOI 156 as the final products.

### 9.7 Selenization

**Scheme 96:**
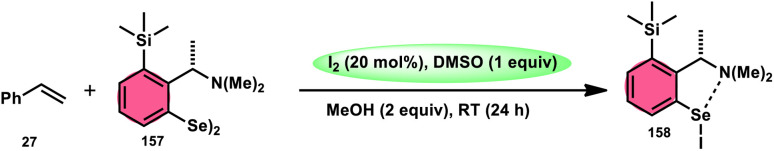
N–Se–I interactions detected by ORTEP analysis.

**Scheme 97:**
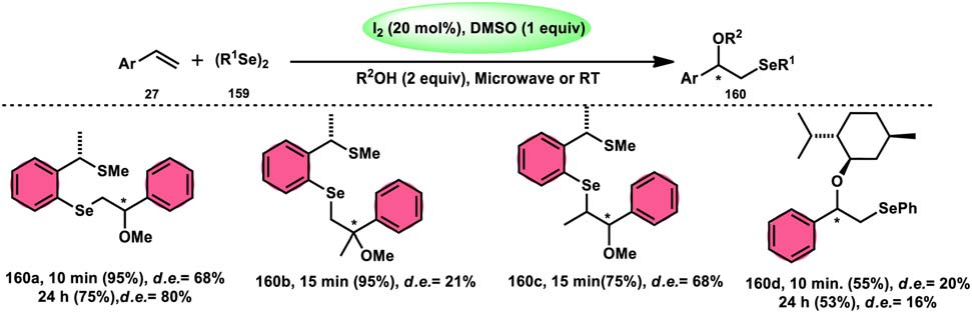
I_2_–DMSO-catalyzed alkoxy-selenization of styrenes 27.

The emerging catalytic combo I_2_/DMSO was applied by Julian B. Azeredo and coworkers^124^ to pursue the asymmetric alkoxy-selenization of styrene 27 and its derivatives in a metal-free, solvent-free, and environmental-benign methodology. These researchers detected that [(1*S*)-1-(methylthio)ethyl]phenyl diselenide 157 was the most effective non-racemic chiral diselenide reagent, and methanol works as the best alcohol, although menthol behaving as an alcohol source also shows feasibility of using natural products. The investigating team revealed that the high yield and diastereoisomeric excess resulting from [(1*S*)-1-(methylthio)ethyl]phenyl diselenide were attributed to the interaction between the S and Se atom, which brings the stereogenic center closer to the reaction center to form R–Se–I complex. It was further proven by ORTEP analysis that employing the diselenide reagent bearing an N-atom results in the formation of the N–Se–I complex (Scheme 96). The Se atom behaving as an electrophilic center directs the protocol in a Markovnikov regiochemistry manner, producing asymmetric alkoxy-selenized 160. The targeted products could be achieved using either microwave irradiation or heat as the energy source, providing equal results (Scheme 97).

**Scheme 98:**
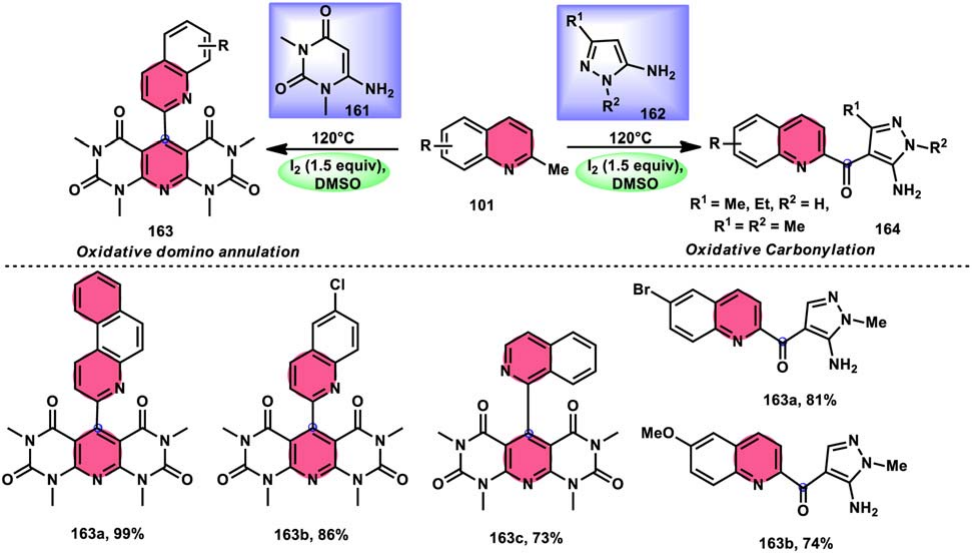
Synthesis of substituted pyridines 163 and diheteroaryl ketones 164.

In the research by Zhu, Sun, and colleagues,^125^ they developed a new approach employing an iodine-promoted oxidative domino annulation and carbonylation methodology. The use of this approach facilitates the production of physiologically significant aza-arene-substituted bis pyrazolo pyridines (BPPs) 163, *o*-amino diheteroaryl ketones 164, and diuracilpyridines (Scheme 98). Using readily available 5-aminouracils 161, modified 5-aminopyrazoles 162, and methyl aza-arenes 101, the domino process was carried out. This basic approach does not require the use of metals and it works well with numerous different substrates and functional groups. Additionally, this process can be exploited to synthesize gram-scale dipyrazolo/diuracil-fused pyridines 163 and 164. Substituted methyl quinolines incorporating both electron-donating and electron-removing groups exhibit a remarkable degree of tolerance.

**Scheme 99:**
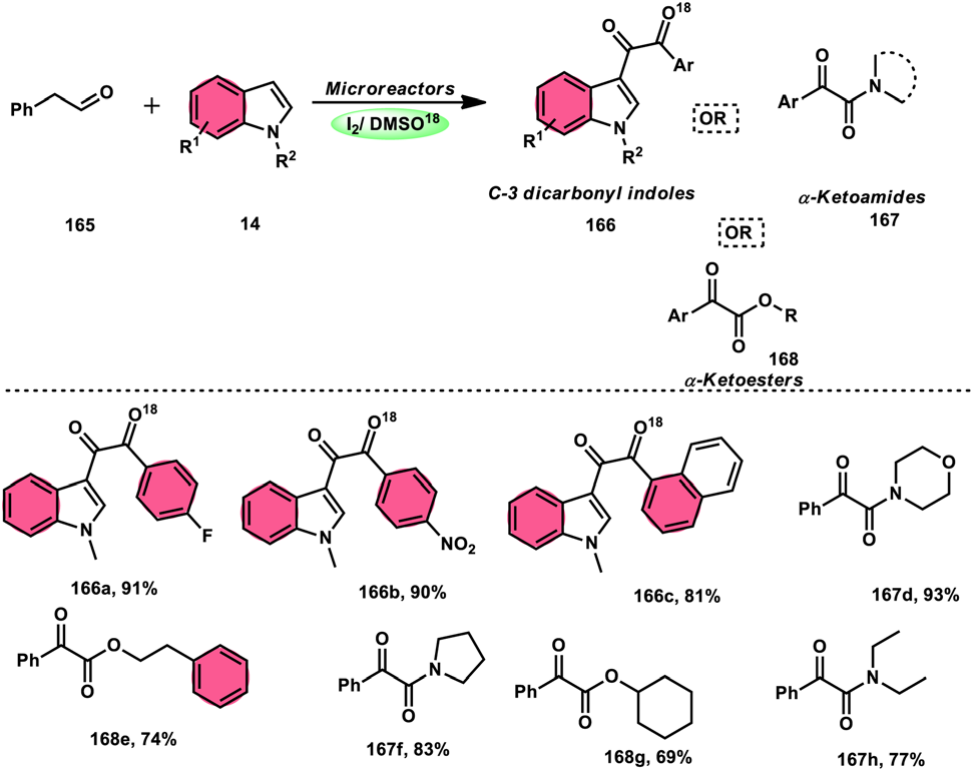
Selective oxidative coupling reactions facilitated by I_2_/DMSO.

Zheng Fang, Kai Guo and coworkers^126^ developed a practical, environmentally friendly, fast, metal- and amine catalyst-free, two-step continuous flow reaction strategy to prepare C-3 dicarbonyl indole derivates 166 from phenylacetaldehyde 165 and *N*-methyl indole 14 promoted by I_2_/DMSO. This approach also offers potential for synthesizing α-ketoamides^127^167 and α-ketoesters 168 simultaneously under the same reaction conditions, utilizing secondary amines and alcohols, respectively, which demonstrates its broad application. The reaction occurs in a two-step process within two microreactors to achieve a maximum yield of up to 91%. In the first step, phenylacetaldehyde 165 undergoes oxidation to form phenylglyoxal through the Kornblum oxidation method. In the subsequent step, indole is continuously introduced *via* a syringe, where it reacts with the iodine-activated aldehyde of phenylglyoxal to form a benzoin intermediate. This intermediate is then oxidized to yield the desired product. The research team employed a DMSO^18^-labelled experiment to establish the reaction mechanism. The methodology has good functional group tolerance for the substrates, where EDGs and EWGs are equally tolerated on both substrates; however, primary amine and aniline could not be converted into α-ketoamide 167 (Scheme 99).

## 10. Conclusion

This review presented the broad applicability of I_2_/DMSO working not merely as green oxidative agent but also a versatile catalyst–solvent combination having good compatibility for the formation of various important and diverse bonds, providing more beneficial paths to generate various important compounds. This review also highlighted the recent trends of employing transition metals, Fe/Cu, *etc.* as co-catalysts, which enables more proficient methodologies in achieving various significant compounds. Besides, I_2_/DMSO working as a solvent-free catalytic system is equally effective in enabling the formation of C–Se bonds under metal-free reaction conditions. Overall, this review will help readers understand the tendencies, reaction mechanism, and applicability of I_2_/DMSO without any metal catalyst or in the presence of metal catalysts. In this work, the synthetic progress made possible by the combination of DMSO and molecular iodine was highlighted. The majority of the biologically active compounds was produced synthetically, and these developments have had a significant impact on their preparation. Thus, the I_2_/DMSO catalytic system is anticipated to be used for the synthesis of significant structures and frameworks with biological activities in the future. This will enhance the application value of heteroatomic molecules in medicine, agricultural chemicals, materials, and fine chemicals. Nevertheless, the I_2_/DMSO catalytic system typically requires an elevated reaction temperature and its reaction activity is subject to certain limitations. Accordingly, further research is required to enhance these synthetic approaches for the production of new molecules with distinct pharmacological properties. For instance, the appropriate incorporation of certain additives may induce a reaction at standard ambient temperature.

Also, additional thorough studies are required to further enhance the reaction system and develop more effective catalytic reactions. In the future, it is anticipated that the use of I_2_/DMSO, an environmentally friendly catalytic system, will be expanded in organic synthesis to create a variety of beneficial heterocyclic compounds with biological properties.

## Conflicts of interest

There are no conflicts to declare.
